# Competitive molecular docking approach for predicting estrogen receptor subtype α agonists and antagonists

**DOI:** 10.1186/1471-2105-15-S11-S4

**Published:** 2014-10-21

**Authors:** Hui Wen Ng, Wenqian Zhang, Mao Shu, Heng Luo, Weigong Ge, Roger Perkins, Weida Tong, Huixiao Hong

**Affiliations:** 1 Division of Bioinformatics and Biostatistics, National Center for Toxicological Research, U.S. Food and Drug Administration, 3900 NCTR Road, Jefferson, AR 72079, USA; 2 University of Arkansas at Little Rock/University of Arkansas for Medical Sciences Bioinformatics Graduate Program, Little Rock, Arkansas, AR 72204, USA

## Abstract

**Background:**

Endocrine disrupting chemicals (EDCs) are exogenous compounds that interfere with the endocrine system of vertebrates, often through direct or indirect interactions with nuclear receptor proteins. Estrogen receptors (ERs) are particularly important protein targets and many EDCs are ER binders, capable of altering normal homeostatic transcription and signaling pathways. An estrogenic xenobiotic can bind ER as either an agonist or antagonist to increase or inhibit transcription, respectively. The receptor conformations in the complexes of ER bound with agonists and antagonists are different and dependent on interactions with co-regulator proteins that vary across tissue type. Assessment of chemical endocrine disruption potential depends not only on binding affinity to ERs, but also on changes that may alter the receptor conformation and its ability to subsequently bind DNA response elements and initiate transcription. Using both agonist and antagonist conformations of the ERα, we developed an *in silico *approach that can be used to differentiate agonist versus antagonist status of potential binders.

**Methods:**

The approach combined separate molecular docking models for ER agonist and antagonist conformations. The ability of this approach to differentiate agonists and antagonists was first evaluated using true agonists and antagonists extracted from the crystal structures available in the protein data bank (PDB), and then further validated using a larger set of ligands from the literature. The usefulness of the approach was demonstrated with enrichment analysis in data sets with a large number of decoy ligands.

**Results:**

The performance of individual agonist and antagonist docking models was found comparable to similar models in the literature. When combined in a competitive docking approach, they provided the ability to discriminate agonists from antagonists with good accuracy, as well as the ability to efficiently select true agonists and antagonists from decoys during enrichment analysis.

**Conclusion:**

This approach enables evaluation of potential ER biological function changes caused by chemicals bound to the receptor which, in turn, allows the assessment of a chemical's endocrine disrupting potential. The approach can be used not only by regulatory authorities to perform risk assessments on potential EDCs but also by the industry in drug discovery projects to screen for potential agonists and antagonists.

## Background

The endocrine system comprises a large system of glands that secrete hormones into the circulatory system where they travel to and exert their effects in target cells throughout the organism. The system plays pivotal roles in the regulation of homeostasis, growth and development as well as in a wide range of other normal bodily functions [1]. At the site of action, hormones exert their biological effects through highly complex and integrated signaling pathways which often involve the hormone receptors. Chemicals can alter endocrine function through a variety of molecular mechanisms, some of which involves these receptors, resulting in a wide spectrum of developmental and disease outcomes [2,3].

The terms endocrine disruptor or endocrine disrupting chemicals (EDCs) were coined in the early 1990s [4] following increasing concerns and awareness among the scientific community and public on the deleterious health effects caused by these compounds. The World Health Organization defined EDCs as "exogenous substances that alter function(s) of the endocrine system and consequently cause adverse health effects in an intact organism, or its progeny, or (sub)-populations", and potential EDCs as those chemicals that "possess the properties that might be expected to lead to endocrine disruption" [5]. A significant portion of the chemicals humans are exposed to on a daily basis are among the putative EDCs. They are found in drinking water as effluents from industry and agriculture [6,7]. Pharmaceutical [8], pesticide [9], plasticizer [10] and natural plant compounds such as phytoestrogens [11] are among the wide range of EDC sources. EDCs span an enormous range of chemical structure classes, and have the potential to cause a wide range of adverse health effects, where the developing organism is particularly sensitive [12,13], including stillbirths [14] and malformations of reproductive organs [8]. EDCs have also been implicated in a wide range of other adverse health effects including infertility or reduced fertility, precocious puberty, various cancers (e.g. breast [15,16], cervical and vaginal cancers [17-19]), obesity, diabetes, cardiovascular [20,21], and immune disorders[22], among others.

In response to growing evidence and concerns, the U.S. government moved swiftly to develop screens to detect potential EDCs, e.g. the Endocrine Disruptor Screening Program (EDSP) (http://www.epa.gov/endo/pubs/edspoverview/chronology.htm) spearheaded by the Environmental Protection Agency (EPA) [23,24]. The Food and Drug Administration (FDA) had also developed a number of databases, including the Endocrine Disruptor Knowledge Base (EDKB) [25], in the mid-1990s, and the more recent Estrogenic Activity Database (EADB) [26] as resources for the study of EDCs. Apart from that, a new guidance document on endocrine disruption potential of drugs had also been published by the FDA to monitor EDCs in pharmaceutical products (http://www.fda.gov/downloads/drugs/guidancecomplianceregulatoryinformation/guidances/ucm369043.pdf ).

**Figure 1:**
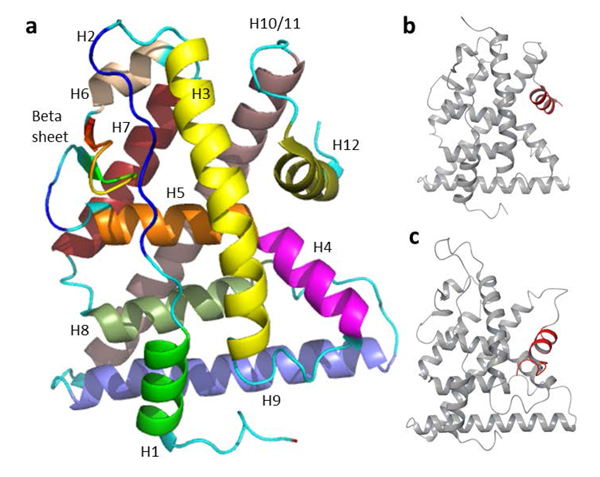
**Estrogen receptor ligand binding domain**. 1a The ER LBD comprising twelve α-helices and a beta sheet/hairpin: The twelve α-helices (H1-12) are colored differently for clarity; **1b **conformation of an active ER and **1c **conformation of an inactive ER. The major difference between 1b and 1c lies in the H12 conformation, highlighted in red.

Many hormone receptors are members of the nuclear receptor superfamily which modulate various endocrine mechanisms, often through acting as transcription factors, regulating gene expression involving development, homeostasis and metabolism [27]. The estrogen receptors (ERs), particularly the ERα subtype, have been extensively studied with substantial evidence accumulated of altered endocrine function through binding to xenoestrogens [3,26,28-31]. The ER is a nonspecific binder that interacts with structurally diverse ligands, altering normal estrogen signaling through genomic and non-genomic pathways [31-34]. Xenoestrogens can act as agonists, partial agonists, or antagonists to ERs, altering normal gene expression levels and functions modulated by endogenous hormones [22]. The binding target of these xenoestrogens is the ligand binding domain (LBD) of the ERs. The LBD consists of twelve α-helices (H1-H12) and a beta-hairpin (Figure 1a). The H12 of LBD plays the key role of a molecular switch [35] through adopting distinct ligand-dependent conformations crucial for receptor activation [36] (Figure 1b and 1c respectively). When bound to an agonist, the LBD adopts an active conformation: H12 rests across H3 and H11, forming a groove to accommodate co-regulator binding and facilitate downstream activation process. When bound to an antagonist, H12 is displaced from this position resulting in the distortion of this co-regulator binding groove and the inhibition of receptor activation [37].

A battery of validated assays, both *in vivo *and high-throughput *in vitro*, have been developed to screen for mimics that act either as estrogens or anti-estrogens, but the cost of comprehensively testing hundreds of thousands of man-made chemicals would be formidable [38]. The timeline would also be highly protracted, given that in over a decade, barely the tip of the iceberg of the chemical universe, a few chemical classes, have been tested [38,39]. Finally, experimental techniques thus far validated are not comprehensive, as developmental endpoints, means to detect levels of no biological effect, and mixture and metabolism effects, among other limitations, are not adequately represented. Suffice it to say that a full EDC assessment across the universe of chemicals constitutes a daunting problem, and any *in silico *means to reduce costs and streamline the process would be a welcome prospect [28].

Computational techniques have often been used to complement experimental studies in order to assist with data analysis as well as improve results. In this instance, rapid *in silico *screening can be used not only to help identify and prioritize which class of compounds to screen, but also reduce the number of compounds to be tested. Docking is one of the popular techniques commonly used for a number of purposes, e.g. ligand pose prediction, ligand binding affinity prediction as well as identifying potential actives from a library of decoys in virtual screening (VS) [40]. In the past, docking studies performed on ERs have been carried out. A number of these studies developed models for the purpose of screening for potential ligands/EDCs based on either docking alone or in combination with three-dimensional (3D)-QSAR models: Zhang *et al*. [41] looked at both ERα and ERβ subtypes and successfully developed QSAR and docking models using large sets of ligands from various sources for the identification of potential EDCs; also looking at both ERα and ERβ subtypes, Wolohan *et al*.[42] built their model based on 3D-QSAR and docking using a diverse set of 36 estrogen ligands. While they demonstrated that the CoMFA models could correctly rank-order the ligands according to their relative binding affinities, and thus could be used for screening of novel subtype-selective ligands, incorporating results from docking failed to introduce further improvement to the existing predictions. Schapira *et al*. [43] docked over 5000 compounds across a range of nuclear receptors including the ERs and showed that VS performed on these receptors could be used to identify hits. Finally, Huang *et al*. [44] assembled a database called the Directory of Useful Decoys (DUD) using 2950 ligands across 40 targets (ERs included). Varying levels of enrichment were reported for the different targets studied, amongst which the results for ER had been found to be good with significant early enrichment. The above body of work shares a common outcome: the docking results demonstrate that models have utility to differentiate potential ligands (binders) from decoys (non-binders). While these methods have been shown to be useful, they however, (1) lack the ability to distinguish agonists from antagonists, and are thus unable to obviate or reduce experimental assays for further understanding of the mechanisms of actions; and (2) do not reflect the dynamic biological processes in the body whereby ERα and ligands interact with each other, and depending on the ligand type, leads to the adoption of distinct ERα conformations.

In view of this and as part of our continued research interest in EDCs (past works include [25,26,28-30,45-49]), we have developed an approach that can differentiate ligands in accordance with likelihood of activating or inhibiting or blocking the receptor (i.e. agonist or antagonist, respectively) and more closely mimics the dynamic nature of competing ligand-ERα complexes where agonists and antagonists impart different conformation changes not represented by a single rigid conformation found in prior docking models. Two separate docking models (SDMs) were employed, one based on an ERα agonist conformation crystal structure and the other based on ERα antagonist crystal structure. The competitive docking approach (CDA) uses both SDMs in that the agonist and antagonist SDMs compete in determining whether an individual ligand is assigned as an agonist or antagonist. The CDA takes into account and compares the non-covalent interactions between a specific ligand and the two separate docking models based on the respective docking scores of the docked complex and, therefore, better reflects the receptor-ligand interaction in reality whereby the more energetically favorable complex is favored. A ligand is assigned to be (in a winner take all strategy) the type, agonist or antagonist, corresponding to the most favorable docking score from the individual SDMs. We tested our models using two sets of ER ligands (one extracted from PDB crystal structures and another from the DUD [44]) and assessed the quality of our SDMs and CDA through virtual screening, using enrichment factors (EFs) as the performance metric. Results obtained showed that our CDA was able to differentiate agonists and antagonists with considerable accuracy and that the qualities of the CDA as well as its individual components (agonist and antagonist SDMs) are comparable to the work of others [44].

## Methods

### Study design

**Figure 2:**
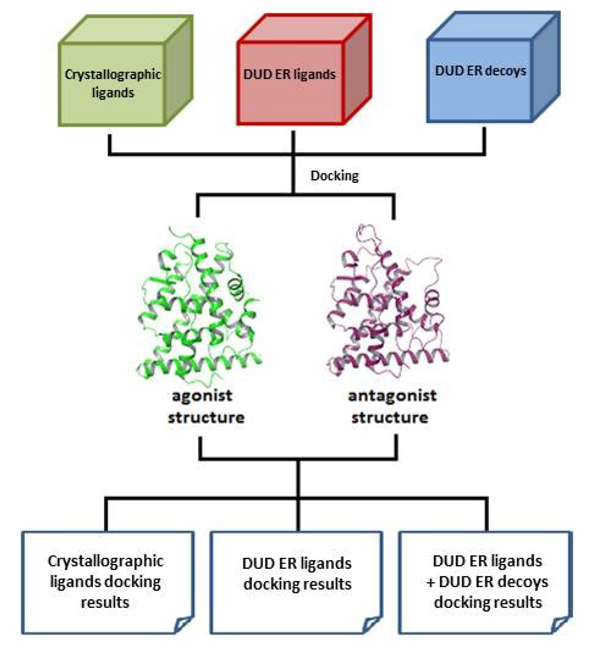
**Study design depicting the overall workflow of this study**. Three ligand sets are used for docking. While the first set of ligands is derived from the crystal structures available from the PDB, the second and third sets of ligands and decoys, respectively, are obtained from the DUD website. Results from the first and second sets of docking will be used to evaluate the ability of the CDA to differentiate agonists and antagonists while the results from the second and third sets of dockings will be combined and used to calculate enrichment factors.

Figure 2 depicts the overall study design and work flow. A preferred agonist ERα structure and a preferred antagonist ERα structure were selected from the PDB. Three sets of ligands comprising both agonists and antagonists as well as decoys were docked to the preferred ERα structures: the first set of ligands consisted of ligands extracted from ERα crystal complexes in the PDB; the second set were ER ligands obtained from the DUD website (http://dud.docking.org); and the third set consisted of ER agonist and antagonist inactive decoys, also obtained from the DUD website. A competitive approach was implemented in the docking procedures to yield the final results. The main purposes for carrying out these dockings were: firstly, to determine the ability of the CDA to differentiate agonists and antagonists using the first set of crystallographic ligands; secondly, to further validate the agonist-antagonist differentiating ability of the CDA using the second (larger) ligand set; and thirdly, to use VS and EF calculations to evaluate the quality and reliability of the CDA and its individual agonist and antagonist SDM components. Structural analyses were also performed on the ER crystal structures available in the PDB in order to assist in the rationalization of the docking results as well as to delineate structural differences between the ERα structures bound to different ligands.

### ERα structures for structural analysis

The ERα crystal structures available in the PDB were compiled for two main purposes: (1) to evaluate and make the most reasonable decision on the selection of two ERα structures (agonist and antagonist conformations) such that the chosen structures were the most representative structures; (2) for verification and rationalization of docking results.

Eighty four 3D structures of ERα ligand-binding domain complexes were downloaded from the PDB. Multimeric structures were reduced to monomeric and superimposed. Four structures, PDB IDs 2G5O, 3Q97, 1A52 [50], 2B23 [51], were excluded from the analysis. Structures 2G5O and 3Q97 were excluded because they were bound to ligands with unknown ligand type. Structure 1A52 was excluded because it was purported to contain an aberrant helix 12 conformation as a result of crystallization [50]. The 2B23 structure was excluded because it was an apo-protein with agonist-conformation-stabilized mutations [51].

### ERα structures for docking

The 3D structures of complexes of ERα bound with an agonist and an antagonist, i.e. estradiol (PDB ID: 1GWR) and 4-hydroxytamoxifen (PDB ID: 3ERT), respectively, were selected as the preferred docking target proteins. The preferred proteins were chosen based on three criteria: (1) highest possible resolution; (2) contained no mutations or modified residues; and (3) bound to an endogenous/well-studied ligand. While the first requirement ensured that protein structures used for docking were of a good quality, the second requirement was applied because some mutations have been found to have profound effects on the final conformation of a protein [35,37,51,52]. The third requirement was imposed such that the structures were a good representation of the proteins when bound to a typical ligand. Table 1 shows the details of the selected ERα structures for agonist and antagonist docking models.

**Table 1:** Selection of agonist and antagonist docking structures.

Structure	Resolution (Å)	Mutation/Modified residues	Bound ligand
1GWR (agonist)	2.4	-	Estradiol
3ERT (antagonist)	1.9	-	4-hydroxytamoxifen

Crystal structures selected from the PDB to be targets for the agonist and antagonist docking and the associated details.

### Ligand sets

The first set of ligands consisted of 66 compounds (47 agonists and 19 antagonists) that were extracted from the ERα complexes downloaded from the PDB (see Additional file 1 and Additional file 4). While the PDB contained 83 ligand-bound ERα structures, some were for the same ligand (e.g. estradiol and genistein) and were excluded, and two were bound to ligands of undetermined ligand type (PDB ID: 2G5O and 3Q97) and were also excluded. The second set of ligands consisted of 106 ER binders downloaded from the DUD, of which 67 were agonists and 39 antagonists (see Additional file 2). The third set of ligands which contained 4018 ER decoys (2570 agonist decoys and 1448 antagonist decoys), were also downloaded from the DUD website (see Additional file 3).

### Protein and ligand preparation

The preferred 3D ERα structures for docking agonists (1GWR) and antagonists (3ERT) were preprocessed before docking calculation using the *Protein Preparation Wizard *tool within the Maestro program by Schrodinger [53]. First, hydrogen atoms were added to the protein structures, bond orders were assigned and crystallographic waters were deleted. Then, the hydrogen bonds were optimized at pH 7 using the PROPKA program in Schrodinger before a restrained minimization was performed using the OPLS_2005 force field [54] whereby the convergence for the heavy atoms were set at RMSD 0.3 Å.

The crystallographic ligands, ER ligands and decoys downloaded from DUD were prepared using the *LigPrep *tool in Maestro. Possible ionization states were generated at pH 7.0 (+/- 2) using Epik [55,56], while the stereoisomers were determined from the 3D structures of the ligands.

### Grid generation and molecular docking

**Figure 3:**
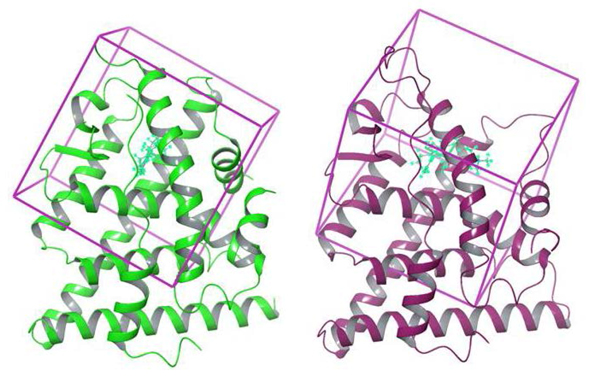
**Docking grid generation**. Docking grids were generated for the ERα agonist (green) and antagonist structures (purple) using Maestro. The boxes are centered at the cognate ligands i.e. estradiol and 4-hydroxytamoxifen respectively

Docking grids for both protein structures were generated using Maestro: the grid box was centered at the cognate ligands of the protein structures (estradiol and 4-hydroxytamoxifen respectively) while the maximum length of the dock ligands were set to 20 Å, as shown in Figure 3. Docking was performed with Glide using Standard Precision (SP) and the following parameters: ligand sampling was set to flexible, energy window for ring sampling set to 2.5 kcal/mol, number of poses per ligand at the initial phase of docking was set to 5000, number of poses per ligand kept for energy minimization was set to 400, and maximum number of minimization steps was set to 100. Post-docking minimization was allowed whereby the number of poses included per ligand was set to 5. Only one pose was written out per ligand in the final output. Docking with SP instead of Extra Precision (XP) [57] was used because the ultimate goal for this work was to use the developed model to screen large ligand libraries having up to hundreds of thousands of molecules. However, initial docking of diethyl-(1R,2S,3R,4S)-5,6-bis(4-hydroxyphenyl)-7-oxabicyclo[2.2.1]hept-7-ene-2,3-dicarboxylate (PDB ID: 2QH6 [51]) failed to produce any results; using XP in this case overcame the problem.

### Competitive docking approach agonist and antagonist determination

The CDA has five possible outcomes in determining ligand status, as shown in Table 2. If the ligand can be docked to neither the agonist nor the antagonist ERα structures, it is determined to be a non-binder. If it can be docked to only the agonist ERα structure or only to the antagonist ERα structure, it is determined to be an agonist or antagonist, respectively. If the ligand can be docked to both ERα structures, the determination corresponds to the ERα structure with the lowest docking score.

**Table 2:** Decision table used to determine ligand type based on the five possible outcomes of CDA.

	Outcomes		Ligand Type
	**Agonist SDM**	**Antagonist SDM**	

**Docking**	Negative	Negative	Non-binder
	Positive	Negative	Agonist
	Negative	Positive	Antagonist
	Positive	Positive	Agonist (dock score for agonist SDM < antagonist SDM)
	Positive	Positive	Antagonist (dock score for antagonist SDM < agonist SDM)

The first three rows of the table show straightforward ligand type determination. On the other hand, for a ligand that docks to both agonist and antagonist SDMs, its ligand-type is determined using the dock scores of the respective SDMs whereby the lower score is favored over the higher. A positive outcome indicates that a ligand can dock in the structure, vice versa for a negative outcome.

### Post-docking analyses

EF defined in equation (1) was used for estimating VS efficiency of the SDM and CDA:

(1) $$EF=\frac{TT_{scr}}{n_{scr}}\times \frac{N_{c}}{TT_{c}}$$

Where TT_scr _indicates the number of the true targets (i.e. agonists/antagonists) among the number of chemicals screened n_scr _(i.e. agonists/antagonists and decoys) at a given percentage of the entire dataset. N_c _and TT_c _denote the total number of chemicals and the total number of true targets in the VS experiment, respectively. EF values were calculated at different percentages of the total chemicals to measure VS performance for screening agonists and antagonists using the SDM and the CDA separately. This was followed by VS efficiency comparative analyses.

The backbone RMSD and all-atom RMSD of the ERα structures were calculated using equation (2) in a Matlab script:

(2) $$RMSD=\sqrt{\frac{1}{n}\overset{n}{\underset{i=1}{\sum }}\left({\left(V_{ix}-W_{ix}\right)}^{2}+{\left(V_{iy}-W_{iy}\right)}^{2}+{\left(V_{iz}-W_{iz}\right)}^{2}\right)}$$

Where n denotes the number of atoms used in the calculation and x, y and z denote the Cartesian coordinates of atom i in the two ERα structures, V and W, being compared.

The graphics of ERα structures in this paper were generated using Maestro.

## Results and discussion

### Docking results of crystallographic ligands

Table 3 gives predictions by SDMs alone versus truth for the crystallography ligands. Of 47 true agonists, 43 docked to both the agonist and antagonist SDMs, such that no type determination can be made. This indicates that majority (91.5%) of the agonists could not be differentiated from the antagonists despite successfully docked in the ERα conformation for agonists. The remaining four agonists docked to only the antagonist SDM and were thus falsely typed. Of the 19 true antagonists, 17 docked to only the antagonist SDM, and were correctly typed, while the remaining two docked to both SDMs such that no type determination is possible. This indicates that most (89.5%) of the antagonists were differentiated from the agonists.

**Table 3:** SDMs predictions of crystallographic ligand set

		Ligand type (truth)		Total (Predicted)
		**Agonist**	**Antagonist**	

Ligand type (Predicted)	Not determinable (docks to both agonist and antagonist SDMs)	43	2	45
	Non-binder (docks neither agonist nor antagonist SDM)	0	0	0
	Agonist (docks agonist SDM only)	0	0	0
	Antagonist (docks antagonist SDM only)	4	17	21
Total (truth)		47	19	

The table shows the predictions made by the SDMs for the crystallographic ligand set versus truth. The columns represent the truth (agonist and antagonist) while the rows represent the prediction outcomes (not determinable, non-binder, agonist and antagonist).

Table 4 gives predictions by the CDA versus truth for the crystallography ligands. CDA correctly predicted 35 of 47 true agonists, and falsely predicted 12 as antagonists. The successful rate for agonist prediction was increased to 74.5% compared to 0% (0 of 47) of SDMs. For antagonists, 18 of 19 were correctly predicted, showing a slight improvement compared to antagonist SDM (94.7% of CDA vs 89.5% of antagonist SDM). Thus, CDA correctly predicted type for 80.3% (53 of 66) ligands, compared to only 25.8% (17 of 66) correct predictions using the SDMs separately. The difference, of course, is solely due to choosing ligand type based on lowest docking score for ligands that docked to both SDMs.

**Table 4:** CDA predictions of crystallographic ligand set

		Ligand type (truth)		Total (Predicted)
		**Agonist**	**Antagonist**	

Ligand type (Predicted)	Not determinable (docks to both agonist and antagonist SDMs)	-	-	-
	Non-binder (docks neither agonist nor antagonist SDM)	0	0	0
	Agonist (docks agonist SDM only **OR **dock score for agonist SDM < antagonist SDM)	35	1	36
	Antagonist (docks antagonist SDM only **OR **dock score for antagonist SDM < agonist SDM)	12	18	30
Total (truth)		47	19	

The table shows the predictions made by the CDA for the crystallographic ligand set versus truth. The columns represent the truth (agonist and antagonist) while the rows represent the prediction outcomes (non-binder, agonist and antagonist).

**Figure 4:**
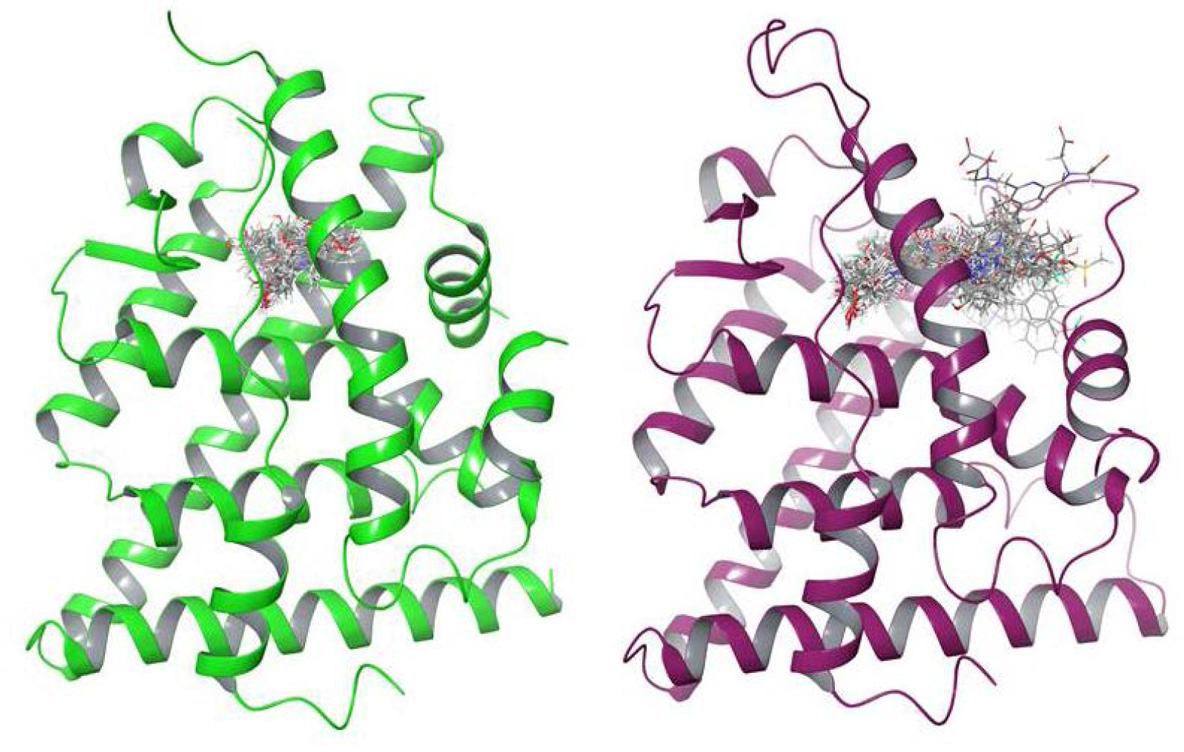
**Docked ligands in the agonist and antagonist structures**. The docked crystallographic ligands in the agonist (green) and antagonist (purple) structures: These diagrams clearly show that ligands which are sufficiently small in size are able to fit within both agonist and antagonist structures while larger ligands only fit into the antagonist structure.

The primary difference between ERα agonist and antagonist molecules is molecular size, with agonists generally found to be the smaller. ERα agonists and antagonists alike have steroidal cores, but most antagonists compared to agonists have bulky pendant side chains of varying lengths attached to this steroid core, significantly increasing molecule size [36,58]. It is precisely this difference that causes the difference in prediction accuracy between the agonists and antagonists. The agonists (and some smaller antagonists) are able to fit within both agonist and antagonist ERα binding pockets, as depicted in Figure 4, therefore leading to the likelihood of these ligands being predicted as either an agonist or antagonist by the CDA. Conversely, a significant number of antagonists are too large to be accommodated by the agonist ERα binding pocket and only bind to the antagonist ERα. This reason directly results in the higher prediction accuracy for antagonists compared to the agonists.

The difference in the prediction accuracy can also be seen as a product of rigid protein docking. Docking a flexible ligand to a rigid receptor, as in this study, is a common practice. However, fixing protein conformation has long been seen as a limitation of docking as proteins are conformationally dynamic in reality [59,60]. Unfortunately, allowing full protein flexibility is extremely computationally expensive and remains impractical with the current state-of-the-art [59]. Partially flexible docking i.e. allowing side chain flexibility of a few key residues in the binding pocket [59-61] is a reasonable trade-off between computational time and accuracy and can be used for improving this docking study.

**Figure 5:**
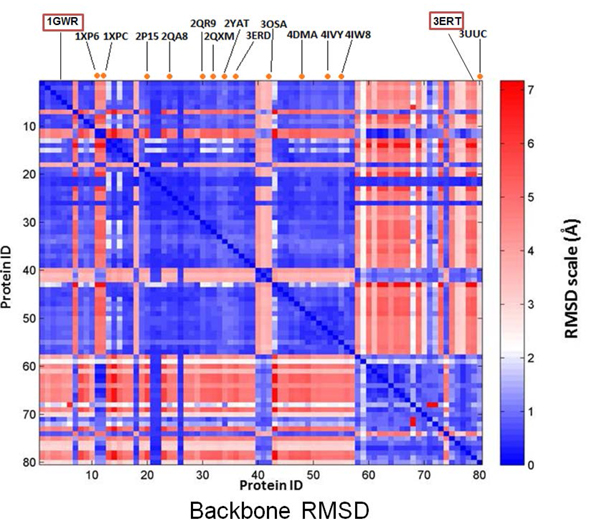
**Backbone analysis of ERα crystal structures**. Structural analysis of the ERα crystal structures in the PDB was performed using RMSD. Protein IDs 1-57 represent the agonist-bound conformations while 58-80 represent antagonist-bound structure according to the literature. A number of structures in both agonist-bound and antagonist-bound categories have been found to deviate from the norm, displaying characteristics which more resemble those of the other category. The orange circles situated at the top of the figure denote the incorrectly predicted ligands with their associated PDB ID. The two chosen protein conformations i.e. agonist structure (PDB ID: 1GWR) and antagonist structure (PDB ID: 3ERT), with a RMSD of 4.687 between each other, are also shown.

Despite the significant improvement observed in the CDA, 13 molecules (12 agonists and 1 antagonist) were incorrectly predicted. A collective ERα backbone structural analysis of the 80 ERα crystal structures (Figure 5) revealed some interesting observations. Three compounds, (i) (2S,3R)-2-(4-2-[(3S,4S)-3,4-dimethylpyrrolidin-1-yl]ethoxyphenyl)-3-(4-hydroxyphenyl)-2,3dihydro-1,4-benzoxathiin-6-ol, (ii) (2S,3R)-3-(4-hydroxyphenyl)-2-(4-{[(2R)-2-pyrrolidin-1-ylpropyl]oxy}phenyl)-2,3-dihydro-1,4-benzoxathiin-6-ol, and (iii) 4-[1-(3-methylbut-2-en-1-yl)-7-(trifluoromethyl)- 1H-indazol-3-yl]benzene-1,3-diol (PDB ID: 1XP6, 1XPC, 3OSA respectively), despite being reported as partial-agonists [37,62], were predicted to be antagonists by our CDA. A closer look at the backbone analysis revealed that these three compounds were bound to ERα structures that more closely resembled the antagonist-bound conformations. A number of possible scenarios could potentially explain this contradictory observations: (1) the partial nature of these ligands (e.g. partial agonism/antagonism) leads to the destabilization of the protein structure instead of the adoption of a complete agonist or antagonist conformation; (2) the final resultant conformation of the proteins is dictated more by the presence of agonist- and antagonist-conformation inducing mutations in these protein structures than by the type of the bound ligand; and (3) the mis-assignment of these ligand types. Scenario (1) may be applied to the first two compounds, bound to 1XP6 and 1XPC. These compounds are partial agonists arising from the modifications of the parent compound dihydrobenzoxathiins, which is a selective ERα modulator demonstrating antagonistic actions. The partial agonistic characteristics introduced by the modifications had resulted in the destabilization of the antagonist conformation of the proteins particularly at the helix 12 position [62] but did not cause the proteins to switch from an antagonist conformation to an agonist conformation. This is in line with the observations reported by Pike *et al*.[63] in which a partial agonist showed lower efficacy when compared to a full agonist. In addition to the first scenario, the second scenario may also be applicable to the third compound, a partial agonist bound to an ERα structure containing L536S and L372R mutations. These mutations have been reported to stabilize ERα at antagonist conformations [37]. Two other incorrect predictions involving (17beta)-17-(E)2-[2-(trifluoromethyl)phenyl]vinyl) estra-1(10),2,4-triene-3,17-diol and estradiol-pyridinium tetraacetic acid (PDB ID: 2P15 [35] and 2YAT [64]), can be rationalized by the large molecular size of these compounds that cannot be accommodated by the agonist ER conformation. When bound to the 2P15 and 2YAT complexes, the induced fit that occurred allowed these rather large agonists to fit into their respective protein structures [35,64]. The remaining agonists i.e. genistein, dimethyl(1R,4S)-5,6-bis(4-hydroxyphenyl)-7-oxabicyclo[2.2.1]hepta-2,5-diene-2,3-dicarboxylate, 2-amino-1-methyl-6-phenylimidazole[4,5-B]pyrine, diethylstilbestrol, 2'-bromo-6'-(furan-3-yl)-4'-(hydroxymethyl) biphenyl-4-ol, 4-[1-(but-3-en-1-yl)-7-(trifluoromethyl)-1H-indazol-3-yl]benzene-1,3-diol and 4-[1-(3-methylbut-2-en-1-yl)-7-(trifluoromethyl)-1H-indazol-3-yl]benzene-1,3-diol (PDB ID: 2QA8 [51], 2QR9 [51], 2QXM [51], 3ERD [65], 4DMA [66], 4IVY [67] and 4IW8 [67]) that were predicted as antagonists docked to both agonist and antagonist ER structures, but scored better as antagonists due to more favorable interactions. The reverse apply to the antagonist 4,4'-(2,2-dichloroethene-1,1-diyl)diphenol (PDB ID: 3UUC [68]) that was predicted as an agonist.

**Figure 6:**
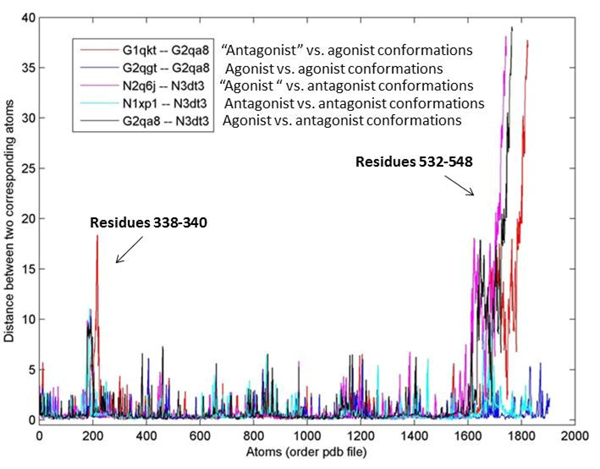
**All-atom analysis of the ERα crystal structures**. The graph shows the all-atom RMSD for five pairs of ERα complexes found to be interesting in the study. Note: "agonist" with parentheses represents structure which was bound to an antagonist according to the literature, but demonstrated an agonist conformation, and vice versa for the "antagonist" structure. Letters G and N in front of the PDB IDs denote the types of ligand bound to the structures, as reported in the literature. Major differences were found between the antagonist's and antagonist's conformations whereby these differences were found to lie in the region between residues 338-340 (loop linking helix 2 and helix 3) and 532-548 (end of helix 11 to end of helix 12). See Figure 7 for diagrams showing these differences.

**Figure 7:**
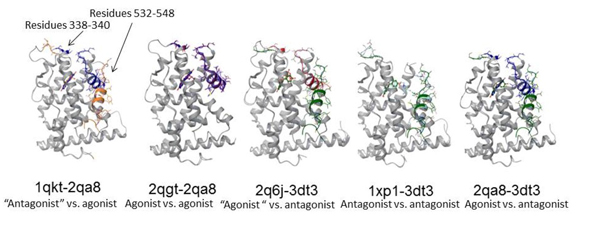
**Differences between the agonist's and antagonist's conformations**. The differences of residues 338-340 (loop linking helix 2 and helix 3) and 532-548 (end of helix 11 to end of helix 12) are shown in the five pairs of protein conformations as mentioned in Figure 6. Color codes: 1QKT (orange), 2QA8 (blue), 2QGT (pink), 2Q6J (orange red), 3DT3 (green) and 1XP1 (light green). The cognate ligands of these structures were also shown using the same color codes.

The structural differences between the agonist's and antagonist's conformations were studied in finer detail using five pairs of ERα structures (Figure 6) which were found to be interesting ("agonist" with parentheses represents structure which was bound to an antagonist as reported by the literature, but demonstrated an agonist conformation, and vice versa for the "antagonist" structure). From the analysis of the all-atom RMSD, we observed that the major differences between the agonist's and antagonist's conformations lie in the loop regions that connect helix 2 and helix 3 (residues 338-340) of the ERα ligand binding domain, as well as, in the stretch of residues that begin from the end of helix 11 to the end of helix 12 (residues 532-548) (Figure 7). This is due to the fact that in the agonist conformation, helix 12 is positioned against helix 11 and helix 3, therefore limiting the mobility of helix 11 and helix 3 as compared to the antagonist conformation [37].

### Docking results of DUD ERα ligands

Table 5 gives predictions by agonist and antagonist SDMs versus truth for ligands from the DUD database containing ER binders for benchmarking. The overall results are highly reminiscent of those obtained in the crystallographic ligand set. No agonists could be differentiated from antagonist. Of 67 true agonists, 66 docked to both the agonist and antagonist ERα structures, such that no type determination could be made. The remaining agonist docked to only the antagonist ERα structure, and was thus falsely typed. A better outcome was again observed for the antagonists. Of the 39 true antagonists, 34 docked to only the antagonist ERα structure, and were correctly typed, and two were unable to dock to any of the two ERα structures, thus were predicted as non-binders, while the remaining three docked to both ERα structures such that no type determination was possible.

**Table 5:** SDMs predictions of DUD ER ligand set

		Ligand type (truth)		Total (Predicted)
		**Agonist**	**Antagonist**	

Ligand type (Predicted)	Not determinable (docks to both agonist and antagonist SDMs)	66	3	69
	Non-binder (docks neither agonist nor antagonist SDM)	0	2	2
	Agonist (docks agonist SDM only)	0	0	0
	Antagonist (docks antagonist SDM only)	1	34	35
Total (truth)		67	39	

The table shows the predictions made by the SDMs for the DUD ER ligand set versus truth. The columns represent the truth (agonist and antagonist) while the rows represent the prediction outcomes (not-determinable, non-binder, agonist and antagonist).

Table 6 gives predictions by CDA versus truth for the DUD ligands. The CDA again was superior in agonist prediction than the SDMs. CDA correctly predicted 70.1% (47 of 67) agonists and 92.3% (36 of 39) antagonists, as compared to SDMs: 0% and 87.2% for agonists and antagonists respectively. The overall accuracy of CDA for differentiating between agonists and antagonists was improved to 78.3%, from 32.1% of the SDMs. The improvement in typing agonists versus antagonists is similar for the DUD ligands as for the crystallographic ligands, with the majority of improvement occurring for the agonists.

**Table 6:** CDA predictions of DUD ER ligand set.

		Ligand type (truth)		Total (Predicted)
		**Agonist**	**Antagonist**	

Ligand type (Predicted)	Not determinable (docks to both agonist and antagonist SDMs)	-	-	-
	Non-binder (docks neither agonist nor antagonist SDM)	0	2	2
	Agonist (docks agonist SDM only **OR **dock score for agonist SDM < antagonist SDM)	47	1	48
	Antagonist (docks antagonist SDM only **OR **dock score for antagonist SDM < agonist SDM)	20	36	56
Total (truth)		67	39	

The table shows the predictions made by the CDA for the DUD ER ligand set versus truth. The columns represent the truth (agonist and antagonist) while the rows represent the prediction outcomes (non-binder, agonist and antagonist).

**Figure 8:**
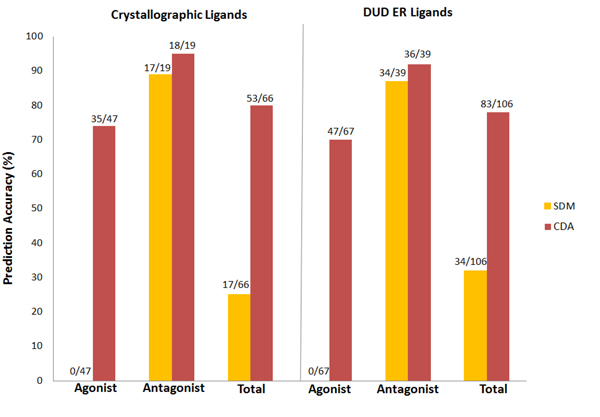
**Prediction accuracy of the SDMs and CDA**. The bar charts show the prediction accuracy of the SDMs (yellow) and CDA (red) for the crystallographic and DUD ER ligand sets. The bar heights denote the total number of ligands in each category. In all cases, CDA outperformed the SDMs, particularly in the case of agonist predictions.

Figure 8 compares the prediction performance of SDMs and CDA for both the crystallographic and DUD ligands. Clearly, the CDA (in red) performed consistently and significantly better than SDMs (in yellow), in all cases, highlighting the predictive accuracy improvement using CDA. While both SDMs and CDA performed comparably well in antagonist prediction, most improvement was in agonist prediction.

Using 199 molecular descriptors, Li *et al *[69] developed support vector machine, *k*-NN, probabilistic neural network, and C4.5 decision tree structure-activity relationship (SAR) models for predicting ER agonists based on a data set of 243 agonists and 463 non-agonists. One 5-fold cross validation was used to estimate the performance of their models: 66.3-83.8% agonist prediction accuracy and 83.8-91.1% non-agonist prediction accuracy. As a comparison, our CDA had 74.5% and 70.1% agonist prediction accuracy and 94.7% and 92.3% antagonist prediction accuracy for the crystallographic and DUD ER ligands, respectively. Though our results were similar to those from Li *et al*. [69], we should point out that the comparison is not a head-to-head comparison. First, majority of the non-agonists used by Li *et al*. are ER non-binders instead of antagonists. Therefore, more precisely, Li *et al*. models differentiate between ER agonists and ER non-binders - this, in comparison, is easier than differentiating the biological functions of ER binders (between agonist or antagonist), which is our objective. Second, the performance of the SAR models was estimated by only one run of 5-fold cross validation and, thus, the validation results are not robust: different division of the data set into five folds most likely have different performance. In contrast to this, our method is protein structure based and, thus, ligand set independent.

### Virtual screening results

**Figure 9:**
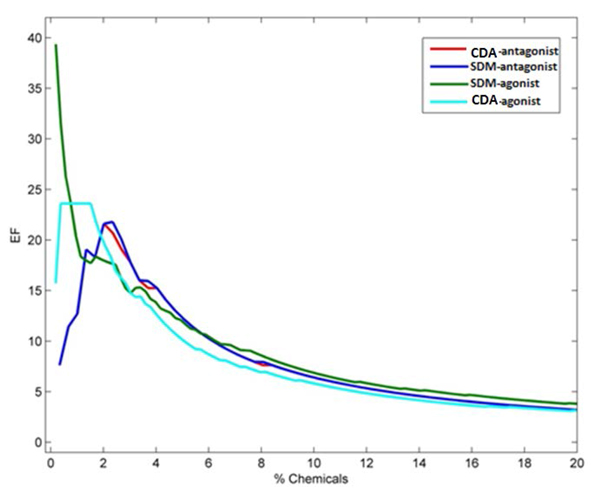
**Performance of SDMs and CDA in virtual screening**. The lines show the enrichment factors calculated for the SDMs and CDA in the agonists (green and cyan respectively) and antagonists (blue and red respectively) VSs. Larger differences are observed between the SDMs and CDA for the agonist VS compared to antagonist VS, which show very little difference between the models.

The VS calculation was done for the agonist SDM after combining 67 true agonists and 2570 decoys from DUD. The calculation was repeated for the antagonist SDM after combining 39 true antagonists and 1448 decoys from DUD. Next, the antagonist SDM result was obtained for the 67 agonists and 2570 decoys, and the agonist SDM results obtained for the 39 antagonists and 1448 decoys. Finally, the agonist SDM and antagonist SDM results for each dataset were combined with the CDA. The VS performances were analyzed using EFs plotted in Figure 9. The agonist and antagonist SDMs had peak enrichments of about 40 and 22, respectively. A high EF of about 40 was obtained for the agonist SDM in the early stage of the screening, with a steep subsequent decrease with increasing ligands screened, indicating that most of the agonists were detected at a very early stage of screening (less than 1%). Agonist screening with the CDA, on the other hand, produced a peak EF of 24 at 2% chemicals, indicating that more agonists were screened out compared to agonist SDM. The enrichment for the antagonist SDM and CDA were generally similar in shape and magnitude, and both less than for agonists, in agreement with the results reported by Huang *et al*. [44] but in contrast to docking results (Tables 3, 4, 5, 6) that showed higher accuracy for antagonists.

In order to evaluate the quality of the individual docking models used in the CDA, a comparison of enrichment for our SDMs and those reported by Huang *et al*. (DUD database) [44] was made and the results summarized in Table 7. Results were comparable at 1% and 20% of chemicals screened. EF_max _of our agonist SDM was higher than Huang *et al*., i.e. 39.4 vs. 29.6. However, for antagonist screening, Huang *et al*. reported a much higher EF_max _of 101.6, compared to our 21.8. The calculation to obtain the remarkably high EF_max _value of 101.6 was impossible according to equation (1) and was not demonstrated in the published article, therefore warranting further verification.

**Table 7:** Comparison of enrichment factors of SDMs with literature.

	Agonist		Antagonist	
	**SDM**	**Huang *et al***.	**SDM**	**Huang *et al*.**

1%	20.5	19.2	12.7	12.7
2%	17.9	-	21.6	-
3%	14.8	-	17.8	-
4%	13.9	-	15.3	-
5%	12.1	-	12.2	-
20%	3.8	4.5	3.2	1.3
Max	39.4	29.6	21.8	101.6*

Comparisons of the calculated EFs at different % of database screened and EF_max _for the SDMs reported in our work and Huang *et al*.[44]. *The remarkably high EF_max _reported by Huang *et al*. for antagonist may require further verification.

Differences between the EFs of SDMs and CDA shown in Figure 9 occur in early stages of < 3% of chemicals screened. Table 8 shows that in the 1% to 3% interval, CDA performed better than SDM. Although the differences were modest (one should bear in mind the promiscuity of ERs when it comes to ligand binding), the result adequately demonstrated the potential usefulness of CDA in VS.

**Table 8:** Comparison of enrichment factors of SDMs and CDA.

	Agonist		Antagonist	
	**SDM**	**CDA**	**SDM**	**CDA**

1%	20.5	23.6	12.7	12.7
2%	17.9	19.3	21.6	21.6
3%	14.8	14.8	17.8	17.8
4%	13.9	12.7	15.3	15.3
5%	12.1	10.3	12.2	12.2
20%	3.8	3.1	3.2	3.2
Max	39.4	23.6	21.8	21.6

Comparisons of the calculated EFs at different % of database screened and EF_max _for the SDMs and CDA. For agonist screening, CDA performs better at the early stage (1-3%) compared to SDM. For antagonist screening, comparable results are obtained. The EF_max _for the SDMs are better than the CDA, however, these peaks occur at a later stage of screening.

## Conclusions

We have developed a competitive docking approach for performing ligand-docking in ERs. The quality of the individual components (SDMs) on which the CDA depends was evaluated and found comparable to other published models [44]. The CDA was demonstrated to provide discriminatory power to segregate agonists and antagonists at useful accuracy. It was also shown to provide comparable enrichment to the results of Huang *et al*. [44] in a large data set comprising true and decoy ligands. The CDA could be useful as part of an EDC screening program to identify and rank potential binders to aid setting of testing priority. The ability to distinguish agonists from antagonists could be further useful since some compounds could be tested in either an agonist or antagonist assay, but not both, reducing cost. The CDA approach is extensible to other receptor targets both to screen for potential binders and to differentiate between agonists and antagonists, and is as applicable in drug discovery as for regulatory testing purposes.

## Abbreviations

3D: three-dimensional; CDA: competitive docking approach; EADB: Estrogenic Activity Database; EDCs: Endocrine disrupting chemicals; EDKB: Endocrine Disruptor Knowledge Base; EDSP: Endocrine Disruptor Screening Program; EF: enrichment factor; EPA: Environmental Protection Agency; ER: estrogen receptor; FDA Food and Drug Administration; PDB: protein data bank; SAR: structure-activity relationship; SDM: separate docking model; SP: standard precision; VS: virtual screening; XP: extra precision.

## Competing interests

The authors declare that they have no competing interests.

## Authors' contributions

HN performed all calculations and data analysis, and wrote the first draft of manuscript. WZ, HL, MS, and WG contributed to the data analysis, verified the calculations. RP, WT and HH wrote the final manuscript. HH developed the original idea and guided the data analysis and presentation of results. All authors read and approved the final manuscript.

## Supplementary Material

Additional file 1 Crystallographic ligands Click here for file

Additional file 2 DUD ER ligands Click here for file

Additional file 3 DUD ER decoys Click here for file

Additional file 4 Chemical structures of crystallographic ligands Click here for file

## Declarations

Publication costs of this article were funded by the US government.

This article has been published as part of *BMC Bioinformatics *Volume 15 Supplement 11, 2014: Proceedings of the 11th Annual MCBIOS Conference. The full contents of the supplement are available online at http://www.biomedcentral.com/bmcbioinformatics/supplements/15/S11.
