# Supplementary material for: Competitive molecular docking approach for predicting estrogen receptor subtype α agonists and antagonists
Source: BMC Bioinformatics. 2014 Oct 21;15(Suppl 11):S4. doi: 10.1186/1471-2105-15-S11-S4 (PMC4251048; doi:10.1186/1471-2105-15-S11-S4)
Supplement: Additional file 1 — Crystallographic ligands [file 1471-2105-15-S11-S4-S1.pdf]

Additional file 1 The crystallographic ligands used in the first set of docking as described in the study design. Information about the PDB ID, ligand type and docking scores in the agonist and antagonist structures are provided. The chemical structures of the ligands can be found in Figure S1, labelled according to the ID listed in the first column.

ID	PDB	Ligand	Type	Docking Score (ago structure)	Docking Score (ant structure)
**X1**	3L03	(14beta,15alpha,16alpha,17alpha)-estra-1,3,5(10)-triene-3,15,16,17-tetrol	ago	-10.807	-5.35
**X2**	2P15	(17BETA)-17-{(E)-2-[2-(TRIFLUOROMETHYL)PHENYL]VINYL}ESTRA-1(10),2,4-TRIENE-3,17-DIOL	ago	-	-4.117
**X3**	1XP6	(2S,3R)-2-(4-{2-[(3S,4S)-3,4-DIMETHYLPYRROLIDIN-1-YL]ETHOXY}PHENYL)-3-(4-HYDROXYPHENYL)-2,3-DIHYDRO-1,4-BENZOXATHIIN-6-OL	ago	-	-7.662
**X4**	1XPC	(2S,3R)-3-(4-HYDROXYPHENYL)-2-(4-{[(2R)-2-PYRROLIDIN-1-YLPROPYL]OXY}PHENYL)-2,3-DIHYDRO-1,4-BENZOXATHIIN-6-OL	ago	-	-11.953
**X5**	2Q70	(3AS,4R,9BR)-2,2-DIFLUORO-4-(4-HYDROXYPHENYL)-1,2,3,3A,4,9B-HEXAHYDROCYCLOPENTA[C]CHROMEN-8-OL	ago	-10.452	-4.845
**X6**	2I0J	(3AS,4R,9BR)-4-(4-HYDROXYPHENYL)-1,2,3,3A,4,9B-HEXAHYDROCYCLOPENTA[C]CHROMEN-8-OL	ago	-10.131	-8.965
**X7**	2POG	(3AS,4R,9BR)-4-(4-HYDROXYPHENYL)-1,2,3,3A,4,9B-HEXAHYDROCYCLOPENTA[C]CHROMEN-9-OL	ago	-9.728	-7.58
**X8**	2QE4	(3AS,4R,9BR)-4-(4-HYDROXYPHENYL)-6-(METHOXYMETHYL)-1,2,3,3A,4,9B-HEXAHYDROCYCLOPENTA[C]CHROMEN-8-OL	ago	-10.384	-8.36
**X9**	2QGT	(9BETA,11ALPHA,13ALPHA,14BETA,17ALPHA)-11-(METHOXYMETHYL)ESTRA-1(10),2,4-TRIENE-3,17-DIOL	ago	-10.356	-8.728
**X10**	3HM1	(9beta,13alpha)-3-hydroxyestra-1,3,5(10)- trien-17-one	ago	-9.875	-8.196

**X11**	3HLV	(9beta,13alpha,16beta)-3,16-dihydroxyestra-1,3,5(10)-trien-17-one	ago	-10.351	-8.686
**X12**	1L2I	(R,R)-5,11-CIS-DIETHYL-5,6,11,12-TETRAHYDROCHRYSENE- 2,8-DIOL	ago	-10.73	-9.063
**X13**	1X7E	[5-HYDROXY-2-(4-HYDROXYPHENYL)-1-BENZOFURAN-7-YL]ACETONITRILE	ago	-9.319	-8.054
**X14**	2B1Z	17-METHYL-17-ALPHA-DIHYDROEQUILENIN	ago	-9.791	-5.104
**X15**	2QXM	2-AMINO-1-METHYL-6-PHENYLIMIDAZO[4,5-B]PYRID	ago	-7.873	-8.21
**X16**	4DMA	2'-bromo-6'-(furan-3-yl)-4'-(hydroxymethyl)biphenyl-4-ol	ago	-9.085	-9.234
**X17**	4IWF	2-chloro-3'-fluoro-3-[(E)-(hydroxyimino)methyl]biphenyl- 4,4'-diol	ago	-8.785	-8.28
**X18**	2QGW	3-CHLORO-2-(4-HYDROXYPHENYL)-2H-INDAZOL-5- OL	ago	-8.749	-8.643
**X19**	2QAB	3-ETHYL-2-(4-HYDROXYPHENYL)-2H-INDAZOL-5- OL	ago	-9.315	-8.781
**X20**	2QSE	4-(2-amino-1-methyl-1H-imidazo[4,5-b]pyridin- 6-yl)phenol	ago	-8.522	-8.292
**X21**	2QA6	4-(6-HYDROXY-1H-INDAZOL-3-YL)BENZENE-1,3-DIOL	ago	-8.636	-7.906
**X22**	3UUA	4,4'-(1,1,1,3,3,3-hexafluoropropane-2,2-diyl)diphenol	ago	-8.879	-8.265
**X23**	3UU7	4,4'-PROPANE-2,2-DIYLDIPHENOL	ago	-8.741	-8.339
**X24**	4IWC	4,4'-thiene-2,5-diylbis(3-methylphenol)	ago	-7.149	-5.957
**X25**	2G44	4-[(1S,2R,5S)-4,4,8-TRIMETHYL-3-OXABICYCLO[3.3.1]NON-7-EN-2-YL]PHENOL	ago	-7.934	-7.773
**X26**	1ZKY	4-[(1S,2S,5S)-5-(HYDROXYMETHYL)-6,8,9-TRIMETHYL-3-OXABICYCLO[3.3.1]NON-7-EN-2-YL]PHENOL	ago	-8.613	-7.578
**X27**	2B1V	4-[(1S,2S,5S)-5-(HYDROXYMETHYL)-8-METHYL-3-OXABICYCLO[3.3.1]NON-7-EN-2-YL]PHENOL	ago	-8.741	-7.985
**X28**	2FAI	4-[(1S,2S,5S,9R)-5-(HYDROXYMETHYL)-8,9-DIMETHYL-3-OXABICYCLO[3.3.1]NON-7-EN-2-YL]PHENOL	ago	-9.121	-7.911

**X29**	4IV2	4-[1-(2-methylpropyl)-7-(trifluoromethyl)- 1H-indazol-3-yl]benzene-1,3-diol	ago	-9.548	-9.408
**X30**	3OSA	4-[1-(3-methylbut-2-en-1-yl)-7-(trifluoromethyl)- 1H-indazol-3-yl]benzene-1,3-diol	ago	-9.462	-10.351
**X31**	4IW8	4-[1-(3-methylbut-2-en-1-yl)-7-(trifluoromethyl)- 1H-indazol-3-yl]benzene-1,3-diol	ago	-9.44	-10.322
**X32**	4IVY	4-[1-(but-3-en-1-yl)-7-(trifluoromethyl)- 1H-indazol-3-yl]benzene-1,3-diol	ago	-7.362	-10.065
**X33**	3OS9	4-[1-allyl-7-(trifluoromethyl)-1H-indazol- 3-yl]benzene-1,3-diol	ago	-9.095	-8.694
**X34**	3OS8	4-[1-benzyl-7-(trifluoromethyl)-1H-indazol- 3-yl]benzene-1,3-diol	ago	-10.177	-7.760
**X35**	4IUI	4-[1-butyl-7-(trifluoromethyl)-1H-indazol- 3-yl]benzene-1,3-diol	ago	-9.274	-8.813
**X36**	4IV4	4-[2-(2-methylpropyl)-7-(trifluoromethyl)- 2H-indazol-3-yl]benzene-1,3-diol	ago	-10.114	-9.439
**X37**	4IW6	4-[2-(but-3-en-1-yl)-7-(trifluoromethyl)- 2H-indazol-3-yl]benzene-1,3-diol	ago	-10.004	-9.557
**X38**	4IVW	4-[2-benzyl-7-(trifluoromethyl)-2H-indazol- 3-yl]benzene-1,3-diol	ago	-9.257	-5.391
**X39**	4IU7	4-[2-ethyl-7-(trifluoromethyl)-2H-indazol- 3-yl]benzene-1,3-diol	ago	-9.707	-9.64
**X40**	2QH6	DIETHYL (1R,2S,3R,4S)-5,6-BIS(4-HYDROXYPHENYL)-7-OXABICYCLO[2.2.1]HEPT-5-ENE-2,3-DICARBOXYLATE	ago	-7.837	-4.858
**X41**	3ERD	DIETHYLSTILBESTROL	ago	-8.979	-9.978
**X42**	2QR9	DIMETHYL (1R,4S)-5,6-BIS(4-HYDROXYPHENYL)-7-OXABICYCLO[2.2.1]HEPTA-2,5-DIENE-2,3-DICARBOXYLATE	ago	-8.516	-9.143
**X43**	1G50	ESTRADIOL	ago	-10.488	-8.332
**X44**	2YAT	ESTRADIOL-PYRIDINIUM TETRAACETIC ACID	ago		-5.08

**X45**	3Q95	ESTRIOL	ago	-10.386	-8.638
**X46**	2QA8	GENISTEIN	ago	-8.912	-9.477
**X47**	1GWQ	RALOXIFENE CORE	ago	-9.148	-9.139
**X48**	1XQC	(1S)-1-{4-[(9AR)-OCTAHYDRO-2H-PYRIDO[1,2-A]PYRAZIN-2-YL]PHENYL}-2-PHENYL-1,2,3,4-TETRAHYDROISOQUINOLIN-6-OL	ant	-	-11.44045
**X49**	1R5K	(2E)-3-{4-[(1E)-1,2-DIPHENYLBUT-1-ENYL]PHENYL}ACRYLIC ACID	ant	-	-4.34
**X50**	1YIM	(2R,3R,4S)-3-(4-HYDROXYPHENYL)-4-METHYL-2-[4-(2-PYRROLIDIN-1-YLETHOXY)PHENYL]CHROMAN-6-OL	ant	-	-11.401
**X51**	1YIN	(2R,3R,4S)-5-FLUORO-3-(4-HYDROXYPHENYL)-4-METHYL-2-[4-(2-PIPERIDIN-1-YLETHOXY)PHENYL]CHROMAN-6-OL	ant	-	-11.857
**X52**	1SJ0	(2S,3R)-2-(4-(2-(PIPERIDIN-1-YL)ETHOXY)PHENYL)-2,3-DIHYDRO-3-(4-HYDROXYPHENYL)BENZO[B][1,4]OXATHIIN-6-OL	ant	-	-11.773
**X53**	1XP1	(2S,3R)-2-(4-{2-[(3R,4R)-3,4-DIMETHYLPYRROLIDIN-1-YL]ETHOXY}PHENYL)-3-(4-HYDROXYPHENYL)-2,3-DIHYDRO-1,4-BENZOXATHIIN-6-OL	ant	-	-7.306
**X54**	1XP9	(2S,3R)-3-(4-HYDROXYPHENYL)-2-(4-{[(2S)-2-PYRROLIDIN-1-YLPROPYL]OXY}PHENYL)-2,3-DIHYDRO-1,4-BENZOXATHIIN-6-OL	ant	-	-11.848
**X55**	2OUZ	(5R,6S)-6-PHENYL-5-[4-(2-PYRROLIDIN-1-YLETHOXY)PHENYL]-5,6,7,8-TETRAHYDRONAPHTHALEN-2-OL	ant	-	-11.97
**X56**	2R6Y	[6-HYDROXY-2-(4-HYDROXYPHENYL)-1-BENZOTHIEN-3-YL][4-(2-PYRROLIDIN-1-YLETHOXY)PHENYL]METHANONE	ant	-	-11.663
**X57**	2R6W	[6-HYDROXY-2-(4-HYDROXYPHENYL)-1-BENZOTHIEN-3-YL]{4-[2-(4-METHYLPIPERIDIN-1-YL)ETHOXY]PHENYL}METHANONE	ant	-	-10.005
**X58**	1UOM	2-PHENYL-1-[4-(2-PIPERIDIN-1-YL-ETHOXY)-PHENYL]-	ant	-	-11.445

		1,2,3,4-TETRAHYDRO-ISOQUINOLIN-6-OL			
**X59**	3UUC	4,4'-(2,2-dichloroethene-1,1-diyl)diphenol	ant	-8.457	-8.391
**X60**	2Q6J	4-[(DIMESITYLBORYL)(2,2,2-TRIFLUOROETHYL)AMINO]PHENOL	ant	-	-3.319
**X61**	3ERT	4-HYDROXYTAMOXIFEN	ant	-	-11.82
**X62**	3DT3	5-(4-hydroxyphenoxy)-6-(3-hydroxyphenyl)- 7-methylnaphthalen-2-ol	ant	-9.75	-10.868
**X63**	2AYR	6-(4-METHYLSULFONYL-PHENYL)-5-[4-(2-PIPERIDIN-1-YLETHOXY)PHENOXY]NAPHTHALEN-2-OL	ant	-	-6.716
**X64**	2IOK	N-[(1R)-3-(4-HYDROXYPHENYL)-1-METHYLPROPYL]-2-(2-PHENYL-1H-INDOL-3-YL)ACETAMIDE	ant	-	-7.852
**X65**	2IOG	N-[(1R)-3-(4-HYDROXYPHENYL)-1-METHYLPROPYL]-2-[2-PHENYL-6-(2-PIPERIDIN-1-YLETHOXY)-1H-INDOL-3-YL]ACETAMIDE	ant	-	-6.825
**X66**	2QXS	RALOXIFENE	ant	-	-11.256
