# Supplementary material for: Competitive molecular docking approach for predicting estrogen receptor subtype α agonists and antagonists
Source: BMC Bioinformatics. 2014 Oct 21;15(Suppl 11):S4. doi: 10.1186/1471-2105-15-S11-S4 (PMC4251048; doi:10.1186/1471-2105-15-S11-S4)
Supplement: Additional file 2 — DUD ER ligands [file 1471-2105-15-S11-S4-S2.pdf]

Additional file 2 The ER ligands obtained from DUD used in the second set of docking as described in the study design. Information about the ligand type and docking scores in the agonist and antagonist structures are provided.

Ligand	Type	Docking Score (ago structure)	Docking Score (ant structure)
**ZINC00001219**	ago	-9.601455	-8.840287
**ZINC00039316**	ago	-9.584837	-9.156675
**ZINC00041692**	ago	-9.138191	-9.405563
**ZINC00047551**	ago	-8.976502	-8.952992
**ZINC00056434**	ago	-8.378305	-8.359926
**ZINC00056546**	ago	-7.24244	-8.871042
**ZINC00056548**	ago	-9.295089	-9.908184
**ZINC00057656**	ago	-9.768677	-9.201183
**ZINC00113304**	ago	-8.267516	-8.310766
**ZINC00155246**	ago	-6.646644	-6.812291
**ZINC00283101**	ago	-8.517315	-8.277994
**ZINC00388660**	ago	-9.212063	-8.857978
**ZINC00508310**	ago	-8.456492	-8.408264
**ZINC00585979**	ago	-9.413575	-7.196518
**ZINC01070102**	ago	-9.666618	-8.16063
**ZINC01510316**	ago	-8.423716	-8.895508
**ZINC01566834**	ago	-7.957267	-7.989849
**ZINC01587804**	ago	-8.760565	-8.849293
**ZINC01631270**	ago	-8.724366	-9.034127
**ZINC01666991**	ago	-8.16275	-9.547132
**ZINC01668370**	ago	-8.687476	-9.631634

**ZINC01686128**	ago	-8.123985	-6.037191
**ZINC01747889**	ago	-7.676667	-8.148357
**ZINC01999257**	ago	-6.497737	-4.676604
**ZINC02035359**	ago	-8.668854	-8.425483
**ZINC02048444**	ago	-8.014495	-9.210498
**ZINC02106113**	ago	-8.055275	-4.34669
**ZINC02526173**	ago	-8.571051	-8.061561
**ZINC03807917**	ago	-9.399844	-8.630396
**ZINC03812897**	ago	-9.653869	-8.033185
**ZINC03814361**	ago	-7.17977	-6.644075
**ZINC03814364**	ago	-10.556761	-8.245001
**ZINC03814379**	ago	-9.848557	-7.309332
**ZINC03814383**	ago	-7.289441	-6.722645
**ZINC03814410**	ago	-9.892839	-8.42049
**ZINC03814412**	ago	-10.006219	-8.675272
**ZINC03814415**	ago	-9.154774	-7.095231
**ZINC03814416**	ago	-9.570467	-7.257004
**ZINC03814419**	ago	-7.724644	-7.513942
**ZINC03815415**	ago	-10.477433	-7.976375
**ZINC03815416**	ago	-9.83947	-8.615452
**ZINC03815417**	ago	-9.029824	-7.705412
**ZINC03815418**	ago	-10.392001	-8.66569
**ZINC03815419**	ago	-10.712919	-7.377451
**ZINC03815421**	ago	-9.042926	-8.439475
**ZINC03815424**	ago	-8.143034	-4.169571

**ZINC03815427**	ago	-9.63573	-8.631006
**ZINC03815428**	ago	-8.043586	-7.237011
**ZINC03815429**	ago	-7.805636	-8.213421
**ZINC03815431**	ago	-10.093402	-8.43557
**ZINC03815432**	ago	-10.421544	-5.992555
**ZINC03815435**	ago	-8.882628	-9.778321
**ZINC03815437**	ago	-9.544944	-7.61157
**ZINC03815438**	ago	-9.652254	-8.93483
**ZINC03815439**	ago	-10.14124	-8.58935
**ZINC03815440**	ago	-9.067382	-6.657368
**ZINC03815441**	ago	-9.933905	-7.579918
**ZINC03815442**	ago	-9.722866	-6.866677
**ZINC03815443**	ago		-5.886944
**ZINC03815444**	ago	-9.895545	-8.845249
**ZINC03815445**	ago	-10.110234	-10.593516
**ZINC03815446**	ago	-9.882414	-9.843789
**ZINC03815448**	ago	-9.373309	-8.399811
**ZINC03815449**	ago	-9.754066	-8.948767
**ZINC03861549**	ago	-9.065207	-9.980012
**ZINC03872293**	ago	-8.006523	-9.549326
**ZINC04150452**	ago	-8.721701	-9.60442
**ZINC01530090**	ant		-9.749891
**ZINC01530602**	ant		-8.708754
**ZINC01530690**	ant		-8.665116
**ZINC01531019**	ant		-9.696979

**ZINC01543842**	ant		-9.844033
**ZINC01545572**	ant		-6.325652
**ZINC01585847**	ant		-10.969617
**ZINC01730387**	ant		
**ZINC01914469**	ant		-10.303724
**ZINC03793538**	ant		-10.028852
**ZINC03815450**	ant		-11.401375
**ZINC03815451**	ant		-11.249699
**ZINC03815452**	ant		-10.597824
**ZINC03815453**	ant		-9.880144
**ZINC03815454**	ant		-9.718972
**ZINC03815455**	ant		-10.45706
**ZINC03815456**	ant		-10.720336
**ZINC03815457**	ant		-7.058894
**ZINC03815458**	ant		-9.346332
**ZINC03815459**	ant		-8.922223
**ZINC03815460**	ant		-9.616427
**ZINC03815461**	ant		-5.82728
**ZINC03815462**	ant		-10.793351
**ZINC03815463**	ant		-8.668713
**ZINC03815464**	ant		-6.88745
**ZINC03815465**	ant		-7.152316
**ZINC03815466**	ant		-8.05994
**ZINC03815467**	ant		-7.029727
**ZINC03815468**	ant	-8.193642	-6.446163

**ZINC03815469**	ant		
**ZINC03815470**	ant		-7.142483
**ZINC03815471**	ant		-6.280203
**ZINC03815472**	ant		-6.440148
**ZINC03815473**	ant	-8.565868	-9.104681
**ZINC03815474**	ant	-8.167918	-9.125046
**ZINC03815475**	ant		-7.083903
**ZINC03815476**	ant		-6.30466
**ZINC03815477**	ant		-11.575553
**ZINC03815478**	ant		-10.330471
