# Supplementary material for: Competitive molecular docking approach for predicting estrogen receptor subtype α agonists and antagonists
Source: BMC Bioinformatics. 2014 Oct 21;15(Suppl 11):S4. doi: 10.1186/1471-2105-15-S11-S4 (PMC4251048; doi:10.1186/1471-2105-15-S11-S4)
Supplement: Additional file 3 — DUD ER decoys [file 1471-2105-15-S11-S4-S3.pdf]

Additional file 3 The ER decoys obtained from DUD used in the second set of docking as described in the study design. Information about the ligand type and docking scores in the agonist and antagonist structures are provided.

Decoy	Type	Docking Score (ago structure)	Docking Score (ant structure)
**ZINC00000189**	ago	-7.537766	-6.369726
**ZINC00000934**	ago	-6.933091	-6.605218
**ZINC00001503**	ago	-7.977738	-8.201454
**ZINC00001503**	ago	-7.791504	-7.372097
**ZINC00002614**	ago	-7.836414	-7.405175
**ZINC00002831**	ago	-8.008676	-7.56059
**ZINC00002831**	ago	-7.396932	-6.592987
**ZINC00002832**	ago	-7.867246	-7.266866
**ZINC00002832**	ago	-7.159874	-7.124577
**ZINC00002834**	ago	-7.786079	-6.071324
**ZINC00002834**	ago	-7.635594	-5.311656
**ZINC00003434**	ago	-9.452789	-9.075342
**ZINC00003629**	ago	-7.929623	-7.233312
**ZINC00003629**	ago	-7.882058	-6.270949
**ZINC00003728**	ago	-8.606748	-8.690885
**ZINC00003728**	ago	-8.21884	-6.366581
**ZINC00003760**	ago	-7.642068	-6.764295
**ZINC00003760**	ago	-7.36823	-6.241824
**ZINC00003978**	ago		-7.073991
**ZINC00004303**	ago	-7.728941	-7.00065
**ZINC00004404**	ago	-7.750292	-5.998275

**ZINC00004619**	ago	-7.497139	-6.962102
**ZINC00004619**	ago	-7.398371	-5.633707
**ZINC00004624**	ago	-8.280424	-8.022923
**ZINC00004683**	ago	-6.847266	-6.616682
**ZINC00005140**	ago	-8.370662	-8.99673
**ZINC00005569**	ago	-7.288837	-7.03941
**ZINC00005681**	ago	-8.497078	-5.964764
**ZINC00005909**	ago	-7.346581	-5.397948
**ZINC00006006**	ago	-7.690431	-6.734273
**ZINC00006052**	ago	-7.243883	-7.709493
**ZINC00006959**	ago	-7.133797	-5.766045
**ZINC00007254**	ago	-10.554454	-8.906028
**ZINC00007265**	ago	-8.045813	-6.448797
**ZINC00007287**	ago	-9.217461	-8.733399
**ZINC00007345**	ago	-10.604485	-8.972342
**ZINC00007756**	ago	-8.289922	-6.540784
**ZINC00007756**	ago	-8.21393	-6.074067
**ZINC00007756**	ago	-7.498731	-5.532389
**ZINC00007872**	ago	-7.602809	-7.708196
**ZINC00008167**	ago	-9.285134	-7.91421
**ZINC00008170**	ago	-8.041303	-8.220751
**ZINC00008269**	ago	-7.201922	-6.596402
**ZINC00008591**	ago	-6.670342	-6.127716
**ZINC00008680**	ago	-9.20865	-8.81369
**ZINC00008878**	ago	-7.885194	-6.577384

**ZINC00008950**	ago	-7.42878	-6.676564
**ZINC00009305**	ago	-9.24403	-9.6494
**ZINC00009328**	ago	-7.35419	-7.577074
**ZINC00009328**	ago	-7.328499	-7.249881
**ZINC00009328**	ago	-7.224717	-6.620443
**ZINC00009328**	ago	-7.054497	-6.055066
**ZINC00009379**	ago	-7.160046	-6.72336
**ZINC00009394**	ago	-7.320599	-7.582278
**ZINC00009603**	ago	-7.211958	-7.024704
**ZINC00009653**	ago	-7.355461	-7.376416
**ZINC00009661**	ago	-7.743803	-7.250954
**ZINC00009687**	ago	-8.644674	-9.483176
**ZINC00010950**	ago	-8.249991	-8.145359
**ZINC00011293**	ago	-7.401125	-6.532126
**ZINC00011761**	ago	-8.116924	-7.493147
**ZINC00011780**	ago	-7.735589	-7.37482
**ZINC00011787**	ago	-7.245966	-7.508479
**ZINC00011877**	ago	-6.49606	-5.676577
**ZINC00012150**	ago	-7.890918	-7.8975
**ZINC00012482**	ago	-4.928701	-5.676422
**ZINC00012591**	ago	-9.062697	-8.385165
**ZINC00012908**	ago	-7.474702	-8.000909
**ZINC00012908**	ago	-7.263722	-7.756145
**ZINC00012908**	ago	-7.187095	-7.632202
**ZINC00012908**	ago	-7.127713	-7.348214

**ZINC00013024**	ago	-7.836986	-5.068796
**ZINC00013208**	ago	-7.432233	-7.285145
**ZINC00013208**	ago	-7.2164	-6.771258
**ZINC00014171**	ago	-6.87824	-4.807027
**ZINC00015584**	ago	-6.468637	-6.464221
**ZINC00015588**	ago	-7.935518	-8.149397
**ZINC00016008**	ago	-7.276166	-4.347469
**ZINC00016144**	ago	-7.520687	-6.418942
**ZINC00016144**	ago	-7.376866	-6.213799
**ZINC00016144**	ago	-7.222494	-6.040713
**ZINC00016144**	ago	-6.69194	-5.556594
**ZINC00016716**	ago	-7.759098	-5.018839
**ZINC00017354**	ago	-9.633772	-8.202892
**ZINC00018239**	ago	-9.715375	-8.33151
**ZINC00018501**	ago	-7.192907	-7.785657
**ZINC00018758**	ago	-7.27456	-7.326894
**ZINC00019140**	ago	-8.0011	-6.260969
**ZINC00019492**	ago	-7.478869	-5.055048
**ZINC00019790**	ago	-6.466383	-2.496485
**ZINC00019791**	ago	-6.803383	-6.95635
**ZINC00020022**	ago	-8.425303	-6.658623
**ZINC00020300**	ago	-6.783888	-6.829358
**ZINC00020337**	ago	-7.601783	-8.271517
**ZINC00020469**	ago	-8.182756	-7.121019
**ZINC00021531**	ago	-7.437115	-4.794252

**ZINC00021711**	ago	-7.466006	-6.882164
**ZINC00021711**	ago	-7.466006	-6.678622
**ZINC00021711**	ago	-7.12819	-6.678622
**ZINC00022584**	ago	-9.656188	-8.84824
**ZINC00022646**	ago	-4.835904	-6.151076
**ZINC00022647**	ago	-4.521983	-6.053038
**ZINC00022649**	ago	-5.213746	-5.940992
**ZINC00022651**	ago	-6.570599	-6.192065
**ZINC00022657**	ago	-8.153323	-8.231841
**ZINC00023041**	ago	-7.378585	-5.213226
**ZINC00023042**	ago	-7.855413	-5.226309
**ZINC00023046**	ago	-8.320703	-5.1468
**ZINC00023047**	ago	-7.596348	-4.822338
**ZINC00023114**	ago	-7.821793	-5.01351
**ZINC00023114**	ago	-7.821793	-5.01351
**ZINC00023116**	ago	-7.749183	-4.235085
**ZINC00023116**	ago	-7.749183	-4.235085
**ZINC00023395**	ago	-8.900203	-7.001034
**ZINC00023979**	ago	-8.587206	-6.808757
**ZINC00024177**	ago	-7.561424	-6.961772
**ZINC00024177**	ago	-7.414601	-6.573827
**ZINC00024177**	ago	-7.275142	-5.851346
**ZINC00024178**	ago	-7.612773	-7.491015
**ZINC00024178**	ago	-7.551801	-6.68985
**ZINC00024178**	ago	-7.546845	-5.984272

**ZINC00024360**	ago	-6.882331	-6.724011
**ZINC00024362**	ago	-6.700221	-6.575356
**ZINC00024486**	ago	-8.527352	-6.906367
**ZINC00024486**	ago	-8.492163	-6.826935
**ZINC00024486**	ago	-7.254232	-6.320631
**ZINC00024486**	ago	-7.254232	-6.320631
**ZINC00024487**	ago	-8.368786	-7.556364
**ZINC00024487**	ago	-8.034557	-6.342259
**ZINC00024487**	ago	-7.9187	-6.342259
**ZINC00024487**	ago	-7.9187	-6.119517
**ZINC00025365**	ago	-7.394829	-7.272388
**ZINC00025512**	ago	-7.87987	-6.860306
**ZINC00025512**	ago	-7.40157	-6.641328
**ZINC00026164**	ago	-6.673599	-5.746238
**ZINC00026251**	ago	-9.563613	-8.5453
**ZINC00026300**	ago	-10.875184	-8.578093
**ZINC00026384**	ago	-6.729432	-6.55078
**ZINC00026416**	ago	-7.533279	-5.323671
**ZINC00026419**	ago	-7.576423	-6.362008
**ZINC00026786**	ago	-4.385009	-6.934334
**ZINC00026786**	ago	-4.198995	-5.541283
**ZINC00027212**	ago	-7.457816	-6.114144
**ZINC00032057**	ago	-6.023683	-7.272174
**ZINC00032149**	ago	-7.038015	-6.280863
**ZINC00032149**	ago	-6.288823	-6.160998

**ZINC00032843**	ago	-6.797373	-5.000541
**ZINC00033273**	ago	-8.337733	-6.560393
**ZINC00033273**	ago	-6.077016	-6.109939
**ZINC00033342**	ago	-8.037256	-7.363878
**ZINC00033767**	ago	-8.386987	-8.465172
**ZINC00033768**	ago	-7.826457	-8.03161
**ZINC00033888**	ago	-7.138511	-7.121688
**ZINC00034144**	ago	-8.01967	-8.391644
**ZINC00034146**	ago	-7.973313	-6.909759
**ZINC00034149**	ago	-8.413109	-6.884077
**ZINC00035368**	ago	-6.731522	-7.231619
**ZINC00035651**	ago	-7.210637	-5.757293
**ZINC00036452**	ago	-7.276692	-7.27299
**ZINC00036522**	ago	-8.03741	-4.790143
**ZINC00036523**	ago	-8.391047	-5.389655
**ZINC00036530**	ago	-6.841292	-7.335433
**ZINC00036531**	ago	-8.421305	-5.086435
**ZINC00036749**	ago	-6.994161	-6.759387
**ZINC00036815**	ago	-7.56837	-7.679293
**ZINC00037748**	ago	-6.619437	-5.134645
**ZINC00038124**	ago	-7.862991	-6.996765
**ZINC00038430**	ago	-6.464802	-6.860694
**ZINC00038744**	ago	-6.82978	-7.032999
**ZINC00038746**	ago	-6.934786	-6.796089
**ZINC00040761**	ago	-6.862634	

**ZINC00041023**	ago	-5.253213	-5.309402
**ZINC00041025**	ago	-6.233996	-5.724873
**ZINC00041042**	ago		-6.209228
**ZINC00041044**	ago		-5.999706
**ZINC00041448**	ago	-8.293839	-8.3856
**ZINC00041939**	ago	-7.041351	-6.469618
**ZINC00042653**	ago	-7.73669	-6.677897
**ZINC00043125**	ago	-7.19132	-6.954565
**ZINC00043770**	ago	-7.463269	-7.192299
**ZINC00043771**	ago	-7.008099	-6.837496
**ZINC00043901**	ago	-7.842424	-5.144665
**ZINC00043980**	ago	-7.164408	-6.747618
**ZINC00044300**	ago	-7.497768	-7.817677
**ZINC00045705**	ago	-7.103509	-6.096031
**ZINC00045734**	ago	-6.894601	-6.915476
**ZINC00045738**	ago	-7.298536	-6.518461
**ZINC00047293**	ago	-7.223448	-5.828046
**ZINC00047984**	ago	-7.097857	-6.410779
**ZINC00049314**	ago	-6.855085	-5.590297
**ZINC00051168**	ago	-6.646719	-7.5608
**ZINC00052758**	ago	-5.531099	-8.088723
**ZINC00053801**	ago	-8.193257	-7.830146
**ZINC00053801**	ago	-7.2768	-7.748167
**ZINC00054232**	ago	-6.482448	-5.660181
**ZINC00054337**	ago	-7.626929	-5.817426

**ZINC00054367**	ago	-5.037023	-5.413563
**ZINC00054550**	ago	-7.165809	-6.763516
**ZINC00055651**	ago	-7.780116	-5.969267
**ZINC00055848**	ago	-7.710469	-5.572293
**ZINC00057822**	ago	-7.916054	-6.193293
**ZINC00059434**	ago	-6.08494	-7.079042
**ZINC00061402**	ago	-8.101785	-4.791736
**ZINC00061403**	ago	-7.445226	-4.83161
**ZINC00061998**	ago	-7.785099	-5.103265
**ZINC00061999**	ago		-6.347736
**ZINC00062228**	ago	-8.117941	-7.989632
**ZINC00062229**	ago	-7.878508	-7.481858
**ZINC00062230**	ago	-7.876655	-7.572114
**ZINC00062231**	ago	-7.489232	-7.235241
**ZINC00063026**	ago	-6.142083	-6.175076
**ZINC00063604**	ago	-7.939274	-7.141379
**ZINC00063606**	ago	-8.901546	-7.625071
**ZINC00063861**	ago	-7.38478	-6.016406
**ZINC00066327**	ago	-8.09799	-8.029246
**ZINC00067440**	ago	-7.486862	-5.778522
**ZINC00067890**	ago	-6.785418	-6.816339
**ZINC00068857**	ago	-6.651774	-5.494598
**ZINC00069233**	ago	-8.879992	-7.010756
**ZINC00070467**	ago	-7.206434	-6.079318
**ZINC00071155**	ago	-6.409785	-4.9896

**ZINC00071496**	ago	-7.41619	-7.141624
**ZINC00071531**	ago	-6.166535	-5.801796
**ZINC00071659**	ago	-8.312357	-7.732447
**ZINC00071662**	ago	-8.492167	-7.225874
**ZINC00071730**	ago	-6.764465	-4.828348
**ZINC00071757**	ago	-7.578518	-6.73721
**ZINC00071990**	ago	-8.763291	-7.420077
**ZINC00071992**	ago	-8.200825	-7.178105
**ZINC00072899**	ago	-8.000083	-5.854154
**ZINC00072899**	ago	-7.418974	-5.6571
**ZINC00073643**	ago	-7.827934	-6.897252
**ZINC00074892**	ago	-6.490684	-5.236903
**ZINC00074973**	ago	-7.452868	-6.486828
**ZINC00075320**	ago	-6.514952	-5.537658
**ZINC00075840**	ago	-7.277227	-3.828438
**ZINC00076530**	ago	-7.437928	-6.217214
**ZINC00076968**	ago	-8.485386	-8.709812
**ZINC00078857**	ago	-6.476662	-4.634394
**ZINC00078909**	ago	-7.485595	-6.063952
**ZINC00078909**	ago	-7.25489	-5.863172
**ZINC00079216**	ago	-7.739692	-6.061707
**ZINC00079521**	ago	-7.845659	-8.375547
**ZINC00082450**	ago	-7.628616	-6.330572
**ZINC00082478**	ago	-7.458396	-4.430682
**ZINC00083128**	ago	-7.288593	-7.555334

**ZINC00083255**	ago	-6.587439	-6.284708
**ZINC00084629**	ago	-7.214957	-8.197622
**ZINC00084632**	ago	-6.87349	-6.197498
**ZINC00085198**	ago	-7.416088	-6.824548
**ZINC00086101**	ago	-5.446352	-6.310845
**ZINC00086178**	ago	-5.720105	-6.468632
**ZINC00087570**	ago	-8.037112	-7.497079
**ZINC00088513**	ago		-6.250546
**ZINC00088772**	ago	-7.949978	-7.055035
**ZINC00089835**	ago	-7.284838	-6.078443
**ZINC00090076**	ago	-7.708058	-5.190838
**ZINC00090665**	ago	-6.864228	-6.768989
**ZINC00090996**	ago	-7.714892	-6.831009
**ZINC00091194**	ago	-7.800546	-7.871513
**ZINC00091248**	ago	-6.059325	-6.541058
**ZINC00091352**	ago	-6.909723	-7.572665
**ZINC00092117**	ago	-8.215985	-6.889242
**ZINC00092324**	ago	-5.706164	-5.56294
**ZINC00092361**	ago	-7.841353	-8.060868
**ZINC00092370**	ago	-7.986012	-7.819108
**ZINC00092695**	ago	-7.638268	-8.209998
**ZINC00092695**	ago	-7.483527	-7.652718
**ZINC00093526**	ago	-8.514487	-6.865787
**ZINC00093895**	ago	-7.000728	-6.436783
**ZINC00094910**	ago	-8.458653	-7.703732

**ZINC00094915**	ago	-8.093584	-7.52802
**ZINC00098047**	ago	-8.109806	-8.28734
**ZINC00098047**	ago	-7.976751	-6.651667
**ZINC00099105**	ago	-6.626576	-5.543063
**ZINC00099109**	ago	-6.939598	-6.506322
**ZINC00100029**	ago	-5.426374	-5.549238
**ZINC00102050**	ago	-7.413996	-6.61998
**ZINC00102978**	ago	-6.784062	-5.313234
**ZINC00103432**	ago	-7.443027	-6.427055
**ZINC00104422**	ago	-7.012994	-6.810895
**ZINC00104724**	ago	-6.843716	-5.87386
**ZINC00104740**	ago	-7.520825	-5.986806
**ZINC00104760**	ago	-7.797698	-6.75809
**ZINC00104765**	ago	-7.659276	-6.828436
**ZINC00106400**	ago	-7.648808	-6.828449
**ZINC00107247**	ago	-7.989098	-8.599954
**ZINC00108286**	ago	-8.974484	-8.278845
**ZINC00110319**	ago	-8.535027	-8.280112
**ZINC00110324**	ago	-9.149077	-8.318891
**ZINC00110431**	ago	-6.945775	-8.161118
**ZINC00110434**	ago	-7.358036	-7.943989
**ZINC00110547**	ago	-8.005052	-5.865672
**ZINC00111513**	ago	-8.106417	-6.890242
**ZINC00111513**	ago	-7.805297	-6.888942
**ZINC00111788**	ago	-7.636299	-6.536649

**ZINC00111791**	ago	-7.513427	-6.724756
**ZINC00111800**	ago	-6.801129	-6.586877
**ZINC00111802**	ago	-7.097723	-6.909436
**ZINC00111829**	ago	-6.774692	-6.292788
**ZINC00111884**	ago	-7.695606	-6.941369
**ZINC00111980**	ago	-7.87041	-6.692303
**ZINC00112038**	ago	-7.571161	-6.688257
**ZINC00112345**	ago	-8.199689	-7.412068
**ZINC00112541**	ago	-7.107741	-6.47771
**ZINC00113368**	ago	-7.304416	-6.195291
**ZINC00114537**	ago	-5.428458	-6.353148
**ZINC00114841**	ago	-5.786723	-4.653658
**ZINC00116297**	ago	-7.187123	-5.036096
**ZINC00116393**	ago	-8.085036	-7.365152
**ZINC00117991**	ago	-7.584711	-6.243766
**ZINC00117991**	ago	-6.908028	-5.555692
**ZINC00121839**	ago	-7.298728	-6.794483
**ZINC00121842**	ago	-8.006361	-7.228837
**ZINC00122118**	ago	-7.288053	-7.700091
**ZINC00122667**	ago	-6.382041	-7.104082
**ZINC00122667**	ago	-6.07168	-6.455287
**ZINC00122729**	ago	-7.357547	-6.690591
**ZINC00122971**	ago	-7.535291	-7.723721
**ZINC00123258**	ago	-7.42236	-7.485686
**ZINC00123261**	ago	-7.566264	-7.155744

**ZINC00124731**	ago	-8.694899	-7.210644
**ZINC00125104**	ago	-7.694796	-6.020399
**ZINC00125584**	ago	-7.193962	-7.09169
**ZINC00126697**	ago	-7.329667	-6.864458
**ZINC00126802**	ago	-7.855671	-4.706704
**ZINC00127005**	ago	-8.631376	-6.960117
**ZINC00127200**	ago	-5.480091	-6.302733
**ZINC00127258**	ago	-6.789242	-5.774215
**ZINC00127328**	ago	-6.45573	-6.063733
**ZINC00127670**	ago	-8.032687	-6.850625
**ZINC00127670**	ago	-6.877688	-6.180805
**ZINC00127670**	ago	-5.891892	-5.725768
**ZINC00127870**	ago	-7.246815	-6.356278
**ZINC00127903**	ago	-7.483383	-6.516907
**ZINC00128048**	ago	-7.941482	-6.654945
**ZINC00128602**	ago	-7.605735	-5.060445
**ZINC00128817**	ago	-6.077172	-6.032914
**ZINC00130306**	ago	-6.383056	-6.709879
**ZINC00130309**	ago	-5.631799	-4.400138
**ZINC00130354**	ago	-5.653869	-6.38088
**ZINC00130638**	ago	-6.513077	-6.935334
**ZINC00130651**	ago	-6.610893	-6.734795
**ZINC00132311**	ago	-7.599355	-8.059337
**ZINC00132447**	ago	-7.751226	-7.98328
**ZINC00132447**	ago	-7.618877	-7.69746

**ZINC00132527**	ago	-9.106234	-8.195668
**ZINC00132529**	ago	-8.272003	-8.252115
**ZINC00133196**	ago	-7.424263	-6.242434
**ZINC00133325**	ago	-5.528786	-6.192977
**ZINC00133881**	ago	-6.205084	-6.574565
**ZINC00134376**	ago	-7.86586	-6.160599
**ZINC00134652**	ago	-7.748777	-4.182166
**ZINC00134654**	ago	-7.286831	-4.475658
**ZINC00134776**	ago	-8.263158	-7.552866
**ZINC00134776**	ago	-7.31412	-7.183362
**ZINC00134779**	ago	-7.536879	-7.024381
**ZINC00134779**	ago	-7.08793	-4.249801
**ZINC00135894**	ago	-7.139503	-6.46797
**ZINC00135905**	ago	-7.346406	-2.686853
**ZINC00135906**	ago	-5.847652	-6.353949
**ZINC00136675**	ago	-7.004864	-5.567489
**ZINC00136818**	ago	-7.39782	-5.676289
**ZINC00136818**	ago	-6.694881	-5.337402
**ZINC00137051**	ago	-8.108647	-6.437158
**ZINC00137178**	ago	-7.506198	-7.419686
**ZINC00137198**	ago	-7.237295	-6.709551
**ZINC00138773**	ago	-9.306106	-9.214714
**ZINC00138785**	ago	-8.017362	-8.119769
**ZINC00138880**	ago	-8.143736	-6.365559
**ZINC00138891**	ago	-7.50226	-6.728459

**ZINC00139529**	ago	-7.200579	-6.739913
**ZINC00140314**	ago	-8.125934	-5.476613
**ZINC00140318**	ago	-8.169873	-4.002626
**ZINC00143973**	ago	-7.806033	-8.418512
**ZINC00144143**	ago	-6.871352	-6.386525
**ZINC00145938**	ago	-7.823089	-8.545417
**ZINC00146196**	ago	-6.313452	-6.057705
**ZINC00146248**	ago	-7.331064	-7.056615
**ZINC00147726**	ago	-6.17028	-6.004597
**ZINC00148656**	ago	-7.943891	-6.604949
**ZINC00148883**	ago	-8.006651	-6.857251
**ZINC00148912**	ago	-7.921841	-6.027744
**ZINC00150675**	ago	-7.650876	-8.010682
**ZINC00150676**	ago	-7.280554	-7.309894
**ZINC00150939**	ago	-7.815778	-6.155669
**ZINC00151809**	ago	-6.873172	-5.758875
**ZINC00152683**	ago	-5.585488	-5.679957
**ZINC00153269**	ago	-6.295619	-5.810163
**ZINC00157026**	ago	-5.449656	-6.279506
**ZINC00157694**	ago	-6.306411	-5.50405
**ZINC00159164**	ago	-8.53628	-6.904808
**ZINC00161107**	ago	-7.274306	-5.491607
**ZINC00162703**	ago	-7.62047	-6.298109
**ZINC00162705**	ago	-7.187741	-5.786872
**ZINC00162707**	ago	-7.641912	-6.212664

**ZINC00164865**	ago	-8.148854	-4.495436
**ZINC00165974**	ago	-7.272374	-4.142598
**ZINC00166050**	ago	-8.022176	-7.64311
**ZINC00167632**	ago	-7.363826	-7.48822
**ZINC00167789**	ago	-7.858969	-4.885105
**ZINC00168076**	ago	-7.011738	-6.987433
**ZINC00169221**	ago	-8.146255	-6.905609
**ZINC00169549**	ago	-7.394902	-5.561507
**ZINC00169728**	ago	-6.952417	-6.810192
**ZINC00172006**	ago	-5.755981	-6.131574
**ZINC00172324**	ago	-7.301361	-5.69608
**ZINC00172332**	ago	-7.01622	-4.508647
**ZINC00173761**	ago	-8.295829	-8.350725
**ZINC00173989**	ago	-9.088239	-7.37964
**ZINC00174051**	ago		-3.383885
**ZINC00174901**	ago	-6.302033	-6.212299
**ZINC00175449**	ago	-6.88565	-6.028121
**ZINC00175821**	ago	-6.995341	-5.393476
**ZINC00175943**	ago	-6.259124	-5.647912
**ZINC00177986**	ago	-7.290263	-7.420423
**ZINC00177986**	ago	-7.236073	-7.158893
**ZINC00178238**	ago	-5.783994	-5.496375
**ZINC00178248**	ago	-5.531008	-5.547727
**ZINC00178800**	ago	-7.099844	-6.745114
**ZINC00179164**	ago	-6.088343	-6.323155

**ZINC00179241**	ago	-7.414871	-4.992161
**ZINC00179288**	ago	-7.466495	-5.47228
**ZINC00179289**	ago	-8.326775	-5.95062
**ZINC00179291**	ago	-8.153274	-7.201317
**ZINC00179291**	ago	-7.844182	-5.732695
**ZINC00180621**	ago	-7.406402	-7.364246
**ZINC00180650**	ago	-7.053309	-6.772388
**ZINC00181419**	ago	-5.883968	-5.650307
**ZINC00181868**	ago	-6.708294	-6.703296
**ZINC00182337**	ago	-6.340833	-5.691444
**ZINC00184134**	ago	-6.208945	-4.505206
**ZINC00184560**	ago	-6.844468	-7.946969
**ZINC00184583**	ago	-7.546549	-7.629372
**ZINC00184584**	ago	-7.768381	-6.865171
**ZINC00184724**	ago	-8.147042	-7.472055
**ZINC00185159**	ago	-7.217058	-4.34014
**ZINC00185169**	ago	-6.880332	-4.665538
**ZINC00185696**	ago	-8.222348	-7.017202
**ZINC00186075**	ago	-7.905463	-5.060485
**ZINC00186390**	ago	-7.950415	-5.748574
**ZINC00186417**	ago	-6.538206	-5.835299
**ZINC00186475**	ago	-7.723465	-6.83443
**ZINC00186504**	ago	-7.346327	-5.502323
**ZINC00186631**	ago	-7.654958	-6.659044
**ZINC00186643**	ago	-8.603614	-5.7412

**ZINC00188889**	ago	-7.512242	-6.683844
**ZINC00191197**	ago	-7.664328	-6.721974
**ZINC00191620**	ago	-5.705444	-5.549364
**ZINC00191880**	ago	-5.980783	-6.349441
**ZINC00192127**	ago	-6.923402	-6.069165
**ZINC00192477**	ago	-4.629779	-6.302479
**ZINC00194010**	ago	-7.027101	-6.982899
**ZINC00194013**	ago	-7.188998	-7.476594
**ZINC00194058**	ago	-7.833215	-8.018827
**ZINC00194603**	ago	-7.269283	-6.323007
**ZINC00194767**	ago	-7.04713	-6.439691
**ZINC00195023**	ago	-7.864155	-4.834201
**ZINC00199617**	ago	-7.193762	-6.734222
**ZINC00199651**	ago	-7.494013	-6.826611
**ZINC00204006**	ago	-8.294699	-7.419612
**ZINC00207253**	ago	-8.063	-7.108594
**ZINC00208671**	ago	-8.549671	-6.8762
**ZINC00209577**	ago	-7.046525	-7.08458
**ZINC00210209**	ago	-7.897093	-7.062496
**ZINC00210209**	ago	-7.589572	-6.208622
**ZINC00210340**	ago	-7.332525	-7.061295
**ZINC00210711**	ago	-6.81738	-5.367283
**ZINC00210799**	ago	-6.604394	-5.266355
**ZINC00213316**	ago	-7.549839	-6.044108
**ZINC00213324**	ago	-6.112542	-6.34675

**ZINC00213463**	ago	-8.435416	-4.37117
**ZINC00213488**	ago	-8.492923	-4.970194
**ZINC00215530**	ago	-7.112552	-5.053333
**ZINC00217234**	ago	-7.333736	-7.208604
**ZINC00217349**	ago		
**ZINC00222225**	ago	-8.29324	-7.483151
**ZINC00223312**	ago	-6.501468	-6.464826
**ZINC00223334**	ago	-6.293016	-6.363734
**ZINC00224129**	ago	-6.23556	-7.313819
**ZINC00224260**	ago	-6.81012	-6.209369
**ZINC00224610**	ago	-7.228832	-6.826724
**ZINC00225299**	ago	-6.759934	-6.96898
**ZINC00225343**	ago	-6.443064	-6.836962
**ZINC00225461**	ago	-6.765486	-7.342253
**ZINC00225610**	ago	-9.463553	-9.083282
**ZINC00226062**	ago	-8.453243	-7.529969
**ZINC00226379**	ago	-6.701131	-6.441768
**ZINC00226425**	ago	-6.979988	-6.62796
**ZINC00226846**	ago	-7.835227	-7.09908
**ZINC00227300**	ago	-6.305288	-4.334424
**ZINC00227333**	ago	-7.126188	-7.100127
**ZINC00227419**	ago	-6.601807	-5.536609
**ZINC00227433**	ago	-7.775423	-6.658971
**ZINC00227435**	ago	-8.164376	-7.357771
**ZINC00227773**	ago	-8.307448	-7.72478

**ZINC00228227**	ago	-6.964394	-4.751451
**ZINC00228272**	ago	-7.478854	-5.674808
**ZINC00228351**	ago	-6.945531	-6.124034
**ZINC00229083**	ago	-8.175662	-7.28813
**ZINC00229272**	ago	-6.289765	-5.717527
**ZINC00229306**	ago	-6.844721	-6.65791
**ZINC00229322**	ago	-6.472552	-5.79073
**ZINC00229627**	ago	-7.157577	-6.690004
**ZINC00229918**	ago	-7.565377	-5.488046
**ZINC00230012**	ago	-7.707471	-4.21395
**ZINC00230495**	ago	-7.557046	-6.615151
**ZINC00230496**	ago	-7.285511	-6.665096
**ZINC00230498**	ago	-7.625386	-6.372441
**ZINC00232436**	ago	-7.918397	-5.586724
**ZINC00232969**	ago	-7.189241	-6.583598
**ZINC00232994**	ago	-7.507761	-6.074591
**ZINC00232994**	ago	-6.993134	-5.419796
**ZINC00233058**	ago	-7.344011	-5.917339
**ZINC00233102**	ago	-7.373781	-6.041018
**ZINC00233102**	ago	-6.1264	-5.331069
**ZINC00234068**	ago	-7.158355	-6.140065
**ZINC00234075**	ago	-7.568936	-6.087192
**ZINC00234806**	ago	-7.137223	-4.3721
**ZINC00234809**	ago	-7.17765	-7.264152
**ZINC00234811**	ago	-7.79165	-6.750382

**ZINC00234816**	ago	-7.576453	-7.386001
**ZINC00235311**	ago	-7.069748	-6.648527
**ZINC00235359**	ago	-8.000118	-6.812243
**ZINC00235823**	ago	-9.119629	-9.013125
**ZINC00236108**	ago	-7.90662	-7.304895
**ZINC00236112**	ago	-8.196387	-7.515275
**ZINC00236871**	ago		-6.70049
**ZINC00236871**	ago		-6.201029
**ZINC00238014**	ago	-7.826259	-5.45567
**ZINC00238473**	ago	-7.757587	-7.390089
**ZINC00238763**	ago	-6.376627	-6.448308
**ZINC00238764**	ago	-6.790191	-6.643433
**ZINC00239709**	ago	-7.399915	-6.886572
**ZINC00241870**	ago	-7.500323	-7.280653
**ZINC00243533**	ago	-5.483396	-6.564788
**ZINC00244673**	ago	-7.632083	-7.3499
**ZINC00245783**	ago	-8.645437	-7.326945
**ZINC00245828**	ago	-8.173588	-5.801404
**ZINC00246384**	ago	-6.118586	-6.280064
**ZINC00246386**	ago	-6.822398	-5.914958
**ZINC00246653**	ago	-8.127387	-6.996523
**ZINC00246658**	ago	-7.353671	-6.957278
**ZINC00246668**	ago	-8.111815	-6.359299
**ZINC00246672**	ago	-6.879695	-6.383175
**ZINC00246737**	ago	-6.843695	-7.311273

**ZINC00246743**	ago	-7.394866	-6.751065
**ZINC00246756**	ago	-6.313926	-7.234082
**ZINC00246898**	ago	-6.890437	-6.193187
**ZINC00247015**	ago	-6.749627	-5.187262
**ZINC00250221**	ago	-8.143649	-6.958898
**ZINC00250228**	ago	-8.749604	-7.728701
**ZINC00250236**	ago	-5.710964	-6.089373
**ZINC00250259**	ago	-8.37351	-7.808015
**ZINC00250266**	ago	-8.655055	-7.809489
**ZINC00250698**	ago	-6.746903	-5.319712
**ZINC00250997**	ago	-6.122459	-6.294888
**ZINC00251012**	ago	-6.295016	-5.979469
**ZINC00252220**	ago	-6.187803	-5.176335
**ZINC00257145**	ago	-6.914841	-6.144898
**ZINC00257180**	ago	-6.701545	-6.733174
**ZINC00257645**	ago	-7.119689	-6.551956
**ZINC00257645**	ago	-6.764704	-6.15072
**ZINC00257739**	ago	-6.857663	-6.503661
**ZINC00257868**	ago	-7.010297	-6.796162
**ZINC00258382**	ago	-6.732438	-6.032244
**ZINC00258914**	ago	-6.38103	-5.642814
**ZINC00259547**	ago	-6.940786	-5.922424
**ZINC00261578**	ago	-7.478452	-5.323104
**ZINC00262378**	ago	-7.605011	-7.115287
**ZINC00262587**	ago	-8.632305	-4.586109

**ZINC00263250**	ago	-6.65616	-6.762075
**ZINC00263280**	ago	-6.7382	-6.485068
**ZINC00264282**	ago	-5.909184	-4.973805
**ZINC00264285**	ago	-4.936081	-5.052006
**ZINC00265009**	ago	-5.04588	-5.597006
**ZINC00265241**	ago	-6.423273	-5.39754
**ZINC00265802**	ago	-7.960274	-4.747277
**ZINC00265805**	ago	-7.34919	-6.051752
**ZINC00268518**	ago	-4.887794	-7.19036
**ZINC00268704**	ago	-8.860597	-7.984298
**ZINC00268708**	ago	-8.364335	-8.152739
**ZINC00269450**	ago	-5.869318	-6.312423
**ZINC00269608**	ago	-7.386466	-6.254557
**ZINC00270572**	ago	-6.810446	-6.192115
**ZINC00271435**	ago	-6.994678	-6.920136
**ZINC00271538**	ago	-6.226356	-5.680495
**ZINC00271772**	ago	-6.54839	-5.245417
**ZINC00271980**	ago	-6.295597	-6.644754
**ZINC00272213**	ago	-6.249941	-5.530505
**ZINC00272299**	ago	-7.804518	-6.250122
**ZINC00272602**	ago	-6.327363	-6.951403
**ZINC00272668**	ago	-7.352999	-6.695659
**ZINC00272680**	ago	-7.702263	-3.736431
**ZINC00273511**	ago	-6.706087	-5.336488
**ZINC00275194**	ago	-7.917846	-4.521581

**ZINC00277992**	ago	-7.409182	-5.581761
**ZINC00279589**	ago	-6.099443	-6.423543
**ZINC00279679**	ago		-6.111973
**ZINC00279792**	ago	-8.316705	-6.479694
**ZINC00279800**	ago	-8.147308	-7.158134
**ZINC00279868**	ago	-8.141121	-7.807689
**ZINC00280009**	ago	-8.207346	-6.379434
**ZINC00280009**	ago	-6.855739	-5.477582
**ZINC00280240**	ago	-7.138562	-7.537769
**ZINC00280313**	ago	-8.223982	-7.112823
**ZINC00280320**	ago	-8.038258	-5.695934
**ZINC00280330**	ago	-8.294216	-7.399746
**ZINC00280652**	ago	-7.148554	-4.978554
**ZINC00282372**	ago	-5.728447	-6.097674
**ZINC00282386**	ago	-7.065927	-6.947711
**ZINC00282386**	ago	-6.676618	-6.335947
**ZINC00282477**	ago	-8.021812	-6.771711
**ZINC00283151**	ago	-7.154947	-4.548013
**ZINC00283210**	ago	-6.742344	-6.501737
**ZINC00284075**	ago	-7.745685	-4.463336
**ZINC00284271**	ago	-5.721416	-6.5314
**ZINC00285876**	ago	-6.952936	-5.436908
**ZINC00287361**	ago	-7.658037	-7.323588
**ZINC00287363**	ago	-7.687726	-7.420063
**ZINC00288555**	ago	-6.943941	-5.607643

**ZINC00289653**	ago	-7.28582	-6.708155
**ZINC00290540**	ago	-7.005928	-5.954203
**ZINC00291349**	ago	-6.277755	-5.352747
**ZINC00291625**	ago	-6.756064	-5.050486
**ZINC00292702**	ago	-7.430909	-7.516027
**ZINC00293942**	ago	-7.876673	-7.387591
**ZINC00293945**	ago	-8.337352	-7.8916
**ZINC00296603**	ago	-7.081881	-7.380359
**ZINC00297090**	ago	-7.632131	-4.818616
**ZINC00297106**	ago	-7.763215	-6.766658
**ZINC00297109**	ago	-8.077331	-7.889385
**ZINC00297326**	ago	-8.120914	-6.922121
**ZINC00297328**	ago	-6.834133	-6.491075
**ZINC00297668**	ago	-6.567673	-7.241012
**ZINC00297690**	ago	-8.432514	-7.933776
**ZINC00300497**	ago	-6.992358	-4.41786
**ZINC00300577**	ago	-6.241253	-5.713737
**ZINC00300674**	ago	-7.262154	-5.103454
**ZINC00300945**	ago	-7.784341	-5.994355
**ZINC00300950**	ago	-7.943299	-7.041898
**ZINC00302078**	ago	-8.41356	-8.98212
**ZINC00305185**	ago	-7.657526	-4.272751
**ZINC00305188**	ago	-7.392276	-5.318354
**ZINC00305251**	ago	-7.514768	-6.769931
**ZINC00305251**	ago	-7.163881	-6.248563

**ZINC00307652**	ago	-7.667673	-6.839331
**ZINC00309284**	ago	-7.523622	-6.366258
**ZINC00309912**	ago	-7.223119	-6.64331
**ZINC00310980**	ago	-7.687519	-5.563949
**ZINC00311200**	ago	-5.359575	-5.496749
**ZINC00311393**	ago	-7.18179	-7.231552
**ZINC00311467**	ago	-7.574746	-7.738701
**ZINC00311482**	ago	-7.337456	-6.385295
**ZINC00312965**	ago	-7.657172	-6.568514
**ZINC00313435**	ago	-6.416179	-6.407077
**ZINC00314066**	ago	-7.835782	-6.793476
**ZINC00314940**	ago	-7.864496	-6.627196
**ZINC00315507**	ago	-7.653358	-6.758826
**ZINC00316745**	ago	-6.064104	-5.989766
**ZINC00317099**	ago	-7.104155	-6.471773
**ZINC00317852**	ago	-8.44829	-7.561338
**ZINC00318172**	ago	-5.653578	-7.270161
**ZINC00320812**	ago	-6.742116	-4.666311
**ZINC00323862**	ago	-8.938812	-8.38418
**ZINC00330816**	ago	-6.167739	-6.157763
**ZINC00330819**	ago	-5.33578	-5.926849
**ZINC00330864**	ago	-6.363977	-7.178981
**ZINC00331447**	ago	-7.005306	-5.686095
**ZINC00331504**	ago	-7.001664	-5.319474
**ZINC00331931**	ago	-8.039069	-4.855426

**ZINC00335546**	ago	-6.592383	-6.496331
**ZINC00336226**	ago	-7.275972	-5.893384
**ZINC00336226**	ago	-7.056822	-5.477312
**ZINC00336612**	ago	-6.568903	-6.6817
**ZINC00337522**	ago	-7.392861	-6.848434
**ZINC00337860**	ago	-6.986599	-6.562125
**ZINC00338073**	ago	-5.199446	-6.988913
**ZINC00338118**	ago	-8.648697	-6.732061
**ZINC00338553**	ago	-8.102125	-7.440361
**ZINC00339976**	ago	-7.203248	-6.208129
**ZINC00339977**	ago	-6.850982	-6.810372
**ZINC00339978**	ago	-7.367661	-6.52368
**ZINC00339979**	ago	-6.119994	-6.897165
**ZINC00339980**	ago	-7.147932	-5.959429
**ZINC00339983**	ago	-6.290528	-6.798091
**ZINC00341187**	ago	-6.872132	-6.474517
**ZINC00341188**	ago	-5.482112	-6.124355
**ZINC00342010**	ago	-3.164533	-4.740278
**ZINC00342061**	ago		-4.963169
**ZINC00342644**	ago	-5.740974	-5.774856
**ZINC00342721**	ago	-7.377308	-7.075447
**ZINC00342723**	ago	-7.026998	-7.012094
**ZINC00344025**	ago	-7.55324	-7.407111
**ZINC00344025**	ago	-7.531997	-7.079754
**ZINC00344401**	ago	-7.158818	-6.608429

**ZINC00346288**	ago	-7.47443	-7.441495
**ZINC00346712**	ago	-7.752097	-7.043462
**ZINC00346712**	ago	-7.563723	-7.042614
**ZINC00347452**	ago	-6.458854	-8.022673
**ZINC00349381**	ago	-7.255224	-6.59413
**ZINC00351100**	ago	-7.582852	-6.516182
**ZINC00354239**	ago	-8.463995	-7.50177
**ZINC00354622**	ago	-7.791761	-7.774565
**ZINC00354625**	ago	-7.011646	-7.156961
**ZINC00357998**	ago	-7.855031	-6.567685
**ZINC00358181**	ago	-8.415883	-6.930471
**ZINC00358184**	ago	-6.355087	-6.45677
**ZINC00360098**	ago	-8.731886	-7.219257
**ZINC00362700**	ago	-5.343896	-6.838798
**ZINC00363836**	ago	-5.33539	-6.650266
**ZINC00364397**	ago	-5.174859	-6.613757
**ZINC00364511**	ago	-5.796402	-6.20922
**ZINC00365219**	ago	-8.382341	-8.09386
**ZINC00365445**	ago	-7.513318	-7.913083
**ZINC00365447**	ago	-7.361327	-7.4952
**ZINC00366655**	ago	-7.691762	-7.473184
**ZINC00367769**	ago	-7.762331	-7.06489
**ZINC00367771**	ago	-7.42738	-7.101475
**ZINC00367848**	ago	-5.656466	-5.679326
**ZINC00368040**	ago	-6.987436	-6.131401

**ZINC00370585**	ago	-8.005388	-7.60615
**ZINC00370586**	ago	-7.689048	-6.236954
**ZINC00373926**	ago	-7.876768	-6.576258
**ZINC00373968**	ago	-6.699664	-5.270433
**ZINC00373971**	ago	-7.240547	-5.592001
**ZINC00374072**	ago	-7.344492	-5.120538
**ZINC00374472**	ago	-7.174385	-6.44912
**ZINC00374521**	ago	-7.830801	-6.98469
**ZINC00375044**	ago	-6.861104	-6.733787
**ZINC00380387**	ago	-7.174184	-6.750276
**ZINC00381509**	ago	-7.734254	-5.648533
**ZINC00381510**	ago	-7.250605	-5.603763
**ZINC00383790**	ago	-4.561449	-6.577973
**ZINC00383791**	ago	-5.568891	-6.735611
**ZINC00383792**	ago	-5.046456	-5.696049
**ZINC00385818**	ago	-6.726456	-6.450971
**ZINC00385818**	ago	-6.597463	-6.064966
**ZINC00386224**	ago	-6.482258	-5.669912
**ZINC00386458**	ago	-7.021159	-6.977714
**ZINC00386528**	ago	-7.238111	-5.419274
**ZINC00386529**	ago	-7.377695	-6.736691
**ZINC00386540**	ago	-8.129891	-7.778972
**ZINC00386540**	ago	-7.749965	-7.580438
**ZINC00387281**	ago	-6.112656	-5.402661
**ZINC00387351**	ago	-8.519625	-7.87113

**ZINC00387810**	ago	-7.861919	-6.846167
**ZINC00387859**	ago	-7.617514	-7.134643
**ZINC00387860**	ago	-7.025504	-7.43809
**ZINC00387861**	ago	-8.092639	-7.14496
**ZINC00387862**	ago	-7.974044	-7.45917
**ZINC00389470**	ago	-7.608912	-4.379611
**ZINC00389943**	ago	-7.779411	-7.240772
**ZINC00390200**	ago	-7.740256	-7.087133
**ZINC00390200**	ago	-7.175266	-5.361413
**ZINC00390202**	ago	-8.028381	-7.372295
**ZINC00390202**	ago	-7.35542	-4.89744
**ZINC00390238**	ago	-7.838705	-5.299788
**ZINC00390280**	ago	-9.393048	-9.206467
**ZINC00390350**	ago	-8.289933	-9.239194
**ZINC00390467**	ago	-7.734089	-7.999287
**ZINC00390822**	ago	-7.345368	-5.407662
**ZINC00392237**	ago	-7.02836	-6.769731
**ZINC00392507**	ago	-7.442833	-5.40589
**ZINC00392984**	ago	-8.225348	-5.700582
**ZINC00393543**	ago	-5.68953	-6.162371
**ZINC00393907**	ago	-6.602845	-6.652541
**ZINC00398505**	ago	-8.192818	-7.190683
**ZINC00398617**	ago	-7.322661	-6.073188
**ZINC00399189**	ago	-7.965343	-7.436232
**ZINC00399905**	ago	-8.239572	-7.771072

**ZINC00400425**	ago	-6.398796	-6.577231
**ZINC00403003**	ago	-8.26293	-7.491965
**ZINC00405331**	ago	-7.184711	-6.103126
**ZINC00407239**	ago	-7.675994	-6.603668
**ZINC00408148**	ago	-7.774153	-7.530066
**ZINC00408148**	ago	-7.380423	-5.460864
**ZINC00411147**	ago	-7.583041	-8.023075
**ZINC00413675**	ago	-9.185356	-8.945172
**ZINC00413675**	ago	-9.151574	-8.880735
**ZINC00413684**	ago	-8.094156	-8.258661
**ZINC00413684**	ago	-7.653081	-7.839371
**ZINC00413697**	ago	-8.147501	-8.057277
**ZINC00413697**	ago	-7.938333	-8.053909
**ZINC00413699**	ago	-9.090267	-9.038788
**ZINC00413699**	ago	-8.723189	-8.995952
**ZINC00413712**	ago	-8.340558	-8.151115
**ZINC00413712**	ago	-8.235158	-6.60353
**ZINC00413717**	ago	-8.695077	-7.612922
**ZINC00413717**	ago	-8.558706	-6.764593
**ZINC00414588**	ago	-7.291997	-6.43549
**ZINC00415535**	ago	-7.556704	-6.663016
**ZINC00416281**	ago	-8.942564	-7.844384
**ZINC00416281**	ago	-8.697607	-7.375061
**ZINC00416283**	ago	-8.925967	-8.764307
**ZINC00416283**	ago	-8.712048	-8.661033

**ZINC00416285**	ago	-7.930799	-8.151953
**ZINC00416285**	ago	-7.904968	-7.817174
**ZINC00416286**	ago	-8.994014	-8.127034
**ZINC00416286**	ago	-8.767003	-8.072202
**ZINC00416658**	ago	-7.588682	-4.713185
**ZINC00417878**	ago	-8.288207	-8.620386
**ZINC00417878**	ago	-8.18766	-8.497639
**ZINC00418205**	ago	-6.21143	-5.961619
**ZINC00419082**	ago	-8.179476	-7.964315
**ZINC00420811**	ago	-6.691364	-6.58586
**ZINC00420824**	ago		-5.212027
**ZINC00422990**	ago	-6.830824	-6.694747
**ZINC00423030**	ago	-8.191129	-7.499602
**ZINC00423349**	ago	-7.12891	-6.144461
**ZINC00424540**	ago	-8.093978	-6.774343
**ZINC00427639**	ago	-6.106014	-7.129262
**ZINC00428762**	ago	-8.000434	-7.685844
**ZINC00428763**	ago	-7.888531	-8.231129
**ZINC00428764**	ago	-7.496913	-7.268453
**ZINC00429463**	ago	-6.154265	-5.685006
**ZINC00429497**	ago	-7.916881	-5.818542
**ZINC00429523**	ago	-7.50282	-5.929222
**ZINC00431557**	ago	-8.231095	-6.129676
**ZINC00431803**	ago	-6.784214	-3.464942
**ZINC00432422**	ago	-7.158902	-6.488043

**ZINC00432423**	ago	-7.783482	-6.70757
**ZINC00432636**	ago	-5.087669	-5.821935
**ZINC00432670**	ago	-7.366274	-7.737775
**ZINC00433585**	ago	-6.27227	-6.886773
**ZINC00433606**	ago	-6.160925	-6.734897
**ZINC00433715**	ago	-6.650152	-6.010829
**ZINC00434183**	ago	-6.708278	-5.913042
**ZINC00435701**	ago	-6.913983	-3.724484
**ZINC00436013**	ago	-6.814073	-7.774924
**ZINC00436303**	ago	-7.027199	-6.123811
**ZINC00436303**	ago	-6.82535	-5.962318
**ZINC00436528**	ago	-4.687046	-6.344171
**ZINC00437295**	ago	-7.358793	-5.926218
**ZINC00437528**	ago	-7.596146	-6.022493
**ZINC00437597**	ago	-7.910671	-7.650286
**ZINC00437811**	ago	-7.345912	-5.579832
**ZINC00438899**	ago	-7.386911	-6.962958
**ZINC00438899**	ago	-6.837202	-6.725596
**ZINC00439137**	ago	-7.223333	-6.895971
**ZINC00440469**	ago	-6.413889	-7.023278
**ZINC00441501**	ago	-6.868607	-7.101826
**ZINC00441601**	ago	-5.821808	-5.813952
**ZINC00442718**	ago	-7.274737	-6.831117
**ZINC00442789**	ago	-7.458516	-7.159552
**ZINC00443255**	ago	-7.312317	-6.428691

**ZINC00443411**	ago	-8.904163	-7.382854
**ZINC00443515**	ago	-8.847785	-8.556456
**ZINC00445513**	ago	-5.745982	-4.558602
**ZINC00445541**	ago	-5.256741	-6.706757
**ZINC00447748**	ago	-6.734461	-5.118289
**ZINC00448984**	ago	-7.343637	-7.290501
**ZINC00451771**	ago	-7.770086	-6.71457
**ZINC00452022**	ago	-7.020785	-6.851516
**ZINC00452350**	ago	-7.301467	-6.448534
**ZINC00452606**	ago	-7.202852	-6.881443
**ZINC00452607**	ago	-7.425425	-6.436366
**ZINC00452658**	ago	-5.443895	-6.510481
**ZINC00452658**	ago	-5.365971	-6.462209
**ZINC00452658**	ago	-5.166243	-6.122234
**ZINC00454156**	ago	-6.751879	-7.033547
**ZINC00456090**	ago	-7.471619	-7.832876
**ZINC00461058**	ago	-6.433567	-6.035199
**ZINC00463113**	ago	-7.284669	-5.036464
**ZINC00463525**	ago	-7.082654	-5.567081
**ZINC00463755**	ago	-8.261678	-5.001342
**ZINC00464164**	ago	-8.010738	-6.455844
**ZINC00464494**	ago	-7.058796	-4.064923
**ZINC00466494**	ago	-5.836189	-5.720343
**ZINC00466508**	ago	-7.43744	-5.948869
**ZINC00469488**	ago	-8.288915	-7.759924

**ZINC00474273**	ago	-7.225703	-3.794468
**ZINC00476230**	ago	-7.057271	-6.472462
**ZINC00476754**	ago	-7.37241	-6.882456
**ZINC00477411**	ago	-6.879118	-5.735733
**ZINC00477507**	ago	-8.58341	-4.598537
**ZINC00478414**	ago	-7.75456	-4.952915
**ZINC00482797**	ago	-7.076526	-7.371218
**ZINC00482802**	ago	-5.972023	-5.735568
**ZINC00482806**	ago	-7.42594	-5.85536
**ZINC00483921**	ago	-8.139047	-7.611046
**ZINC00483922**	ago	-8.546064	-8.150407
**ZINC00484450**	ago	-6.715381	-5.777097
**ZINC00484452**	ago	-6.312794	-6.246306
**ZINC00485045**	ago	-8.626835	-4.075478
**ZINC00485084**	ago	-6.06696	-5.750632
**ZINC00485402**	ago	-6.697703	-4.960929
**ZINC00485689**	ago	-7.523924	-7.418763
**ZINC00486064**	ago	-7.657881	-7.481659
**ZINC00486129**	ago	-6.881534	-4.302692
**ZINC00486185**	ago	-7.954693	-7.95024
**ZINC00487022**	ago	-7.315247	-5.233387
**ZINC00487312**	ago	-7.459826	-8.308504
**ZINC00487650**	ago	-9.061667	-9.012914
**ZINC00487938**	ago	-8.145478	-8.423622
**ZINC00491454**	ago	-7.346087	-7.599851

**ZINC00492807**	ago	-7.525287	-4.597522
**ZINC00495239**	ago	-7.716684	-6.88327
**ZINC00495265**	ago	-7.027501	-7.266169
**ZINC00495351**	ago	-6.896101	-6.122614
**ZINC00497884**	ago	-7.506663	-8.221159
**ZINC00500205**	ago	-8.222959	-7.642814
**ZINC00501131**	ago	-7.030822	-7.558823
**ZINC00501132**	ago	-6.755014	-6.395313
**ZINC00501370**	ago	-7.842837	-7.564653
**ZINC00501522**	ago	-8.194284	-4.234044
**ZINC00501667**	ago	-6.337426	-6.753137
**ZINC00501750**	ago	-6.724752	-5.066596
**ZINC00501752**	ago	-5.924359	-5.199733
**ZINC00501945**	ago	-6.339257	-5.35789
**ZINC00502312**	ago	-5.8342	-6.17934
**ZINC00502312**	ago	-5.557552	-6.008782
**ZINC00502312**	ago	-5.548152	-5.861873
**ZINC00502653**	ago		-5.13214
**ZINC00502655**	ago		-4.804725
**ZINC00502932**	ago	-6.427492	-3.038781
**ZINC00503073**	ago	-6.096452	-6.482954
**ZINC00503181**	ago	-6.985224	-4.277796
**ZINC00503365**	ago	-6.893284	-6.264115
**ZINC00503366**	ago	-7.032334	-6.999939
**ZINC00504692**	ago	-6.34906	-6.559763

**ZINC00505704**	ago	-5.155995	-5.68866
**ZINC00505712**	ago	-5.486113	-5.998984
**ZINC00505728**	ago	-5.766356	-5.85685
**ZINC00507019**	ago	-9.207599	-7.941995
**ZINC00507019**	ago	-8.624114	-7.38932
**ZINC00507019**	ago	-8.310487	-7.220911
**ZINC00508501**	ago	-6.353322	
**ZINC00509885**	ago		-7.611647
**ZINC00510125**	ago	-8.346994	-8.231514
**ZINC00510125**	ago	-7.961333	-7.421592
**ZINC00510393**	ago	-8.426421	-6.42718
**ZINC00510906**	ago	-8.183719	-7.739794
**ZINC00510907**	ago	-7.654226	-4.662143
**ZINC00510990**	ago	-6.909899	-6.022274
**ZINC00511107**	ago	-7.406393	-6.311803
**ZINC00512594**	ago	-6.309338	-5.989133
**ZINC00513277**	ago	-4.153223	-3.345316
**ZINC00513288**	ago	-5.525271	-6.872569
**ZINC00513294**	ago	-7.62417	-5.999837
**ZINC00513296**	ago	-6.774887	
**ZINC00513297**	ago	-6.632478	-4.709388
**ZINC00513684**	ago	-7.937356	-5.045
**ZINC00513685**	ago	-7.486152	-6.614298
**ZINC00514644**	ago	-6.500057	-5.791259
**ZINC00518961**	ago	-8.969121	-8.474419

**ZINC00519450**	ago	-9.408551	-7.728518
**ZINC00519451**	ago	-9.383018	-7.685558
**ZINC00519490**	ago	-7.793675	-7.252273
**ZINC00519491**	ago	-8.36245	-7.065025
**ZINC00519579**	ago	-6.837207	-5.137399
**ZINC00519667**	ago		-5.837915
**ZINC00519960**	ago	-6.986065	-6.779336
**ZINC00520032**	ago	-7.691591	-8.436883
**ZINC00520224**	ago	-7.542856	-4.608997
**ZINC00520762**	ago	-4.845599	-5.643752
**ZINC00523239**	ago	-6.023912	-6.598138
**ZINC00523352**	ago	-6.803951	-6.726267
**ZINC00523596**	ago	-8.650682	-7.856559
**ZINC00523596**	ago	-8.287509	-5.770048
**ZINC00523596**	ago	-8.287509	-5.770048
**ZINC00523597**	ago	-8.668837	-7.501769
**ZINC00523597**	ago	-8.668837	-5.52999
**ZINC00523597**	ago	-8.29978	-5.52999
**ZINC00524370**	ago	-7.390511	-7.477365
**ZINC00524371**	ago		-6.656132
**ZINC00527709**	ago	-7.661128	-7.403127
**ZINC00527767**	ago	-6.94983	-7.285626
**ZINC00528832**	ago	-7.236833	-6.883768
**ZINC00528897**	ago	-7.203542	-6.840935
**ZINC00533817**	ago	-7.823779	-8.187163

**ZINC00534256**	ago	-7.74393	-7.819167
**ZINC00534618**	ago	-8.122652	-6.945616
**ZINC00534925**	ago	-7.663187	-7.520938
**ZINC00536277**	ago	-7.712119	-7.246238
**ZINC00536291**	ago	-7.924163	-7.405781
**ZINC00536292**	ago	-7.888578	-7.612385
**ZINC00536542**	ago	-7.780612	-5.738435
**ZINC00537955**	ago	-6.817402	-6.395165
**ZINC00538311**	ago	-6.482499	-6.299455
**ZINC00538840**	ago	-7.056245	-6.144522
**ZINC00538861**	ago	-6.969596	-6.129823
**ZINC00539308**	ago	-6.842173	-7.231699
**ZINC00539994**	ago	-6.860221	-5.697704
**ZINC00539994**	ago	-6.860221	-5.436136
**ZINC00539994**	ago	-6.578042	-5.436136
**ZINC00541301**	ago	-6.653969	-5.959263
**ZINC00542835**	ago	-6.31906	-6.137807
**ZINC00542920**	ago	-7.368859	-6.239629
**ZINC00543192**	ago	-6.946759	-6.547826
**ZINC00543381**	ago	-6.290447	-5.821025
**ZINC00543381**	ago	-6.154992	-5.677152
**ZINC00543381**	ago	-5.691203	-5.631628
**ZINC00543767**	ago	-7.650396	-4.288317
**ZINC00544238**	ago	-8.021397	-5.519542
**ZINC00544238**	ago	-7.881382	-5.51572

**ZINC00544530**	ago	-7.261394	-6.364881
**ZINC00546444**	ago	-5.514956	-6.637801
**ZINC00549762**	ago	-7.017317	-6.742479
**ZINC00550351**	ago	-5.668715	-6.135655
**ZINC00551476**	ago	-5.825535	-6.308311
**ZINC00554965**	ago	-7.828213	-7.798566
**ZINC00555999**	ago	-5.82315	-4.455778
**ZINC00556000**	ago	-6.799205	-4.049618
**ZINC00556001**	ago	-6.793444	-5.384883
**ZINC00556002**	ago	-7.257916	-5.815671
**ZINC00556007**	ago	-6.826576	-4.696664
**ZINC00556008**	ago	-6.099436	-5.293093
**ZINC00556009**	ago	-7.203988	-4.890958
**ZINC00556010**	ago	-7.372238	-5.544595
**ZINC00556030**	ago	-6.256305	-4.946458
**ZINC00556031**	ago	-5.936259	-5.129146
**ZINC00556032**	ago	-7.078772	-4.920864
**ZINC00556033**	ago	-7.204605	-5.079533
**ZINC00558003**	ago	-6.320044	-5.476245
**ZINC00558005**	ago	-6.196001	-5.499992
**ZINC00558661**	ago		-3.624579
**ZINC00561163**	ago	-8.41508	-7.412475
**ZINC00561361**	ago	-8.339116	-8.573398
**ZINC00561810**	ago	-7.986458	-6.217644
**ZINC00561955**	ago	-7.400844	-6.87028

**ZINC00562947**	ago	-8.594648	-7.33497
**ZINC00562967**	ago	-8.380039	-6.524254
**ZINC00562973**	ago	-7.584075	-3.089262
**ZINC00563049**	ago	-7.360934	-7.183243
**ZINC00563784**	ago	-6.280849	-5.34633
**ZINC00565333**	ago	-7.677097	-3.953084
**ZINC00565335**	ago	-7.420684	-4.483507
**ZINC00565630**	ago	-6.964191	-5.069459
**ZINC00565631**	ago	-6.731607	-5.959084
**ZINC00565638**	ago	-6.609977	-4.256725
**ZINC00565639**	ago	-7.49957	-4.063768
**ZINC00565640**	ago	-7.376385	-5.268618
**ZINC00565650**	ago	-6.706548	-4.618058
**ZINC00565651**	ago	-6.433708	-5.44801
**ZINC00565682**	ago	-6.457346	-5.256539
**ZINC00565683**	ago	-7.934815	-5.023443
**ZINC00565700**	ago	-6.596509	-4.585502
**ZINC00565704**	ago	-6.814374	-5.100805
**ZINC00565705**	ago	-6.972481	-5.877061
**ZINC00565715**	ago	-6.312617	-6.091608
**ZINC00565716**	ago	-6.573161	-4.998687
**ZINC00565717**	ago	-7.45116	-4.760068
**ZINC00565722**	ago	-6.027453	-4.170223
**ZINC00565723**	ago	-6.96322	-5.44877
**ZINC00565733**	ago	-6.997706	-5.582183

**ZINC00565735**	ago	-6.178348	-5.103222
**ZINC00565745**	ago	-6.963017	-4.996141
**ZINC00565750**	ago	-7.313813	-5.190005
**ZINC00566642**	ago	-6.861669	-5.041066
**ZINC00568773**	ago	-7.243264	-4.874831
**ZINC00569081**	ago	-7.301655	-4.847772
**ZINC00571929**	ago	-8.251719	-8.472914
**ZINC00572493**	ago	-8.378712	-8.156654
**ZINC00572493**	ago	-7.668689	-7.957618
**ZINC00572494**	ago	-8.427194	-8.134403
**ZINC00572494**	ago	-7.772621	-7.551917
**ZINC00573338**	ago	-6.953222	-5.448377
**ZINC00573451**	ago	-7.464012	-4.255835
**ZINC00574856**	ago	-8.332146	-4.825932
**ZINC00575206**	ago	-7.75778	-7.411659
**ZINC00575206**	ago	-7.595172	-7.145569
**ZINC00575206**	ago	-7.111498	-6.944853
**ZINC00575206**	ago	-7.108704	-5.6572
**ZINC00575575**	ago	-7.380913	-7.709531
**ZINC00578005**	ago	-6.719963	-5.880741
**ZINC00579979**	ago	-5.87205	-5.510914
**ZINC00580091**	ago	-6.386245	-6.075346
**ZINC00580369**	ago	-7.172529	-5.430598
**ZINC00580874**	ago	-7.364948	-6.471095
**ZINC00580984**	ago	-8.176708	-6.362257

**ZINC00581215**	ago	-7.609923	-6.075939
**ZINC00583021**	ago	-7.752646	-5.695298
**ZINC00583042**	ago	-8.30497	-7.685373
**ZINC00583790**	ago	-6.402516	-5.680664
**ZINC00586211**	ago	-6.746142	-5.561218
**ZINC00586216**	ago	-6.180377	-4.922665
**ZINC00588445**	ago	-5.854668	-5.825334
**ZINC00596101**	ago	-7.664754	-7.261985
**ZINC00608581**	ago	-8.804721	-8.720674
**ZINC00610972**	ago	-6.979641	-6.796992
**ZINC00611491**	ago	-8.05374	-5.70872
**ZINC00611730**	ago	-4.338764	-4.541297
**ZINC00613470**	ago	-6.630549	-5.235645
**ZINC00613887**	ago	-6.461451	-6.646903
**ZINC00614468**	ago	-6.204839	-5.77302
**ZINC00618816**	ago	-7.295348	-7.395534
**ZINC00620798**	ago	-8.261086	-8.030157
**ZINC00621386**	ago	-7.979588	-7.301246
**ZINC00621387**	ago	-8.231394	-7.370133
**ZINC00621552**	ago	-6.332118	-5.257813
**ZINC00809344**	ago	-6.634674	-6.052411
**ZINC00817982**	ago	-7.020693	-6.770114
**ZINC00831904**	ago	-5.682273	-7.114389
**ZINC00858539**	ago	-5.943898	-5.272123
**ZINC00868863**	ago	-6.590553	-6.199265

**ZINC00868864**	ago	-6.830022	-6.191494
**ZINC00898027**	ago	-8.067246	-7.778304
**ZINC00903783**	ago	-7.906465	-6.143645
**ZINC00935709**	ago	-8.174928	-6.99927
**ZINC00935709**	ago	-8.174928	-6.99927
**ZINC00935709**	ago	-7.327628	-6.1053
**ZINC00968262**	ago	-7.094681	-6.378102
**ZINC00974831**	ago		-4.057795
**ZINC00974832**	ago		-3.86245
**ZINC00983278**	ago	-7.598791	-8.158754
**ZINC00985077**	ago	-8.44762	-5.77042
**ZINC00991276**	ago	-6.830142	-5.697454
**ZINC00994227**	ago	-7.253642	-6.641453
**ZINC01016455**	ago	-7.09555	-6.660912
**ZINC01017493**	ago	-5.972557	-7.637025
**ZINC01027997**	ago	-7.440527	-8.167039
**ZINC01036277**	ago	-7.649109	-7.878294
**ZINC01040987**	ago	-8.427952	-7.40786
**ZINC01040988**	ago	-7.374528	-7.906673
**ZINC01041069**	ago	-8.111711	-7.375862
**ZINC01041069**	ago	-7.646447	-4.147898
**ZINC01041070**	ago	-7.965431	-7.944561
**ZINC01041070**	ago	-7.916648	-7.519147
**ZINC01041207**	ago	-8.203567	-7.215867
**ZINC01042169**	ago	-7.908997	-1.407058

**ZINC01042172**	ago	-7.26381	-4.787983
**ZINC01052190**	ago	-5.77007	-3.820208
**ZINC01052591**	ago	-6.628152	-6.798972
**ZINC01053687**	ago	-6.409	-6.470075
**ZINC01054102**	ago	-6.854089	-4.614752
**ZINC01054990**	ago	-7.421567	-6.272724
**ZINC01055022**	ago	-6.123166	-6.676683
**ZINC01055040**	ago	-6.312655	-7.14069
**ZINC01056452**	ago	-5.874116	-6.452814
**ZINC01069200**	ago	-7.619481	-7.373592
**ZINC01070713**	ago	-7.146871	-4.900468
**ZINC01076057**	ago	-5.987918	-6.593121
**ZINC01077308**	ago	-6.207216	-5.645886
**ZINC01077309**	ago	-7.833644	-5.93949
**ZINC01094836**	ago	-8.087728	-7.919223
**ZINC01100919**	ago	-6.23211	-6.551028
**ZINC01100920**	ago	-6.111631	-6.357694
**ZINC01126443**	ago	-7.17715	-7.066842
**ZINC01140399**	ago	-6.853415	-5.142615
**ZINC01150238**	ago		-4.713647
**ZINC01181126**	ago	-7.59825	-4.975116
**ZINC01196454**	ago	-6.540199	-6.069316
**ZINC01247845**	ago	-7.813514	-7.620961
**ZINC01252115**	ago	-6.863172	-6.371061
**ZINC01269839**	ago	-7.888422	-7.161418

**ZINC01275347**	ago	-7.355132	-7.451422
**ZINC01279320**	ago	-6.980753	-6.100676
**ZINC01279623**	ago	-8.060927	-7.20292
**ZINC01280904**	ago	-8.064091	-8.085497
**ZINC01280904**	ago	-8.064091	-8.085497
**ZINC01280904**	ago	-7.942051	-7.253574
**ZINC01280939**	ago	-8.137669	-7.535944
**ZINC01280939**	ago	-8.137669	-7.535944
**ZINC01280939**	ago	-7.262559	-5.712448
**ZINC01281434**	ago	-7.404032	-6.812156
**ZINC01281434**	ago	-7.318184	-6.812156
**ZINC01281434**	ago	-7.238347	-6.036259
**ZINC01281434**	ago	-7.238347	-5.511966
**ZINC01281436**	ago	-7.963083	-8.209576
**ZINC01281436**	ago	-7.963083	-8.209576
**ZINC01281436**	ago	-7.040271	-6.811395
**ZINC01281436**	ago	-6.728312	-6.5253
**ZINC01281437**	ago	-8.146047	-8.176875
**ZINC01281437**	ago	-8.146047	-8.176875
**ZINC01281437**	ago	-6.958469	-7.36144
**ZINC01281437**	ago	-6.69377	-6.856157
**ZINC01281445**	ago	-7.763459	-6.16831
**ZINC01281445**	ago	-7.763459	-6.16831
**ZINC01281445**	ago	-7.539228	-5.919462
**ZINC01291411**	ago	-7.870436	-7.803103

**ZINC01291412**	ago	-8.666246	-8.025611
**ZINC01292227**	ago	-5.904036	-5.427385
**ZINC01312064**	ago	-7.299983	-6.152201
**ZINC01318904**	ago	-6.721496	-7.294158
**ZINC01319998**	ago	-7.611803	-7.044243
**ZINC01320283**	ago	-7.971015	-7.362881
**ZINC01325325**	ago	-6.433011	-6.902333
**ZINC01366260**	ago	-5.467185	-6.295146
**ZINC01384938**	ago	-7.754627	-5.072525
**ZINC01384954**	ago	-5.865351	-7.106082
**ZINC01384954**	ago	-5.795952	-6.10642
**ZINC01394939**	ago	-6.682349	-7.372838
**ZINC01398321**	ago	-8.158371	-7.539866
**ZINC01398627**	ago	-6.820552	-6.218196
**ZINC01398629**	ago	-6.809884	-6.182821
**ZINC01399032**	ago	-8.555766	-7.690789
**ZINC01399032**	ago	-8.555766	-7.690789
**ZINC01399032**	ago	-7.082647	-5.793265
**ZINC01399719**	ago	-7.700868	-7.116475
**ZINC01402582**	ago	-6.667816	-6.208423
**ZINC01404530**	ago	-6.92896	-4.92378
**ZINC01410002**	ago	-8.044141	-7.856303
**ZINC01410003**	ago	-8.727015	-6.705174
**ZINC01410464**	ago	-7.708011	-7.764797
**ZINC01420471**	ago	-5.766633	-5.199478

**ZINC01420471**	ago	-4.996203	-5.174838
**ZINC01422182**	ago	-7.644139	-5.515435
**ZINC01422183**	ago	-6.89622	-4.774552
**ZINC01428555**	ago	-6.5976	-6.510596
**ZINC01428555**	ago	-6.5976	-6.423344
**ZINC01428555**	ago	-6.482868	-6.423344
**ZINC01433169**	ago	-6.664785	-6.325127
**ZINC01446484**	ago	-7.202855	-6.205232
**ZINC01446484**	ago	-6.931009	-6.063031
**ZINC01446485**	ago	-5.87527	-6.497886
**ZINC01446485**	ago	-5.801165	-6.253664
**ZINC01446486**	ago	-5.942744	-5.957307
**ZINC01446486**	ago	-5.879341	-5.819562
**ZINC01446487**	ago	-6.925456	-6.622333
**ZINC01446487**	ago	-5.360368	-6.460033
**ZINC01456181**	ago	-6.85831	-5.758208
**ZINC01456701**	ago	-6.398709	-5.72711
**ZINC01459881**	ago	-7.013933	-7.940696
**ZINC01459882**	ago	-7.079281	-7.989238
**ZINC01464919**	ago	-6.845055	-4.241283
**ZINC01470617**	ago	-7.72818	-7.175259
**ZINC01470617**	ago	-7.430312	-6.692131
**ZINC01472305**	ago	-7.031238	-5.415344
**ZINC01476740**	ago	-7.391501	-5.862094
**ZINC01495311**	ago	-7.600414	-7.017744

**ZINC01500903**	ago	-7.195286	-7.714602
**ZINC01501442**	ago	-6.768878	-7.805261
**ZINC01504755**	ago	-5.104495	-6.891774
**ZINC01504755**	ago	-7.822876	-6.55474
**ZINC01506209**	ago	-8.130335	-6.198663
**ZINC01507653**	ago	-7.166777	-6.456707
**ZINC01507835**	ago		-5.894449
**ZINC01513590**	ago	-7.423873	-6.546488
**ZINC01514781**	ago	-8.97784	-6.430098
**ZINC01516515**	ago	-8.332241	-7.775113
**ZINC01518999**	ago	-5.9622	-6.159207
**ZINC01519000**	ago	-7.600576	-6.963943
**ZINC01536668**	ago	-6.707844	-5.571478
**ZINC01536948**	ago	-6.677479	-5.972528
**ZINC01542529**	ago	-7.576911	-6.105933
**ZINC01542777**	ago	-8.532284	-7.237285
**ZINC01554458**	ago	-6.003141	-5.705792
**ZINC01569660**	ago	-8.568504	-8.144754
**ZINC01575059**	ago	-8.619911	-4.494557
**ZINC01576843**	ago	-7.56043	-4.830927
**ZINC01580673**	ago		
**ZINC01580906**	ago	-8.799085	-7.556105
**ZINC01585780**	ago	-6.991377	-7.806084
**ZINC01586846**	ago	-7.817372	-7.433283
**ZINC01595661**	ago	-6.593093	-6.371836

**ZINC01598745**	ago	-7.773411	-7.054908
**ZINC01612041**	ago	-7.597186	-6.62567
**ZINC01612041**	ago	-7.019187	-6.572259
**ZINC01613570**	ago	-6.155544	-6.264283
**ZINC01616861**	ago	-8.070505	-8.489351
**ZINC01620100**	ago	-7.450209	-6.490294
**ZINC01620100**	ago	-7.261715	-6.32352
**ZINC01620388**	ago	-7.548446	-6.472052
**ZINC01621240**	ago	-6.140782	-6.906411
**ZINC01626331**	ago	-7.534272	-8.27172
**ZINC01627515**	ago	-7.181856	-6.409028
**ZINC01629654**	ago	-7.340863	-6.868078
**ZINC01630593**	ago	-6.639388	-6.764386
**ZINC01636366**	ago	-7.02453	-4.797953
**ZINC01639322**	ago	-8.722276	-3.927342
**ZINC01647451**	ago	-6.242483	-6.741695
**ZINC01650980**	ago	-7.661427	-7.656406
**ZINC01650980**	ago	-7.634726	-6.573484
**ZINC01650989**	ago	-7.224831	-7.239352
**ZINC01650989**	ago	-6.698307	-6.855074
**ZINC01654061**	ago	-7.400319	-4.507345
**ZINC01658350**	ago	-8.18185	-6.684992
**ZINC01663678**	ago	-6.602898	-7.8238
**ZINC01663679**	ago	-8.529344	-5.014987
**ZINC01666289**	ago	-9.301155	-7.793647

**ZINC01666820**	ago	-8.130955	-8.098226
**ZINC01670243**	ago	-7.73783	-7.467203
**ZINC01676880**	ago	-6.907993	-7.063688
**ZINC01677173**	ago	-8.200773	-7.470118
**ZINC01678820**	ago	-7.042931	-7.684082
**ZINC01682044**	ago	-7.05962	-6.310351
**ZINC01683226**	ago	-7.255618	-6.565567
**ZINC01683226**	ago	-7.069167	-5.706757
**ZINC01690791**	ago	-9.315029	-9.183421
**ZINC01696129**	ago	-7.548333	-7.851231
**ZINC01699583**	ago	-7.813686	-6.769564
**ZINC01704165**	ago	-7.492076	-6.594354
**ZINC01708605**	ago	-6.968044	-6.039321
**ZINC01716556**	ago	-7.200352	-5.857882
**ZINC01739317**	ago	-6.512587	-5.293731
**ZINC01742864**	ago	-5.357569	-6.426067
**ZINC01744290**	ago	-6.625715	-6.570165
**ZINC01754793**	ago	-7.394695	-5.982454
**ZINC01757351**	ago	-7.704595	-4.358566
**ZINC01792360**	ago	-7.121323	-5.699975
**ZINC01815001**	ago	-6.193676	-6.372524
**ZINC01840706**	ago	-7.189844	-7.107249
**ZINC01857883**	ago	-7.170758	-6.034965
**ZINC01859746**	ago	-6.612381	-6.390479
**ZINC01876582**	ago	-8.329169	-6.808627

**ZINC01880250**	ago	-6.390312	-5.898609
**ZINC01881588**	ago	-6.613612	-5.834032
**ZINC01883728**	ago	-8.074475	-8.21853
**ZINC01883733**	ago	-7.85236	-7.37624
**ZINC01911903**	ago	-6.165606	-7.785794
**ZINC01922532**	ago	-7.69971	-7.246986
**ZINC01922552**	ago	-7.302835	-7.277185
**ZINC01924071**	ago	-6.964674	-5.895947
**ZINC01924075**	ago	-7.250095	-6.372157
**ZINC01961565**	ago	-6.837143	-4.78859
**ZINC01961566**	ago	-7.024599	-5.324657
**ZINC01993830**	ago	-6.890575	-6.062956
**ZINC01993844**	ago	-6.23313	-6.033487
**ZINC01998241**	ago	-7.722334	-7.217729
**ZINC02009400**	ago	-6.175693	-6.898182
**ZINC02022163**	ago	-7.953569	-6.648375
**ZINC02022863**	ago	-6.190365	-6.140803
**ZINC02023791**	ago	-6.283508	-5.725083
**ZINC02026515**	ago	-6.481206	-5.566663
**ZINC02026520**	ago	-6.405919	-5.410369
**ZINC02035552**	ago	-7.63889	-7.109225
**ZINC02079715**	ago	-7.225725	-7.158277
**ZINC02079717**	ago	-6.475422	-7.226105
**ZINC02099412**	ago		-6.67059
**ZINC02104894**	ago	-7.479695	-8.149177

**ZINC02130508**	ago	-6.844267	-6.695081
**ZINC02130571**	ago	-7.660869	-7.914388
**ZINC02131344**	ago	-8.38748	-7.472671
**ZINC02134976**	ago		-2.204514
**ZINC02136055**	ago	-6.782297	-6.058661
**ZINC02136356**	ago	-8.579734	-2.042697
**ZINC02146356**	ago		-6.872785
**ZINC02146360**	ago	-8.199251	-6.041626
**ZINC02148553**	ago	-6.120726	-5.79013
**ZINC02156127**	ago	-8.147922	-5.79158
**ZINC02157878**	ago	-6.06307	-5.033006
**ZINC02159149**	ago	-7.150247	-5.675792
**ZINC02161451**	ago	-5.801725	-2.141154
**ZINC02161454**	ago	-4.385269	-1.728791
**ZINC02162373**	ago	-6.943013	-5.577164
**ZINC02169638**	ago	-9.445213	-7.669621
**ZINC02171329**	ago	-7.12011	-5.136718
**ZINC02173032**	ago	-7.482472	-6.525821
**ZINC02179591**	ago	-7.167052	-6.847924
**ZINC02179592**	ago	-6.480809	-4.847794
**ZINC02200057**	ago	-7.312093	-6.292336
**ZINC02202278**	ago	-7.688377	-5.896373
**ZINC02210173**	ago	-6.678884	-5.729756
**ZINC02213867**	ago	-7.085984	-6.633892
**ZINC02217534**	ago	-6.822349	-5.269275

**ZINC02223055**	ago	-7.035763	-7.123859
**ZINC02223200**	ago	-6.495415	-6.808974
**ZINC02231288**	ago	-7.182024	-6.742398
**ZINC02236712**	ago	-7.201304	-6.714148
**ZINC02263982**	ago	-7.258291	-5.749971
**ZINC02267756**	ago	-7.213481	-6.206513
**ZINC02273184**	ago	-6.915409	-5.959713
**ZINC02292290**	ago	-7.523685	-7.314159
**ZINC02294557**	ago	-7.348532	-7.069038
**ZINC02294558**	ago	-5.987966	-7.11982
**ZINC02306420**	ago	-8.627163	-6.534112
**ZINC02315488**	ago	-4.786434	-4.075624
**ZINC02327096**	ago	-7.654084	-6.496065
**ZINC02327583**	ago	-6.899816	-6.247669
**ZINC02330094**	ago	-6.858472	-6.175159
**ZINC02330112**	ago	-7.501814	-6.968324
**ZINC02333569**	ago	-7.093788	-5.842175
**ZINC02336994**	ago	-8.099386	-6.803202
**ZINC02342321**	ago	-7.27415	-6.625637
**ZINC02342321**	ago	-7.27415	-6.094943
**ZINC02342321**	ago	-7.2465	-6.094943
**ZINC02342321**	ago	-6.899569	-5.719837
**ZINC02342407**	ago	-7.465705	-7.250214
**ZINC02345165**	ago	-4.439015	-6.612289
**ZINC02347439**	ago	-6.583335	-6.320247

**ZINC02354761**	ago	-6.830905	-6.110923
**ZINC02359546**	ago	-6.703866	-6.10102
**ZINC02360726**	ago	-7.98917	-7.534486
**ZINC02360727**	ago	-8.425812	-7.6038
**ZINC02362916**	ago	-7.692223	-4.846651
**ZINC02364744**	ago	-6.828564	-6.412864
**ZINC02385470**	ago	-6.765419	-7.122201
**ZINC02387600**	ago	-7.17679	-5.776545
**ZINC02400223**	ago	-6.398742	-5.464668
**ZINC02419495**	ago	-7.989249	-4.599058
**ZINC02419496**	ago	-7.980151	-7.489713
**ZINC02423385**	ago	-7.666094	-6.945237
**ZINC02431617**	ago	-6.83463	-5.803295
**ZINC02432839**	ago	-7.503928	-6.282423
**ZINC02434806**	ago	-6.561595	-5.94107
**ZINC02435371**	ago	-7.013277	-6.343506
**ZINC02438608**	ago	-6.342053	-6.667924
**ZINC02439757**	ago	-6.603119	-7.786839
**ZINC02452061**	ago	-7.44226	-4.46295
**ZINC02452858**	ago	-7.773245	-6.588985
**ZINC02452888**	ago	-7.564984	-6.419246
**ZINC02453043**	ago	-8.459973	-7.773648
**ZINC02453724**	ago	-7.336252	-5.968351
**ZINC02455152**	ago	-7.551696	-4.116078
**ZINC02464434**	ago	-8.03044	-5.682854

**ZINC02464572**	ago	-6.280779	-4.653072
**ZINC02469681**	ago	-8.038925	-4.888443
**ZINC02473887**	ago	-8.756413	-8.541022
**ZINC02474999**	ago	-7.614246	-4.386238
**ZINC02481049**	ago	-8.512148	-5.357525
**ZINC02481994**	ago	-5.383728	-5.942682
**ZINC02482877**	ago	-7.028612	-4.228927
**ZINC02484772**	ago	-7.43124	-4.552388
**ZINC02490261**	ago	-7.320266	-7.189459
**ZINC02490270**	ago	-8.289177	-5.34784
**ZINC02496572**	ago	-7.533796	-6.519593
**ZINC02502234**	ago	-8.273966	-5.707051
**ZINC02502582**	ago	-7.787584	-5.429818
**ZINC02504446**	ago	-7.488523	-6.493376
**ZINC02506006**	ago	-6.546344	-4.075888
**ZINC02506009**	ago	-6.801563	-4.111016
**ZINC02509821**	ago	-7.665899	-6.85928
**ZINC02510611**	ago	-7.501656	-6.827191
**ZINC02512657**	ago	-8.897214	-8.486787
**ZINC02518248**	ago	-6.54789	-6.362606
**ZINC02518947**	ago	-6.933221	-6.647481
**ZINC02524319**	ago	-7.211104	-6.818609
**ZINC02524786**	ago	-7.728504	-7.523526
**ZINC02527121**	ago	-6.817909	-6.876434
**ZINC02528970**	ago	-8.054986	-6.287106

**ZINC02529411**	ago	-8.032907	-7.599023
**ZINC02534829**	ago	-6.907891	-6.610844
**ZINC02534843**	ago	-7.162818	-7.230449
**ZINC02534899**	ago	-7.279331	-6.087019
**ZINC02534916**	ago	-7.601848	-7.446973
**ZINC02534941**	ago	-6.589525	-5.760967
**ZINC02534952**	ago	-8.072259	-6.889
**ZINC02534955**	ago	-5.946762	-6.145409
**ZINC02534956**	ago	-6.555686	-5.751191
**ZINC02534972**	ago	-7.506151	-7.413444
**ZINC02535329**	ago	-8.834687	-6.901332
**ZINC02535865**	ago	-7.682709	-5.640803
**ZINC02541427**	ago	-6.528343	-6.478126
**ZINC02541465**	ago	-7.147644	-5.719792
**ZINC02543369**	ago	-7.591039	-6.641856
**ZINC02543468**	ago	-8.000273	-4.6496
**ZINC02545374**	ago	-6.741188	-7.018405
**ZINC02546668**	ago	-8.297429	-8.261676
**ZINC02549466**	ago	-7.327736	-4.18219
**ZINC02550132**	ago	-7.732585	-6.976303
**ZINC02550491**	ago	-7.017732	-4.349616
**ZINC02558495**	ago	-8.296066	-6.602983
**ZINC02558500**	ago	-7.869776	-5.961964
**ZINC02558512**	ago	-6.980143	-7.007423
**ZINC02558527**	ago	-7.000755	-5.143149

**ZINC02558529**	ago	-7.146921	-6.411166
**ZINC02558530**	ago	-6.814206	-7.121571
**ZINC02558531**	ago	-7.125343	-6.775722
**ZINC02561669**	ago	-8.11989	-7.896764
**ZINC02561986**	ago		-6.003309
**ZINC02562057**	ago	-7.247395	-5.928699
**ZINC02567739**	ago	-6.419614	-6.467245
**ZINC02569321**	ago	-7.673515	-6.786842
**ZINC02573410**	ago	-7.931666	-5.214488
**ZINC02573673**	ago	-9.050268	-7.315557
**ZINC02575559**	ago	-6.185447	-7.009392
**ZINC02576980**	ago	-7.914659	-7.261847
**ZINC02578733**	ago	-8.411074	-7.201127
**ZINC02580949**	ago	-7.503162	-7.440192
**ZINC02581494**	ago	-6.308212	-6.582817
**ZINC02584871**	ago	-7.731001	-7.296001
**ZINC02584877**	ago	-7.98474	-4.790365
**ZINC02584887**	ago	-7.495489	-7.738257
**ZINC02590937**	ago	-7.04533	-6.725797
**ZINC02593207**	ago	-7.899004	-8.212291
**ZINC02597429**	ago	-7.591134	-7.121747
**ZINC02597436**	ago	-8.196252	-4.585071
**ZINC02606577**	ago	-8.272141	-5.821861
**ZINC02627803**	ago	-7.319421	-3.819959
**ZINC02628036**	ago	-8.419567	-6.113298

**ZINC02628040**	ago	-6.824699	-6.011793
**ZINC02630798**	ago	-8.102607	-7.844519
**ZINC02641117**	ago	-7.981491	-6.168628
**ZINC02645085**	ago	-8.842034	-7.159752
**ZINC02645358**	ago	-7.127298	-6.935472
**ZINC02650600**	ago	-6.970179	-6.490776
**ZINC02651581**	ago	-7.203695	-5.733063
**ZINC02659985**	ago	-8.183713	-7.450848
**ZINC02660376**	ago	-6.639998	-5.906127
**ZINC02665136**	ago	-5.859287	-6.285311
**ZINC02685704**	ago	-7.185774	-6.22338
**ZINC02685706**	ago	-6.942274	-5.644281
**ZINC02685796**	ago	-6.619386	-5.96419
**ZINC02689941**	ago	-6.498537	-3.561624
**ZINC02689943**	ago	-7.817969	-7.239734
**ZINC02701585**	ago	-8.313807	-7.533905
**ZINC02720594**	ago	-7.975739	-7.091398
**ZINC02720595**	ago	-7.376809	-6.442268
**ZINC02720596**	ago	-7.995377	-6.697891
**ZINC02729603**	ago	-5.801193	-5.816667
**ZINC02730552**	ago	-7.132259	-5.451052
**ZINC02730553**	ago	-6.489841	-5.884909
**ZINC02737933**	ago	-5.919288	-6.751987
**ZINC02737934**	ago	-7.190373	-5.406754
**ZINC02737954**	ago	-7.455609	-5.937433

**ZINC02738012**	ago	-7.340864	-6.491928
**ZINC02738013**	ago	-7.011143	-5.832799
**ZINC02739080**	ago	-7.233313	-5.71826
**ZINC02741472**	ago	-8.271991	-4.616112
**ZINC02742536**	ago	-6.837276	-6.104241
**ZINC02743869**	ago	-6.77638	-5.855584
**ZINC02746880**	ago	-7.432941	-5.812851
**ZINC02748690**	ago	-7.997137	-5.516652
**ZINC02750522**	ago	-6.825558	-6.121162
**ZINC02750523**	ago	-7.18111	-5.318043
**ZINC02753858**	ago	-6.836189	-4.74245
**ZINC02753859**	ago	-6.695006	-5.384629
**ZINC02754011**	ago	-6.598284	-5.562368
**ZINC02755323**	ago	-6.547515	-7.089454
**ZINC02755350**	ago	-6.563818	-5.341809
**ZINC02760792**	ago	-6.769648	-6.040637
**ZINC02763750**	ago	-7.677972	-6.974924
**ZINC02764567**	ago	-6.678428	-5.761633
**ZINC02767146**	ago	-6.272409	-4.278221
**ZINC02777395**	ago	-7.55724	-5.381342
**ZINC02777400**	ago	-6.50576	-6.107566
**ZINC02782960**	ago	-6.672692	-6.540763
**ZINC02783103**	ago	-6.557959	-5.425314
**ZINC02785278**	ago	-7.828144	-6.006777
**ZINC02789187**	ago	-6.410346	-7.217419

**ZINC02790570**	ago	-6.084114	-5.950908
**ZINC02790608**	ago	-7.156385	-5.435791
**ZINC02790609**	ago	-7.192644	-5.369192
**ZINC02790623**	ago	-7.857364	-6.677093
**ZINC02790737**	ago	-7.065627	-5.736982
**ZINC02796081**	ago	-6.569242	-6.813303
**ZINC02806599**	ago		
**ZINC02811176**	ago	-6.480315	-5.440276
**ZINC02813806**	ago	-6.967625	-5.204591
**ZINC02818370**	ago	-6.945616	-5.129409
**ZINC02819819**	ago	-8.437341	-5.247926
**ZINC02833281**	ago		-5.254816
**ZINC02843193**	ago	-7.311001	-8.375803
**ZINC02843196**	ago	-7.579341	-8.14946
**ZINC02854968**	ago	-7.670663	-7.603636
**ZINC02865841**	ago	-8.687425	-8.497942
**ZINC02875050**	ago	-6.905958	-7.418328
**ZINC02877819**	ago	-7.631295	-7.041365
**ZINC02879866**	ago	-8.139184	-7.087427
**ZINC02883628**	ago	-7.276849	-4.135781
**ZINC02887707**	ago	-6.203073	-6.283094
**ZINC02889052**	ago	-7.159422	-7.679304
**ZINC02891262**	ago	-6.631444	-5.223347
**ZINC02891271**	ago	-6.085173	-5.746491
**ZINC02892001**	ago	-7.117815	-5.239524

**ZINC02903255**	ago	-5.695706	-6.319758
**ZINC02904129**	ago		-5.727164
**ZINC02916128**	ago	-7.52898	-7.205557
**ZINC02916614**	ago	-7.827095	-7.310913
**ZINC02916615**	ago	-7.861655	-7.239673
**ZINC02918209**	ago	-7.812944	-7.674727
**ZINC02939083**	ago	-6.441755	-6.718923
**ZINC02950611**	ago	-5.624731	-7.218848
**ZINC02999401**	ago	-7.824034	-8.852261
**ZINC03016439**	ago	-7.096524	-5.749375
**ZINC03030641**	ago	-8.534559	-6.698302
**ZINC03030641**	ago	-8.534559	-6.698302
**ZINC03030641**	ago	-7.751276	-6.076345
**ZINC03033062**	ago	-7.097843	-5.844746
**ZINC03043061**	ago	-8.050375	-7.456818
**ZINC03043069**	ago	-9.094122	-7.759673
**ZINC03057133**	ago	-7.784958	-5.171741
**ZINC03061497**	ago	-7.071677	-6.635852
**ZINC03070606**	ago	-6.839515	
**ZINC03073069**	ago	-7.057215	-5.408927
**ZINC03075209**	ago	-7.033016	-5.45627
**ZINC03075213**	ago	-7.605599	-5.509752
**ZINC03078614**	ago	-6.106505	-6.039245
**ZINC03084404**	ago	-7.174715	-5.905747
**ZINC03093817**	ago	-7.344271	-6.556666

**ZINC03101851**	ago	-7.05039	-5.773663
**ZINC03102059**	ago	-4.933604	-5.627768
**ZINC03110836**	ago	-6.982656	-4.533393
**ZINC03124536**	ago	-5.845606	-6.198628
**ZINC03145349**	ago	-6.3334	-4.8965
**ZINC03151515**	ago	-6.889885	-7.1604
**ZINC03152757**	ago	-7.504414	-6.075112
**ZINC03156920**	ago	-7.584179	-5.077234
**ZINC03157856**	ago	-7.37066	-7.46685
**ZINC03158100**	ago	-6.803203	-5.708215
**ZINC03164452**	ago	-6.762111	-6.603775
**ZINC03165963**	ago	-7.029167	-4.068551
**ZINC03166448**	ago	-8.315873	-7.623337
**ZINC03166450**	ago	-8.975825	-7.546137
**ZINC03178797**	ago	-7.763138	-6.382618
**ZINC03182193**	ago	-6.536494	-6.41486
**ZINC03182484**	ago		-5.529206
**ZINC03185136**	ago	-6.840671	-5.635486
**ZINC03185350**	ago	-6.506938	-6.068981
**ZINC03188821**	ago	-6.271901	-6.962438
**ZINC03189156**	ago	-7.289819	-5.409674
**ZINC03194076**	ago	-8.178498	-6.041447
**ZINC03194246**	ago	-8.269569	-8.351046
**ZINC03194249**	ago	-8.280348	-7.513608
**ZINC03194363**	ago	-6.645082	-5.288011

**ZINC03196581**	ago	-7.890051	-7.855405
**ZINC03198588**	ago	-7.533276	-8.092359
**ZINC03198657**	ago	-7.70756	-8.188893
**ZINC03199353**	ago	-7.787771	-7.637114
**ZINC03199964**	ago	-8.092194	-7.011964
**ZINC03201406**	ago	-8.744379	-7.08905
**ZINC03201888**	ago	-6.937802	-7.150795
**ZINC03205476**	ago	-7.761131	-7.196341
**ZINC03209521**	ago	-8.011783	-7.589692
**ZINC03209699**	ago	-8.094738	-5.076579
**ZINC03213384**	ago	-6.310642	-6.419865
**ZINC03218569**	ago		-6.068138
**ZINC03220572**	ago	-8.661109	-4.453729
**ZINC03227931**	ago	-6.475302	-5.78172
**ZINC03230700**	ago	-8.300034	-7.22714
**ZINC03231603**	ago	-7.193475	-6.810481
**ZINC03233024**	ago	-6.80343	-6.496441
**ZINC03233025**	ago	-6.632299	-6.34884
**ZINC03233620**	ago	-6.523991	-5.535903
**ZINC03233621**	ago	-5.73938	-5.492929
**ZINC03233624**	ago	-7.16817	-5.920166
**ZINC03233625**	ago	-6.552842	-5.247372
**ZINC03233626**	ago		-5.884832
**ZINC03233630**	ago	-6.252427	-5.193295
**ZINC03233702**	ago	-5.97522	-5.909805

**ZINC03233703**	ago	-6.4568	-5.871381
**ZINC03236879**	ago	-8.223699	-7.278269
**ZINC03239531**	ago	-6.766772	-6.37714
**ZINC03239532**	ago	-6.580562	-6.367052
**ZINC03241272**	ago	-6.554502	-6.147162
**ZINC03241273**	ago	-7.026369	-6.399285
**ZINC03243823**	ago		-6.246964
**ZINC03243939**	ago	-6.448249	-6.873541
**ZINC03246319**	ago	-6.300902	-6.697729
**ZINC03249699**	ago	-6.436557	-5.619215
**ZINC03250408**	ago	-6.136451	-5.275669
**ZINC03250410**	ago	-5.866886	-5.387835
**ZINC03253049**	ago	-7.065776	-5.5705
**ZINC03253052**	ago	-7.157904	-5.742957
**ZINC03257738**	ago	-7.141731	-7.002998
**ZINC03257891**	ago	-6.88591	-7.289087
**ZINC03261355**	ago	-6.92403	-6.010155
**ZINC03262357**	ago	-6.791708	-7.39175
**ZINC03265816**	ago	-7.475004	-4.94386
**ZINC03265819**	ago	-7.210238	-5.298006
**ZINC03268059**	ago	-6.853046	-6.57389
**ZINC03268080**	ago	-7.254538	-7.46154
**ZINC03270427**	ago	-6.771398	-5.25032
**ZINC03270568**	ago	-5.982774	-5.855846
**ZINC03273246**	ago	-6.14721	-6.550802

**ZINC03274387**	ago	-7.751373	-7.865118
**ZINC03280829**	ago	-7.584687	-6.917827
**ZINC03288034**	ago	-6.840459	-5.587612
**ZINC03289680**	ago	-7.142641	-6.852706
**ZINC03292822**	ago		-7.46192
**ZINC03300932**	ago	-8.089799	-7.358598
**ZINC03304260**	ago	-6.91793	-6.329591
**ZINC03304263**	ago	-7.790871	-6.031756
**ZINC03305073**	ago	-6.665016	-6.265433
**ZINC03305074**	ago	-7.79348	-6.354345
**ZINC03305078**	ago	-7.77356	-5.165965
**ZINC03305469**	ago	-8.209255	-7.545323
**ZINC03311200**	ago	-7.278341	-5.220066
**ZINC03313643**	ago	-6.776148	-7.46114
**ZINC03313978**	ago	-6.131275	-5.695663
**ZINC03318814**	ago	-7.236777	-7.738635
**ZINC03319694**	ago	-6.4941	-6.163377
**ZINC03322345**	ago	-7.587643	-5.282937
**ZINC03329605**	ago	-7.46066	-5.322413
**ZINC03330631**	ago	-7.229629	-5.653153
**ZINC03333669**	ago	-5.62683	-7.427012
**ZINC03335616**	ago	-6.461334	-6.447605
**ZINC03340227**	ago	-6.301609	-5.757095
**ZINC03340262**	ago	-7.800542	-7.077454
**ZINC03343072**	ago		-5.645509

**ZINC03345294**	ago	-5.275857	-5.377838
**ZINC03349880**	ago	-7.269086	-6.386877
**ZINC03351615**	ago	-7.723426	-5.507333
**ZINC03351617**	ago	-7.3911	-5.99594
**ZINC03352358**	ago	-7.764286	-7.730204
**ZINC03352358**	ago	-7.764286	-7.730204
**ZINC03352363**	ago	-7.818529	-7.489758
**ZINC03352363**	ago	-7.818529	-7.489758
**ZINC03355399**	ago	-7.527999	-7.068889
**ZINC03355400**	ago	-7.8683	-6.896962
**ZINC03355401**	ago	-7.594994	-3.855271
**ZINC03355404**	ago	-7.52759	-3.6736
**ZINC03356061**	ago	-7.913921	-6.042804
**ZINC03362560**	ago	-6.447929	-6.334419
**ZINC03364389**	ago	-7.322131	-6.624469
**ZINC03372384**	ago	-7.353041	-5.099902
**ZINC03379538**	ago	-6.617091	-6.2858
**ZINC03382325**	ago	-7.397279	-5.800818
**ZINC03388867**	ago	-6.729218	-7.227523
**ZINC03394127**	ago	-6.281055	-6.387883
**ZINC03396406**	ago	-7.345527	-6.426828
**ZINC03396901**	ago	-7.769108	-3.701464
**ZINC03396904**	ago	-7.597299	-6.4104
**ZINC03396907**	ago	-7.904657	-4.110752
**ZINC03396910**	ago	-7.597387	-3.677051

**ZINC03418718**	ago	-7.71067	-7.76811
**ZINC03434681**	ago	-7.551368	-7.159387
**ZINC03440753**	ago	-7.044024	-7.101207
**ZINC03440755**	ago	-7.964052	-5.840559
**ZINC03446655**	ago	-4.994088	-5.806288
**ZINC03452729**	ago	-5.851836	-5.932923
**ZINC03463306**	ago	-6.954658	-3.779129
**ZINC03468792**	ago	-6.631565	-5.919455
**ZINC03468913**	ago	-7.456513	-6.416829
**ZINC03468921**	ago	-8.049289	-6.401083
**ZINC03471464**	ago	-6.503676	-6.040311
**ZINC03471543**	ago	-6.409449	-4.992666
**ZINC03473372**	ago	-6.739599	-5.780372
**ZINC03476460**	ago	-7.339909	-4.904691
**ZINC03479014**	ago	-7.201344	-6.463086
**ZINC03482000**	ago	-7.47217	-5.571359
**ZINC03487539**	ago	-7.314005	-6.707478
**ZINC03487542**	ago	-7.550217	-7.082305
**ZINC03487565**	ago	-6.875361	-7.20724
**ZINC03487567**	ago	-6.421719	-6.928095
**ZINC03504111**	ago	-6.684996	-6.218939
**ZINC03505438**	ago	-7.476188	-7.705584
**ZINC03505450**	ago	-7.655622	-7.124776
**ZINC03526474**	ago	-6.337971	-5.238128
**ZINC03532440**	ago	-6.2456	-6.310458

**ZINC03532447**	ago	-5.951204	-5.850737
**ZINC03546588**	ago	-6.100236	-5.908041
**ZINC03546810**	ago	-7.317723	-6.611272
**ZINC03546824**	ago	-6.553072	-6.525371
**ZINC03547621**	ago	-5.904657	-6.153829
**ZINC03547661**	ago	-6.935688	-6.561864
**ZINC03549771**	ago	-6.87042	-7.215267
**ZINC03558988**	ago	-6.487899	-5.540578
**ZINC03559027**	ago	-7.102012	-7.25626
**ZINC03566417**	ago	-6.776655	-5.50376
**ZINC03569979**	ago	-6.010393	-7.09457
**ZINC03572257**	ago	-7.377085	-5.966443
**ZINC03572281**	ago	-8.041303	-5.317678
**ZINC03572943**	ago	-8.017298	-7.232505
**ZINC03572944**	ago	-7.633046	-7.542991
**ZINC03573684**	ago	-6.893672	-5.120945
**ZINC03573733**	ago	-6.402786	-5.56199
**ZINC03574645**	ago	-6.467372	-5.226458
**ZINC03574662**	ago	-7.115851	-6.056678
**ZINC03574681**	ago	-6.405716	-5.416155
**ZINC03574730**	ago	-5.888702	-6.642334
**ZINC03580539**	ago	-7.361097	-6.450026
**ZINC03580562**	ago	-7.849544	-6.662832
**ZINC03580564**	ago	-8.368834	-6.513164
**ZINC03581786**	ago	-7.871787	-5.978833

**ZINC03584608**	ago	-6.687194	-6.263206
**ZINC03585126**	ago	-7.703001	-4.39738
**ZINC03588493**	ago	-7.091543	-7.005154
**ZINC03590134**	ago	-7.990763	-6.459463
**ZINC03590135**	ago	-7.926329	-6.965001
**ZINC03591896**	ago	-5.526817	-6.676407
**ZINC03597007**	ago	-7.713674	-7.562443
**ZINC03598522**	ago	-6.411501	-6.921073
**ZINC03600247**	ago	-7.679009	-6.574772
**ZINC03609376**	ago	-6.175719	-5.877829
**ZINC03609924**	ago	-6.435451	-6.015761
**ZINC03610252**	ago	-7.577338	-7.549654
**ZINC03612965**	ago	-6.780862	-5.751819
**ZINC03613804**	ago	-7.31067	-7.546287
**ZINC03616230**	ago	-7.895106	-5.622846
**ZINC03619635**	ago	-6.147983	-5.64556
**ZINC03619722**	ago	-7.051632	-5.735683
**ZINC03619825**	ago	-7.510111	-6.991823
**ZINC03631036**	ago	-6.927355	-6.591024
**ZINC03641076**	ago		
**ZINC03643624**	ago	-9.287937	-4.821655
**ZINC03646570**	ago	-7.820115	-6.671883
**ZINC03648115**	ago	-7.562862	-7.762807
**ZINC03648115**	ago	-7.271836	-7.521789
**ZINC03648115**	ago	-7.050982	-7.098797

**ZINC03648119**	ago	-7.898171	-7.574522
**ZINC03648119**	ago	-7.58796	-7.134317
**ZINC03648119**	ago	-7.070076	-6.683774
**ZINC03648127**	ago	-8.664012	-7.701004
**ZINC03648127**	ago	-7.637403	-6.800448
**ZINC03648127**	ago	-7.626821	-5.990652
**ZINC03648136**	ago	-8.373933	-7.768499
**ZINC03648136**	ago	-8.32078	-7.611178
**ZINC03648136**	ago	-8.135698	-6.763443
**ZINC03648139**	ago	-8.151316	-7.719157
**ZINC03648139**	ago	-8.109613	-7.564761
**ZINC03648139**	ago	-7.889012	-6.185357
**ZINC03648153**	ago	-7.970902	-7.276643
**ZINC03648153**	ago	-7.495819	-7.234173
**ZINC03648153**	ago	-7.084971	-6.512491
**ZINC03648155**	ago	-8.29614	-7.039862
**ZINC03648155**	ago	-7.380566	-6.480914
**ZINC03648155**	ago	-7.22879	-6.327933
**ZINC03652769**	ago	-9.302819	-9.298862
**ZINC03661716**	ago	-7.476585	-7.905586
**ZINC03668721**	ago	-8.529334	-4.346191
**ZINC03695689**	ago	-7.516522	-5.742382
**ZINC03698226**	ago	-7.430116	-5.674059
**ZINC03698250**	ago	-7.278011	-6.172988
**ZINC03698273**	ago	-7.511608	-6.662593

**ZINC03698280**	ago	-7.146524	-6.432527
**ZINC03698281**	ago	-6.944803	-6.622495
**ZINC03698282**	ago	-7.213788	-6.386286
**ZINC03701017**	ago	-7.102949	-5.313852
**ZINC03701019**	ago	-7.027982	-6.113388
**ZINC03701028**	ago	-7.250149	-5.043017
**ZINC03701034**	ago	-7.184828	-5.340687
**ZINC03701036**	ago	-7.163266	-6.375564
**ZINC03701869**	ago	-7.72246	-6.901701
**ZINC03704041**	ago	-6.649036	-6.885043
**ZINC03704548**	ago	-7.30601	-7.263074
**ZINC03704559**	ago	-7.595365	-7.201378
**ZINC03704568**	ago	-7.261494	-7.491294
**ZINC03704569**	ago	-7.50543	-7.276703
**ZINC03704574**	ago	-7.325358	-7.003148
**ZINC03704575**	ago	-7.447943	-6.339161
**ZINC03704590**	ago	-7.379735	-6.900183
**ZINC03704661**	ago	-7.902633	-7.698298
**ZINC03705708**	ago	-7.445745	-7.020709
**ZINC03706039**	ago	-7.579297	-6.405255
**ZINC03706060**	ago	-7.309648	-7.067018
**ZINC03706340**	ago	-7.893379	-7.753336
**ZINC03711133**	ago	-7.611381	-5.184366
**ZINC03711154**	ago	-6.926146	-6.59166
**ZINC03711229**	ago	-7.608495	-6.659878

**ZINC03711302**	ago	-7.876256	-6.484903
**ZINC03711330**	ago	-7.41033	-4.621766
**ZINC03711332**	ago	-7.074821	-6.173783
**ZINC03715970**	ago	-6.996395	-7.065104
**ZINC03778001**	ago	-8.200134	-5.364228
**ZINC03778005**	ago	-8.487028	-7.978464
**ZINC03778009**	ago	-8.250651	-4.798345
**ZINC03778012**	ago	-7.760184	-4.229594
**ZINC03779780**	ago	-7.395458	-7.386526
**ZINC03787862**	ago	-7.861113	-7.752185
**ZINC03796900**	ago	-7.617654	-7.078582
**ZINC03802111**	ago	-6.911399	-7.029086
**ZINC03802112**	ago	-8.059608	-7.432919
**ZINC03802119**	ago	-6.823454	-7.161892
**ZINC03805109**	ago	-7.063443	-7.186495
**ZINC03806773**	ago	-8.184067	-5.124268
**ZINC03809953**	ago	-7.816651	-7.471183
**ZINC03813975**	ago	-7.990818	-5.003343
**ZINC03817440**	ago	-7.742103	-6.882341
**ZINC03817444**	ago	-8.121009	-7.380381
**ZINC03817448**	ago	-8.304045	-7.327302
**ZINC03819167**	ago	-8.877731	-8.460046
**ZINC03820698**	ago	-6.559101	-7.194963
**ZINC03820698**	ago	-6.228256	-6.370622
**ZINC03820700**	ago	-6.904395	-6.860947

**ZINC03820700**	ago	-5.913938	-6.731659
**ZINC03833048**	ago	-6.890251	-6.536529
**ZINC03833048**	ago	-6.890251	-6.279173
**ZINC03833048**	ago	-5.44325	-6.279173
**ZINC03836286**	ago	-8.180382	-7.586452
**ZINC03845596**	ago	-7.13279	-5.941396
**ZINC03846131**	ago	-7.151968	-6.299233
**ZINC03846353**	ago	-7.640341	-5.659802
**ZINC03848076**	ago	-6.947913	-6.667702
**ZINC03848552**	ago	-6.354933	-5.919412
**ZINC03848643**	ago	-7.735538	-6.478984
**ZINC03848978**	ago	-8.021178	-5.935093
**ZINC03848978**	ago	-7.865797	-5.76298
**ZINC03849549**	ago	-8.234636	-7.613678
**ZINC03849556**	ago	-8.382626	-4.668744
**ZINC03849897**	ago	-7.795027	-5.269784
**ZINC03849900**	ago	-7.526558	-5.339499
**ZINC03850583**	ago	-8.16646	-5.464946
**ZINC03850676**	ago	-8.118003	-7.382563
**ZINC03850705**	ago	-8.063099	-7.434879
**ZINC03850712**	ago	-7.698388	-7.34588
**ZINC03850792**	ago	-8.123856	-6.653084
**ZINC03851020**	ago		-5.04259
**ZINC03851063**	ago	-7.886317	-7.563913
**ZINC03851063**	ago	-7.492952	-7.016534

**ZINC03851155**	ago	-7.798477	-5.586058
**ZINC03851187**	ago	-8.111179	-7.036603
**ZINC03851218**	ago	-7.65647	-7.457122
**ZINC03851251**	ago	-7.738155	-5.505337
**ZINC03851252**	ago	-7.43807	-6.928696
**ZINC03851837**	ago	-8.226296	-6.190568
**ZINC03852165**	ago	-8.066049	-5.247152
**ZINC03852213**	ago	-7.852521	-6.67859
**ZINC03852765**	ago	-6.792403	-6.356061
**ZINC03852771**	ago	-6.824741	-6.637858
**ZINC03852815**	ago	-7.281732	-7.537227
**ZINC03852826**	ago	-7.185304	-6.161262
**ZINC03852836**	ago	-7.143246	-6.302113
**ZINC03852854**	ago	-6.710125	-5.993026
**ZINC03852898**	ago	-6.120973	-5.64034
**ZINC03852923**	ago	-7.577202	-6.664943
**ZINC03853328**	ago	-6.463834	-5.214279
**ZINC03853398**	ago		-6.227954
**ZINC03853405**	ago	-6.31558	-5.884256
**ZINC03853430**	ago	-7.193865	-5.547047
**ZINC03853446**	ago		-5.494225
**ZINC03853447**	ago	-7.082957	-4.145464
**ZINC03853524**	ago	-7.573293	-6.450369
**ZINC03853525**	ago	-8.210159	-7.669731
**ZINC03853575**	ago	-7.779305	-7.079689

**ZINC03853619**	ago	-6.29913	-5.641436
**ZINC03853624**	ago	-7.425796	-5.81959
**ZINC03853736**	ago	-6.824611	-6.426603
**ZINC03853737**	ago	-7.400668	-7.063434
**ZINC03853942**	ago	-7.793025	-5.283775
**ZINC03853943**	ago	-7.67445	-5.163475
**ZINC03853944**	ago	-8.058928	-7.121095
**ZINC03853945**	ago	-8.145623	-4.591951
**ZINC03853947**	ago	-7.833415	-5.148124
**ZINC03853948**	ago	-7.718549	-6.358479
**ZINC03853949**	ago	-6.31796	-6.312608
**ZINC03853950**	ago	-8.128831	-4.609243
**ZINC03854328**	ago		-6.746601
**ZINC03854329**	ago		-4.355474
**ZINC03854572**	ago	-8.136002	-7.324451
**ZINC03854573**	ago	-7.236731	-6.621208
**ZINC03854914**	ago	-7.653291	-6.204902
**ZINC03854982**	ago	-5.972377	-6.873356
**ZINC03855047**	ago	-6.883575	-5.452923
**ZINC03855064**	ago	-7.680803	-6.817688
**ZINC03855970**	ago	-7.665194	-4.832943
**ZINC03855990**	ago	-7.537809	-7.008037
**ZINC03855991**	ago	-7.640934	-7.000681
**ZINC03856290**	ago	-8.056477	-7.859264
**ZINC03857060**	ago	-7.4234	-5.897949

**ZINC03857437**	ago	-7.462619	
**ZINC03857826**	ago		-5.707522
**ZINC03857827**	ago		-5.396691
**ZINC03857921**	ago	-8.203137	-3.821606
**ZINC03858024**	ago	-7.965833	-5.478267
**ZINC03858024**	ago	-7.632367	-4.981354
**ZINC03858227**	ago		-5.58674
**ZINC03858228**	ago	-7.268727	-6.084415
**ZINC03858229**	ago	-5.658579	-6.259516
**ZINC03858231**	ago		
**ZINC03858386**	ago	-8.670981	-8.065127
**ZINC03858425**	ago	-6.548346	-6.448243
**ZINC03858620**	ago	-7.266772	-6.077389
**ZINC03858879**	ago	-9.06387	-7.941253
**ZINC03859198**	ago	-7.751275	-7.387913
**ZINC03859217**	ago	-7.03744	-6.836174
**ZINC03859218**	ago	-7.708972	-4.721471
**ZINC03859315**	ago	-7.416252	-7.240415
**ZINC03859494**	ago		
**ZINC03859539**	ago		
**ZINC03859894**	ago	-7.380265	-7.132355
**ZINC03859894**	ago	-6.997831	-6.754354
**ZINC03860040**	ago	-7.543618	-5.409648
**ZINC03860056**	ago	-7.539531	-5.430489
**ZINC03861812**	ago	-7.502043	-6.463919

**ZINC03861813**	ago	-7.894622	-5.934978
**ZINC03861814**	ago	-7.803905	-6.378443
**ZINC03861816**	ago	-7.029335	-5.052
**ZINC03861829**	ago	-7.624901	-4.986473
**ZINC03861830**	ago	-7.984376	-5.660374
**ZINC03861869**	ago	-6.202602	-6.100714
**ZINC03861870**	ago	-6.356614	-4.170488
**ZINC03861873**	ago	-8.094772	-5.566268
**ZINC03861874**	ago	-7.890968	-7.237615
**ZINC03861875**	ago	-7.789163	-6.858379
**ZINC03861876**	ago	-7.709937	-7.234724
**ZINC03861881**	ago	-7.711003	-6.739372
**ZINC03861882**	ago	-8.12346	
**ZINC03861883**	ago	-7.637518	-6.894977
**ZINC03861885**	ago	-7.000575	-4.775692
**ZINC03861910**	ago	-7.99869	-6.706904
**ZINC03861911**	ago	-7.63382	-6.858622
**ZINC03861912**	ago	-7.781427	-7.395471
**ZINC03861933**	ago	-7.284091	-5.628454
**ZINC03861936**	ago	-7.209522	-5.613854
**ZINC03861937**	ago	-7.346984	-7.247368
**ZINC03862019**	ago	-8.199993	-5.700569
**ZINC03862064**	ago	-7.338226	-5.073509
**ZINC03862065**	ago	-7.819396	-7.214199
**ZINC03862142**	ago	-8.275973	-7.41515

**ZINC03862151**	ago	-8.192152	-5.189492
**ZINC03862171**	ago	-8.011436	-5.561045
**ZINC03862230**	ago	-7.881173	-5.109028
**ZINC03862237**	ago	-7.073703	-8.927106
**ZINC03862253**	ago	-7.811583	-4.819073
**ZINC03862256**	ago	-7.46695	-7.4019
**ZINC03862293**	ago	-8.006641	-6.99788
**ZINC03862360**	ago	-7.252852	-7.14709
**ZINC03862391**	ago	-7.237747	-5.820144
**ZINC03862416**	ago	-7.791804	-4.60651
**ZINC03862417**	ago	-7.103442	-5.595721
**ZINC03862418**	ago	-6.901222	-5.381686
**ZINC03862419**	ago	-7.394175	-5.199703
**ZINC03862420**	ago	-6.994237	-4.058701
**ZINC03862432**	ago		
**ZINC03862452**	ago	-9.787376	-7.913775
**ZINC03862520**	ago	-8.129167	-5.003807
**ZINC03862521**	ago	-6.534032	-6.697699
**ZINC03862640**	ago	-7.270595	-6.40497
**ZINC03862703**	ago	-8.073545	-5.602203
**ZINC03862796**	ago	-8.449769	-4.500328
**ZINC03862797**	ago	-7.729071	-4.912469
**ZINC03862803**	ago	-7.601447	-7.142782
**ZINC03862842**	ago	-7.211812	-6.40874
**ZINC03862848**	ago	-7.951501	-4.439921

**ZINC03862849**	ago	-7.638079	-4.661128
**ZINC03862990**	ago	-6.26787	-5.380614
**ZINC03863011**	ago	-7.628804	-8.105853
**ZINC03863071**	ago	-8.106329	-4.558367
**ZINC03863221**	ago	-7.094461	-7.707531
**ZINC03863289**	ago	-7.14455	-4.571597
**ZINC03863290**	ago	-7.005359	-5.179432
**ZINC03863323**	ago	-7.656479	-7.268459
**ZINC03863327**	ago	-7.454683	
**ZINC03863352**	ago	-7.887804	-8.055418
**ZINC03863357**	ago	-7.068402	-5.200023
**ZINC03863381**	ago	-7.105133	-7.910633
**ZINC03863395**	ago	-7.574926	-5.254354
**ZINC03863536**	ago	-8.033714	-6.814249
**ZINC03863665**	ago	-5.226571	
**ZINC03863700**	ago	-7.995167	-7.357493
**ZINC03863701**	ago	-7.857129	-7.657716
**ZINC03863745**	ago	-7.883886	-4.72097
**ZINC03863763**	ago	-7.908243	-4.68625
**ZINC03863843**	ago	-7.119911	-5.766037
**ZINC03863893**	ago	-6.806966	-3.975174
**ZINC03863904**	ago	-7.093067	-4.544768
**ZINC03863905**	ago	-5.960319	-5.024269
**ZINC03863906**	ago	-7.298993	-5.121697
**ZINC03863907**	ago	-7.213298	-5.410392

**ZINC03863941**	ago	-7.300732	-5.481361
**ZINC03863965**	ago	-7.41917	-5.411742
**ZINC03863986**	ago	-6.826341	-5.540794
**ZINC03866672**	ago	-6.510426	-6.323414
**ZINC03866673**	ago	-7.395397	-4.154768
**ZINC03866716**	ago	-7.341334	-6.199684
**ZINC03866910**	ago	-7.999298	-6.134882
**ZINC03866924**	ago	-6.105428	-5.718511
**ZINC03866931**	ago	-9.144056	-7.449108
**ZINC03866974**	ago	-7.75269	-6.841273
**ZINC03867001**	ago	-8.303286	-4.525408
**ZINC03867154**	ago	-7.517745	-8.320871
**ZINC03867158**	ago	-7.718497	-7.443847
**ZINC03867187**	ago	-7.071435	-5.034396
**ZINC03867188**	ago	-6.660101	-6.54574
**ZINC03867208**	ago	-6.474308	-6.278125
**ZINC03867211**	ago	-8.98049	-5.967721
**ZINC03867215**	ago	-6.877068	-5.412829
**ZINC03867252**	ago	-2.215813	-3.151694
**ZINC03867316**	ago	-6.229101	-6.752184
**ZINC03867316**	ago	-5.50961	-5.550808
**ZINC03867424**	ago	-8.397583	-6.884333
**ZINC03867425**	ago	-8.166799	-6.232223
**ZINC03867426**	ago	-6.672592	-6.380778
**ZINC03867453**	ago	-6.939312	-5.863276

**ZINC03867544**	ago	-7.79891	-7.582488
**ZINC03867544**	ago	-7.773574	-5.906393
**ZINC03867654**	ago	-7.077853	-6.770118
**ZINC03867760**	ago	-8.218216	-4.722827
**ZINC03867761**	ago	-8.481144	-4.909836
**ZINC03867897**	ago	-5.902707	-5.744546
**ZINC03868035**	ago	-6.977646	-6.090509
**ZINC03868036**	ago	-6.312021	-7.159524
**ZINC03868037**	ago	-8.1137	-6.828366
**ZINC03868038**	ago	-5.607304	-6.191334
**ZINC03868039**	ago	-7.792367	-6.64675
**ZINC03868067**	ago	-7.805741	-6.927064
**ZINC03868068**	ago	-7.582658	-6.771856
**ZINC03868185**	ago	-6.430542	-6.362264
**ZINC03868186**	ago	-6.820447	-5.972873
**ZINC03868187**	ago	-5.739747	-5.499387
**ZINC03868222**	ago	-7.903932	-4.788408
**ZINC03868321**	ago	-7.45921	-5.649833
**ZINC03868322**	ago	-8.360762	-7.276241
**ZINC03868323**	ago	-8.398588	-7.590295
**ZINC03868328**	ago	-7.56409	-5.896372
**ZINC03868329**	ago	-7.077166	-6.721496
**ZINC03868350**	ago	-8.222525	-6.849187
**ZINC03868351**	ago	-7.692385	-6.388715
**ZINC03868413**	ago	-7.441987	-6.944877

**ZINC03868452**	ago	-6.662719	-6.319227
**ZINC03868474**	ago	-8.326475	-7.073487
**ZINC03868495**	ago	-6.391615	-4.49434
**ZINC03868497**	ago	-7.156011	-5.702599
**ZINC03868498**	ago	-6.390215	-4.839246
**ZINC03868512**	ago	-7.690971	-6.928609
**ZINC03868596**	ago		-5.763132
**ZINC03868607**	ago	-7.748035	-4.989833
**ZINC03868608**	ago	-7.572944	-7.063401
**ZINC03868874**	ago	-7.222536	-7.091906
**ZINC03868875**	ago	-7.136618	-6.696011
**ZINC03868876**	ago	-7.433329	-7.057636
**ZINC03868877**	ago	-7.879761	-6.718938
**ZINC03868878**	ago	-7.748901	-6.702699
**ZINC03868879**	ago	-7.848923	-7.258885
**ZINC03868880**	ago	-7.400751	-7.245015
**ZINC03868881**	ago	-7.419555	-6.584555
**ZINC03868882**	ago	-7.740425	-6.938467
**ZINC03868883**	ago	-7.818495	-6.599705
**ZINC03868884**	ago	-8.015405	-6.491136
**ZINC03868885**	ago	-7.942558	-6.700519
**ZINC03868886**	ago	-7.863128	-6.584935
**ZINC03868887**	ago	-7.481152	-6.925306
**ZINC03868889**	ago	-7.455419	-7.080219
**ZINC03868890**	ago	-7.625565	-7.113289

**ZINC03868891**	ago	-7.972596	-7.269236
**ZINC03868892**	ago	-7.727711	-7.65872
**ZINC03868893**	ago	-7.754014	-7.834257
**ZINC03868894**	ago	-7.912706	-7.390953
**ZINC03874005**	ago	-7.85371	-6.771388
**ZINC03874293**	ago	-7.925849	-7.254899
**ZINC03877847**	ago	-7.689719	-4.551014
**ZINC03878190**	ago	-6.848557	-6.549766
**ZINC03878696**	ago	-7.384753	-6.760528
**ZINC03880449**	ago	-7.856022	-6.589673
**ZINC03882740**	ago	-8.104032	-7.688637
**ZINC03883435**	ago	-7.399463	-6.515319
**ZINC03883463**	ago	-7.69765	-5.06319
**ZINC03885633**	ago	-7.496307	-8.298627
**ZINC03886022**	ago	-7.619887	-6.755759
**ZINC03886022**	ago	-7.510266	-6.710891
**ZINC03887063**	ago	-7.335639	-6.744812
**ZINC03887961**	ago	-6.969858	-6.620669
**ZINC03888352**	ago	-7.271313	-7.087171
**ZINC03888352**	ago	-7.271313	-7.087171
**ZINC03889080**	ago	-7.732182	-5.849591
**ZINC03889103**	ago	-7.482509	-4.653749
**ZINC03889189**	ago	-6.815195	-5.77177
**ZINC03889197**	ago	-8.779748	-5.791115
**ZINC03889373**	ago	-7.117384	-6.377842

**ZINC03889479**	ago	-7.438561	-4.365217
**ZINC03889530**	ago	-6.689276	-4.842077
**ZINC03889532**	ago	-7.538111	-3.897594
**ZINC03889557**	ago	-7.290313	-6.138853
**ZINC03889602**	ago	-8.387368	-6.333408
**ZINC03889697**	ago	-7.05504	-7.065665
**ZINC03889731**	ago	-7.567316	-5.386247
**ZINC03889768**	ago	-8.841782	-7.890425
**ZINC03890018**	ago	-8.131724	-4.716773
**ZINC03890022**	ago	-6.8386	-8.251186
**ZINC03890024**	ago	-7.662401	-7.967064
**ZINC03890042**	ago	-8.520102	-6.907945
**ZINC03890043**	ago	-8.875626	-8.388872
**ZINC03890045**	ago	-7.693124	-5.342962
**ZINC03890049**	ago	-8.695285	-7.193472
**ZINC03890095**	ago	-8.296517	-7.02834
**ZINC03890097**	ago	-8.597628	-5.887044
**ZINC03890259**	ago	-7.390102	-6.860993
**ZINC03890261**	ago	-8.221468	-7.531158
**ZINC03890263**	ago	-7.299977	-6.36559
**ZINC03890335**	ago	-6.952373	-6.09004
**ZINC03890444**	ago	-7.38487	-5.970511
**ZINC03890549**	ago	-7.26808	-5.448871
**ZINC03890563**	ago	-6.743827	-6.922473
**ZINC03890604**	ago	-7.415938	-7.244965

**ZINC03890623**	ago	-8.281486	-6.576346
**ZINC03890725**	ago	-7.467664	-7.039444
**ZINC03890726**	ago	-7.672073	-7.064915
**ZINC03890733**	ago	-7.805935	-4.475688
**ZINC03890734**	ago	-7.032188	-5.232862
**ZINC03890798**	ago	-6.050379	
**ZINC03890985**	ago	-7.046347	-6.584038
**ZINC03890986**	ago	-7.024918	-6.184483
**ZINC03891002**	ago	-6.601763	-4.510461
**ZINC03891014**	ago	-7.432719	-7.237569
**ZINC03891550**	ago	-6.794958	-6.454414
**ZINC03891594**	ago	-7.757697	-6.675367
**ZINC03891595**	ago	-7.680504	-6.619955
**ZINC03891603**	ago	-7.530361	-6.441857
**ZINC03891604**	ago	-7.733854	-4.784293
**ZINC03891611**	ago	-8.101741	-4.87316
**ZINC03891627**	ago	-6.703095	-6.082111
**ZINC03891773**	ago	-7.866783	-7.930281
**ZINC03891774**	ago	-7.270888	-7.701494
**ZINC03891776**	ago	-7.81657	-4.907264
**ZINC03891789**	ago	-7.924791	-7.50077
**ZINC03891790**	ago	-6.829207	-5.548819
**ZINC03891791**	ago	-7.567874	-5.101592
**ZINC03891889**	ago	-7.588045	-7.739789
**ZINC03891997**	ago	-5.915114	-5.928422

**ZINC03891998**	ago	-6.542851	-5.552899
**ZINC03891998**	ago	-6.532006	-5.399794
**ZINC03892041**	ago	-7.363659	-6.695941
**ZINC03892042**	ago	-7.623597	-7.262824
**ZINC03892093**	ago	-7.927903	-5.378921
**ZINC03892156**	ago	-7.75928	-6.962503
**ZINC03892157**	ago	-7.674713	-7.719391
**ZINC03892170**	ago	-7.214116	-3.985923
**ZINC03892890**	ago	-6.518037	-4.578125
**ZINC03895516**	ago	-5.541365	-6.27271
**ZINC03896497**	ago	-8.037479	-7.819741
**ZINC03896497**	ago	-8.017106	-5.572291
**ZINC03896507**	ago	-8.017728	-7.85337
**ZINC03896507**	ago	-7.957108	-7.421915
**ZINC03898183**	ago	-7.695215	-4.987631
**ZINC03899187**	ago	-7.904982	-5.647673
**ZINC03902335**	ago	-7.567793	-4.179956
**ZINC03902340**	ago	-7.282054	-5.442416
**ZINC03902342**	ago	-7.001966	-5.806985
**ZINC03902359**	ago	-7.287048	-5.919325
**ZINC03903640**	ago	-7.254981	-6.38779
**ZINC03903640**	ago	-7.088434	-5.255879
**ZINC03905729**	ago	-6.645163	-6.763961
**ZINC03905742**	ago	-7.17852	-6.73856
**ZINC03912081**	ago	-8.060924	-7.170043

**ZINC03912082**	ago	-8.259527	-7.098224
**ZINC03912090**	ago	-7.989045	-7.449677
**ZINC03912094**	ago	-8.213951	-4.405179
**ZINC03912100**	ago	-7.477182	-5.634013
**ZINC03912122**	ago	-7.832361	-7.740147
**ZINC03912123**	ago	-8.283965	-7.125397
**ZINC03912352**	ago	-7.624234	-5.53752
**ZINC03912414**	ago	-7.236563	-4.790748
**ZINC03912567**	ago	-7.816849	-4.574198
**ZINC03912587**	ago	-7.635125	-7.294239
**ZINC03912645**	ago	-7.939067	-7.58684
**ZINC03912819**	ago	-7.857843	-7.610255
**ZINC03915640**	ago	-7.585426	-6.910639
**ZINC03917600**	ago	-6.849084	-6.889021
**ZINC03917924**	ago	-6.82587	-5.039128
**ZINC03922122**	ago	-6.949731	-6.397714
**ZINC03927944**	ago	-9.938572	-6.202825
**ZINC03938273**	ago	-6.948443	-6.107678
**ZINC03943315**	ago	-6.613734	-5.761061
**ZINC03943315**	ago	-6.104388	-5.545684
**ZINC03944815**	ago	-7.052192	-6.855304
**ZINC03944815**	ago	-6.54794	-6.789694
**ZINC03944975**	ago	-8.180135	-8.230775
**ZINC03944975**	ago	-8.154865	-7.802121
**ZINC03949689**	ago	-8.063386	-7.446437

**ZINC03949691**	ago	-7.179114	-7.472979
**ZINC03950321**	ago	-9.861828	-9.766078
**ZINC03952329**	ago	-7.8981	-7.704099
**ZINC03952974**	ago	-9.018281	-9.236966
**ZINC03955270**	ago	-7.148518	-6.650268
**ZINC03955272**	ago	-6.740599	-6.845171
**ZINC03956414**	ago	-6.273521	-5.979172
**ZINC03956441**	ago	-7.609422	-7.129721
**ZINC03956453**	ago	-7.533973	-5.793848
**ZINC03956474**	ago	-8.005915	-7.06135
**ZINC03956502**	ago	-8.357846	-6.686188
**ZINC03957527**	ago	-6.940102	-6.239196
**ZINC03957585**	ago	-8.440834	-5.523581
**ZINC03958082**	ago	-8.152088	-7.205587
**ZINC03958184**	ago	-8.119353	-7.689827
**ZINC03958189**	ago	-7.527592	-5.55075
**ZINC03958220**	ago	-7.15792	-6.797045
**ZINC03958436**	ago	-8.526617	-4.901638
**ZINC03959122**	ago	-7.46061	-7.063634
**ZINC03959140**	ago	-7.576101	-4.22529
**ZINC03960801**	ago	-6.901183	-7.332639
**ZINC03961119**	ago	-8.476185	-6.672833
**ZINC03961129**	ago	-6.975381	-7.394442
**ZINC03962055**	ago	-7.887875	-4.864509
**ZINC03962070**	ago	-8.127586	-7.498879

**ZINC03962117**	ago	-7.563935	-5.391232
**ZINC03962400**	ago	-8.249799	-7.215948
**ZINC03962425**	ago	-7.221716	-5.321097
**ZINC03962434**	ago	-7.775439	-7.084088
**ZINC03962648**	ago	-7.775758	-6.421784
**ZINC03962706**	ago	-7.95473	-4.746438
**ZINC03963037**	ago	-6.542989	-5.381764
**ZINC03963228**	ago	-7.801628	-5.817172
**ZINC03963552**	ago	-7.85682	-5.677136
**ZINC03963666**	ago	-7.154876	-4.946585
**ZINC03963684**	ago	-7.121966	-6.735214
**ZINC03963807**	ago	-7.22293	-7.376469
**ZINC03963873**	ago	-7.611446	-7.246809
**ZINC03963876**	ago	-7.998246	-7.368324
**ZINC03963895**	ago	-7.986247	-7.128131
**ZINC03963904**	ago	-8.030448	-6.758613
**ZINC03963905**	ago	-7.921089	-7.564841
**ZINC03963908**	ago	-7.628343	-8.144415
**ZINC03963912**	ago	-8.220254	-7.566159
**ZINC03963923**	ago	-8.084705	-8.105519
**ZINC03963929**	ago	-8.300972	-6.85985
**ZINC03963934**	ago	-7.866455	-7.578069
**ZINC03963936**	ago	-8.104434	-7.879272
**ZINC03963938**	ago	-8.204475	-7.713184
**ZINC03963939**	ago	-7.943935	-7.390094

**ZINC03963941**	ago	-8.034915	-7.140412
**ZINC03963950**	ago	-7.995157	-7.669619
**ZINC03963954**	ago		-6.275945
**ZINC03963957**	ago	-7.836969	-6.077444
**ZINC03964214**	ago	-9.04736	-6.679847
**ZINC03967002**	ago	-7.12257	-7.384292
**ZINC03967617**	ago	-4.87955	-5.959664
**ZINC03967620**	ago	-7.193447	-5.850189
**ZINC03967658**	ago	-6.759217	-5.543519
**ZINC03967912**	ago	-5.659142	-5.708325
**ZINC03968433**	ago	-6.555942	-5.62335
**ZINC03968434**	ago	-7.896137	-6.332313
**ZINC03968512**	ago	-7.080303	-5.892844
**ZINC03968545**	ago	-7.475615	-6.115748
**ZINC03968549**	ago	-6.948953	-5.631819
**ZINC03968602**	ago	-7.422839	-5.526261
**ZINC03968609**	ago	-7.169691	-5.424365
**ZINC03968652**	ago	-8.330019	-6.500944
**ZINC03968673**	ago	-7.497192	-6.295805
**ZINC03968674**	ago	-8.220534	-5.896694
**ZINC03968753**	ago	-6.570456	-5.99526
**ZINC03968753**	ago	-6.273546	-5.66161
**ZINC03968761**	ago	-7.260101	-6.035073
**ZINC03968762**	ago	-6.612257	-6.184477
**ZINC03969325**	ago	-7.608176	-7.963459

**ZINC03969763**	ago	-7.673796	-4.826382
**ZINC03970837**	ago	-7.419466	-7.610637
**ZINC03970838**	ago	-7.066067	-7.392998
**ZINC03970911**	ago	-7.937314	-6.256061
**ZINC03970916**	ago	-7.564535	-3.98502
**ZINC03971344**	ago	-5.923371	-5.122023
**ZINC03971345**	ago	-7.172236	-5.690325
**ZINC03971488**	ago	-6.303362	-6.072761
**ZINC03971624**	ago	-7.576033	-5.967099
**ZINC03972165**	ago	-8.180057	-5.285691
**ZINC03972166**	ago	-7.501085	-7.000503
**ZINC03972174**	ago	-8.057531	-6.448535
**ZINC03972183**	ago	-8.833809	-7.350002
**ZINC03973577**	ago	-6.106278	-5.040349
**ZINC03973862**	ago	-7.691854	-4.806041
**ZINC03974487**	ago	-8.750547	-8.354338
**ZINC03974922**	ago	-7.699042	-5.689693
**ZINC03974923**	ago	-7.095861	-6.408715
**ZINC03974925**	ago	-7.348993	-5.996981
**ZINC03974999**	ago	-7.975312	-5.975538
**ZINC03975000**	ago	-7.093681	-5.35265
**ZINC03975208**	ago	-7.484813	-5.305514
**ZINC03975965**	ago	-8.05347	
**ZINC03975967**	ago	-6.053481	
**ZINC03977594**	ago	-7.265272	-4.69958

**ZINC03977633**	ago	-6.470711	-5.205848
**ZINC03977634**	ago	-7.363086	-6.932502
**ZINC03977636**	ago	-6.90949	-6.227668
**ZINC03977652**	ago	-8.489336	-7.210591
**ZINC03977653**	ago	-7.166677	-6.647447
**ZINC03977660**	ago	-8.191343	-6.384211
**ZINC03977692**	ago	-6.937744	-6.493035
**ZINC03977693**	ago	-6.815175	-5.284343
**ZINC03977698**	ago	-7.128211	-4.796294
**ZINC03980039**	ago	-7.018334	-6.603021
**ZINC03980039**	ago	-6.163388	-6.302998
**ZINC03980300**	ago	-7.562617	-7.663185
**ZINC03980300**	ago	-7.345173	-7.200932
**ZINC03980304**	ago	-7.997627	-7.544003
**ZINC03980304**	ago	-7.933288	-4.353537
**ZINC03987446**	ago	-7.805507	-5.948831
**ZINC03987653**	ago	-6.480471	-5.445325
**ZINC03990121**	ago	-8.416428	-8.149782
**ZINC03990121**	ago	-8.373813	-8.025441
**ZINC03990124**	ago	-8.043836	-6.441419
**ZINC03990124**	ago	-6.795229	-6.232224
**ZINC03993180**	ago	-6.714137	-6.262687
**ZINC03993180**	ago	-6.521446	-5.815021
**ZINC03993213**	ago	-8.23005	-4.142635
**ZINC03995276**	ago	-6.645003	-4.355734

**ZINC03995667**	ago	-9.29009	-8.658222
**ZINC03995667**	ago		-8.569339
**ZINC03995667**	ago		
**ZINC03997271**	ago	-8.703375	-7.316787
**ZINC03997278**	ago	-7.534396	-5.444643
**ZINC03997291**	ago	-7.276784	-7.814094
**ZINC03997329**	ago	-7.592614	-6.976803
**ZINC03997420**	ago	-7.085065	-5.232903
**ZINC03998675**	ago	-7.630338	-4.409498
**ZINC03998897**	ago	-7.243261	-7.015723
**ZINC04000726**	ago	-7.171753	-6.878962
**ZINC04001281**	ago		-4.733456
**ZINC04001309**	ago	-8.091371	
**ZINC04001310**	ago		
**ZINC04003655**	ago	-6.618472	-7.014213
**ZINC04003811**	ago	-7.841277	-7.107248
**ZINC04007242**	ago	-8.005647	-5.031138
**ZINC04010331**	ago	-8.219332	-6.438892
**ZINC04010331**	ago	-7.889028	-5.934284
**ZINC04011518**	ago	-7.770875	-4.517585
**ZINC04011561**	ago	-8.292425	-6.902699
**ZINC04012477**	ago	-7.720431	-7.064078
**ZINC04015227**	ago	-6.859846	-4.625085
**ZINC04021979**	ago	-7.900568	-5.428612
**ZINC04023848**	ago	-7.43554	-7.386498

**ZINC04023848**	ago	-6.871102	-7.361046
**ZINC04024702**	ago	-7.853821	-5.67651
**ZINC04024702**	ago	-7.124678	-5.447966
**ZINC04024702**	ago	-6.858772	-5.327535
**ZINC04025542**	ago	-7.823077	-7.054879
**ZINC04025542**	ago	-7.729924	-6.718056
**ZINC04030205**	ago	-7.185895	-5.922087
**ZINC04030688**	ago	-7.397971	-6.863934
**ZINC04031442**	ago	-7.43876	-4.602544
**ZINC04036395**	ago	-6.857053	-6.220646
**ZINC04039942**	ago	-7.682959	-7.250472
**ZINC04045166**	ago	-7.790776	-7.999732
**ZINC04045945**	ago	-7.263647	-4.658564
**ZINC04045947**	ago	-6.814894	-5.640378
**ZINC04046285**	ago	-7.871503	-8.080166
**ZINC04052857**	ago	-7.99279	-7.172722
**ZINC04053914**	ago	-7.231819	-6.073186
**ZINC04054541**	ago	-6.523055	-6.240751
**ZINC04059952**	ago	-5.798246	-7.627952
**ZINC04059952**	ago	-5.575866	-7.164731
**ZINC04066451**	ago	-8.000396	-5.817534
**ZINC04073094**	ago	-7.75255	-7.908962
**ZINC04084789**	ago	-7.855776	-8.004329
**ZINC04084803**	ago	-7.650844	-8.22794
**ZINC04091645**	ago	-6.01569	-5.713201

**ZINC04093701**	ago	-6.422291	-5.897129
**ZINC04093831**	ago	-7.318824	-7.515389
**ZINC04094697**	ago	-8.972873	-7.588932
**ZINC04094697**	ago	-7.511902	-7.534157
**ZINC04094698**	ago	-7.328905	-6.321202
**ZINC04094698**	ago	-7.086079	-6.027259
**ZINC04095457**	ago	-7.385926	-6.843229
**ZINC04104318**	ago	-7.268253	-7.338877
**ZINC04104321**	ago	-7.599284	-7.226595
**ZINC04104394**	ago	-7.937471	-4.690598
**ZINC04107371**	ago	-6.19591	-6.261727
**ZINC04111124**	ago	-7.297761	-5.282797
**ZINC04111523**	ago		
**ZINC04114008**	ago	-7.2838	-5.314744
**ZINC04114861**	ago	-8.028099	-7.897299
**ZINC04114861**	ago	-7.818587	-7.393975
**ZINC04116089**	ago	-7.485347	-6.809959
**ZINC04116089**	ago	-7.450407	-6.744161
**ZINC04117622**	ago	-7.47774	-5.602487
**ZINC04118124**	ago	-7.258521	-6.270226
**ZINC04121849**	ago	-7.625924	-6.986331
**ZINC04125148**	ago	-8.104646	-6.866268
**ZINC04125498**	ago	-7.374646	-7.126187
**ZINC04129069**	ago	-8.031457	-4.317836
**ZINC04131904**	ago	-7.083479	-3.311711

**ZINC04132179**	ago	-8.614612	-5.479014
**ZINC04135833**	ago	-7.499987	-6.634799
**ZINC04135874**	ago	-6.904258	-6.242056
**ZINC04135874**	ago	-6.760572	-5.721287
**ZINC04136953**	ago	-7.540872	-6.2244
**ZINC04136967**	ago	-7.425546	-5.090947
**ZINC04138128**	ago	-6.408909	-5.605351
**ZINC04138328**	ago	-7.526138	-5.697014
**ZINC04139348**	ago	-7.692757	-5.419089
**ZINC04139377**	ago		
**ZINC04139377**	ago		
**ZINC04139377**	ago		
**ZINC04147175**	ago	-7.868347	-5.008334
**ZINC04166104**	ago	-7.35969	-5.665153
**ZINC04177102**	ago	-6.306897	-5.841355
**ZINC04177114**	ago	-7.342813	-6.070477
**ZINC04183653**	ago	-8.130478	-4.724757
**ZINC04193893**	ago	-7.727388	-7.686672
**ZINC04193893**	ago	-7.180582	-5.184591
**ZINC04217587**	ago	-7.597157	-6.974447
**ZINC04227359**	ago	-7.239576	-7.275436
**ZINC04234725**	ago	-7.823847	-7.515728
**ZINC04235099**	ago	-6.912903	-6.792686
**ZINC04235328**	ago	-7.581262	-6.783521
**ZINC04235376**	ago	-6.990236	-6.040438

**ZINC04245274**	ago	-7.668506	-6.851931
**ZINC04245307**	ago	-6.830717	-6.204614
**ZINC04251127**	ago	-7.486213	-6.713689
**ZINC04251127**	ago	-7.31163	-6.299839
**ZINC04251127**	ago	-7.279028	-5.961945
**ZINC04266959**	ago	-7.625073	-7.357488
**ZINC04268527**	ago	-6.782008	-6.530496
**ZINC04275810**	ago	-7.264664	-5.763095
**ZINC04277133**	ago	-7.050989	-6.776004
**ZINC04288772**	ago	-6.964926	-5.606238
**ZINC04293333**	ago	-8.364673	-7.604219
**ZINC04294191**	ago	-6.787496	-5.111159
**ZINC04299240**	ago	-7.536103	-6.665292
**ZINC04300899**	ago	-7.867363	-7.906996
**ZINC04302682**	ago	-6.827205	-6.201048
**ZINC04302682**	ago	-6.009477	-6.143509
**ZINC04312943**	ago	-7.998884	-7.236309
**ZINC04316909**	ago	-8.109084	-6.953691
**ZINC04316909**	ago	-6.867962	-5.538581
**ZINC04316910**	ago	-7.986317	-5.92066
**ZINC04316910**	ago	-7.091894	-5.782616
**ZINC04317092**	ago	-7.001095	-7.554158
**ZINC04317092**	ago	-6.633803	-5.918762
**ZINC04317093**	ago	-7.084272	-6.05908
**ZINC04317093**	ago	-6.93598	-5.984154

**ZINC04317099**	ago	-7.170969	-6.909464
**ZINC04317099**	ago	-6.510106	-5.748486
**ZINC04323933**	ago	-6.848083	-5.850249
**ZINC04327825**	ago	-7.842893	-6.537895
**ZINC04333021**	ago	-6.04805	-6.777096
**ZINC04338732**	ago	-6.649974	-6.161785
**ZINC04350837**	ago	-7.220523	-7.313423
**ZINC04350837**	ago	-6.789216	-4.2564
**ZINC04352527**	ago	-6.64006	-6.598046
**ZINC04360485**	ago	-6.18597	-6.235722
**ZINC04364903**	ago	-7.40151	-6.80275
**ZINC04365089**	ago	-6.022561	-5.872441
**ZINC04366879**	ago	-8.659654	-5.484294
**ZINC04367667**	ago	-6.111997	-4.936331
**ZINC04368351**	ago	-7.174438	-6.868989
**ZINC04370986**	ago	-7.997296	-6.812666
**ZINC04380742**	ago	-7.217255	-6.631929
**ZINC04381925**	ago	-7.22973	-5.352501
**ZINC04386723**	ago	-7.353297	-6.248693
**ZINC04386723**	ago	-7.130586	-5.955644
**ZINC04390362**	ago	-6.555492	-5.769439
**ZINC04392005**	ago		-6.712242
**ZINC04405328**	ago	-8.259063	-6.811678
**ZINC04413327**	ago	-8.157934	-6.886869
**ZINC04414705**	ago	-7.145494	-6.713288

**ZINC04416664**	ago	-8.30357	-8.281433
**ZINC04418194**	ago	-7.037193	-6.191664
**ZINC04419755**	ago	-7.101651	-7.308869
**ZINC04420150**	ago	-9.060709	-8.311834
**ZINC04426130**	ago	-7.016441	-7.830752
**ZINC04426137**	ago	-7.804472	-7.257973
**ZINC04437446**	ago	-6.464062	-5.90874
**ZINC04439727**	ago	-7.021519	-4.678266
**ZINC04441320**	ago	-7.565334	-4.346175
**ZINC04441339**	ago	-8.380522	-5.418023
**ZINC04441609**	ago	-9.375406	-7.72961
**ZINC04443288**	ago	-7.340231	-7.280128
**ZINC04443599**	ago	-6.816176	-6.420497
**ZINC04443600**	ago	-6.76678	-5.744796
**ZINC04443785**	ago	-7.409204	-6.972608
**ZINC04444273**	ago	-7.720806	-7.126948
**ZINC04445616**	ago	-6.737845	-5.5333
**ZINC04459057**	ago	-8.191343	-6.384211
**ZINC04460084**	ago	-5.514996	-6.217586
**ZINC04464011**	ago	-7.803905	-5.934978
**ZINC04464518**	ago	-7.293145	-6.869189
**ZINC04464569**	ago	-7.562617	-7.663185
**ZINC04464569**	ago	-7.345173	-7.200932
**ZINC04464598**	ago		-5.396691
**ZINC04465063**	ago	-7.415938	-7.244965

**ZINC04465270**	ago	-7.214116	-3.985923
**ZINC04465458**	ago	-7.517745	-8.320871
**ZINC04465462**	ago	-7.718497	-7.443847
**ZINC04465735**	ago	-8.481144	-4.909836
**ZINC04465874**	ago	-7.390949	-5.952355
**ZINC04465906**	ago	-8.1137	-6.828366
**ZINC04465936**	ago	-6.820447	-5.972873
**ZINC04465937**	ago	-4.87955	-5.959664
**ZINC04465946**	ago	-7.193447	-5.850189
**ZINC04465985**	ago	-7.56409	-5.896372
**ZINC04466091**	ago	-8.326475	-7.073487
**ZINC04466101**	ago	-6.759217	-5.543519
**ZINC04466104**	ago	-6.390215	-4.839246
**ZINC04466167**	ago		-5.763132
**ZINC04467865**	ago	-7.937314	-6.256061
**ZINC04467866**	ago	-7.564535	-3.98502
**ZINC04468290**	ago	-6.548346	-6.448243
**ZINC04469500**	ago	-7.212389	-7.277518
**ZINC04469517**	ago	-8.750547	-8.354338
**ZINC04469584**	ago	-8.259527	-7.098224
**ZINC04469606**	ago	-6.45752	-5.899283
**ZINC04469972**	ago	-8.23005	-4.142635
**ZINC04470117**	ago	-5.658579	-6.259516
**ZINC04470133**	ago	-8.042328	-3.909831
**ZINC04470133**	ago	-7.787851	-3.493548

**ZINC04470253**	ago	-7.46061	-7.063634
**ZINC04470350**	ago	-8.833809	-7.350002
**ZINC04484684**	ago	-7.066028	-5.710949
**ZINC04485868**	ago	-7.180423	-6.869069
**ZINC04488647**	ago	-6.92576	-6.146198
**ZINC04490978**	ago	-6.659921	-6.914743
**ZINC04498807**	ago	-8.023451	-6.442521
**ZINC04501924**	ago	-8.17986	-6.007963
**ZINC04503707**	ago	-6.40734	-3.321363
**ZINC04512252**	ago	-6.179027	-6.180003
**ZINC04528708**	ago	-7.34199	-6.909289
**ZINC04555693**	ago	-7.031577	-4.159176
**ZINC04576942**	ago	-8.361959	-7.15496
**ZINC04594809**	ago	-7.974442	-7.679323
**ZINC04596987**	ago	-6.748041	-5.677761
**ZINC04607984**	ago	-7.026868	-4.797889
**ZINC04622791**	ago	-6.375714	-7.107438
**ZINC04627845**	ago	-7.339709	-6.636822
**ZINC04627846**	ago	-6.759866	-6.018235
**ZINC04627852**	ago	-7.726706	-6.915078
**ZINC04632807**	ago		
**ZINC04633682**	ago	-6.386826	-5.33657
**ZINC04633728**	ago	-7.564805	-4.32914
**ZINC04639931**	ago	-6.762937	-5.67703
**ZINC00579606**	ant		-7.07645

**ZINC00585670**	ant	-5.115321
**ZINC00585720**	ant	-6.735219
**ZINC00588552**	ant	-7.924205
**ZINC00588576**	ant	-6.628933
**ZINC00588611**	ant	-7.266486
**ZINC00595646**	ant	-5.058044
**ZINC00598636**	ant	-7.106281
**ZINC00607149**	ant	-5.268927
**ZINC00629307**	ant	-8.737166
**ZINC00629307**	ant	-5.982702
**ZINC00636729**	ant	-6.284292
**ZINC00637414**	ant	-7.021525
**ZINC00637414**	ant	-6.786709
**ZINC00639578**	ant	-4.686477
**ZINC00643901**	ant	-3.985562
**ZINC00643904**	ant	-4.660546
**ZINC00643907**	ant	-5.580141
**ZINC00643909**	ant	-4.653124
**ZINC00643938**	ant	-5.244546
**ZINC00645028**	ant	-7.522086
**ZINC00645028**	ant	-6.928109
**ZINC00645030**	ant	-5.84215
**ZINC00645030**	ant	-5.763669
**ZINC00647792**	ant	-6.04669
**ZINC00647796**	ant	-6.347741

**ZINC00648339**	ant	-7.007109
**ZINC00648342**	ant	-6.048234
**ZINC00654879**	ant	-6.223487
**ZINC00654882**	ant	-6.232033
**ZINC00657030**	ant	-6.915741
**ZINC00657841**	ant	-6.49595
**ZINC00659015**	ant	-6.397676
**ZINC00659387**	ant	-6.343392
**ZINC00659388**	ant	-5.826344
**ZINC00659610**	ant	-6.697974
**ZINC00659652**	ant	-5.801243
**ZINC00659653**	ant	-7.024225
**ZINC00659700**	ant	-6.271475
**ZINC00661256**	ant	-5.948466
**ZINC00661260**	ant	-2.979651
**ZINC00661531**	ant	-6.190807
**ZINC00661532**	ant	-5.311431
**ZINC00662780**	ant	-6.51188
**ZINC00667560**	ant	-6.303399
**ZINC00667608**	ant	-5.848102
**ZINC00669468**	ant	-5.620224
**ZINC00671896**	ant	-7.464404
**ZINC00673177**	ant	-6.977699
**ZINC00675759**	ant	-6.808097
**ZINC00677351**	ant	-5.774051

**ZINC00679820**	ant		-5.758743
**ZINC00679827**	ant		-5.604949
**ZINC00681062**	ant		-6.271279
**ZINC00681063**	ant		-7.602848
**ZINC00687300**	ant		-6.999054
**ZINC00694392**	ant		-7.009033
**ZINC00702506**	ant		-5.427614
**ZINC00703454**	ant		-8.082281
**ZINC00706428**	ant		-6.671914
**ZINC00707252**	ant		-6.946123
**ZINC00709231**	ant		-7.178563
**ZINC00709231**	ant		-6.981654
**ZINC00709232**	ant		-7.905154
**ZINC00709232**	ant	-6.937209	-7.659618
**ZINC00716648**	ant		-6.013749
**ZINC00717183**	ant	-6.939082	-7.533754
**ZINC00722306**	ant		-5.125538
**ZINC00722329**	ant		-5.14868
**ZINC00722330**	ant		-5.561618
**ZINC00722334**	ant		-5.938863
**ZINC00722337**	ant		-5.826085
**ZINC00723711**	ant	-6.887916	-7.462647
**ZINC00723797**	ant		-7.650878
**ZINC00730070**	ant	-7.271792	-6.641748
**ZINC00730112**	ant		-8.024767

**ZINC00730112**	ant	-7.233394
**ZINC00731175**	ant	-7.048324
**ZINC00731946**	ant	-5.94854
**ZINC00737760**	ant	-6.729881
**ZINC00737779**	ant	-6.502378
**ZINC00737781**	ant	-6.263548
**ZINC00737782**	ant	-6.571787
**ZINC00737783**	ant	-6.760766
**ZINC00737802**	ant	-5.93793
**ZINC00737803**	ant	-5.204877
**ZINC00737804**	ant	-6.107813
**ZINC00737813**	ant	-6.294129
**ZINC00762006**	ant	-5.507695
**ZINC00762033**	ant	-6.245155
**ZINC00762288**	ant	-6.272993
**ZINC00762708**	ant	-6.207728
**ZINC00762810**	ant	-6.287577
**ZINC00762812**	ant	-7.099771
**ZINC00762879**	ant	-5.977999
**ZINC00762914**	ant	-5.140269
**ZINC00763392**	ant	-6.407893
**ZINC00763489**	ant	-4.735117
**ZINC00763754**	ant	-7.090415
**ZINC00763756**	ant	-5.856061
**ZINC00788078**	ant	-6.817992

**ZINC00788081**	ant		-7.243944
**ZINC00801804**	ant		-5.887629
**ZINC00801805**	ant		-5.640903
**ZINC00805569**	ant		-6.535138
**ZINC00834464**	ant		-6.793263
**ZINC00834466**	ant		-6.904385
**ZINC00837059**	ant	-6.982516	-8.043896
**ZINC00838695**	ant		-10.740295
**ZINC00839538**	ant		-7.011498
**ZINC00839544**	ant		-6.703551
**ZINC00848438**	ant		-5.51563
**ZINC00854899**	ant		-6.641786
**ZINC00854899**	ant		-6.432273
**ZINC00862418**	ant		-7.107405
**ZINC00862953**	ant		-4.973526
**ZINC00863826**	ant		-7.590794
**ZINC00863826**	ant		-6.617361
**ZINC00871011**	ant		-7.03556
**ZINC00871127**	ant		-8.215307
**ZINC00871137**	ant		-7.452044
**ZINC00871159**	ant		-7.948134
**ZINC00877372**	ant		-4.709333
**ZINC00888248**	ant		-7.331502
**ZINC00888259**	ant		-6.094822
**ZINC00888261**	ant		-5.796052

**ZINC00892325**	ant		-5.227765
**ZINC00900593**	ant		-8.05083
**ZINC00906284**	ant		-7.88743
**ZINC00920378**	ant		-4.67557
**ZINC00920922**	ant		-4.147651
**ZINC00920924**	ant		-4.287373
**ZINC00920935**	ant		-4.074678
**ZINC00920939**	ant	-4.386008	-4.179598
**ZINC00924743**	ant		-7.771803
**ZINC00924743**	ant		-7.218563
**ZINC00925197**	ant		-8.994371
**ZINC00925365**	ant		-6.386234
**ZINC00925396**	ant		-5.43886
**ZINC00929959**	ant	-7.872016	-6.910949
**ZINC00929959**	ant		-6.627035
**ZINC00945953**	ant		-6.679175
**ZINC00945953**	ant		-6.424811
**ZINC00956922**	ant		-5.376724
**ZINC00956923**	ant		-5.843851
**ZINC00958654**	ant		-6.036668
**ZINC00978417**	ant		-7.670315
**ZINC00989095**	ant		-4.996521
**ZINC00990080**	ant		-6.781293
**ZINC00995087**	ant		-6.243994
**ZINC00995091**	ant		-6.523668

**ZINC00997304**	ant		-6.159145
**ZINC00997310**	ant		-6.202492
**ZINC00998973**	ant		-6.231193
**ZINC00998981**	ant	-6.151896	-6.53756
**ZINC01002507**	ant		-6.257792
**ZINC01002623**	ant		-6.812666
**ZINC01003365**	ant		-6.025047
**ZINC01003367**	ant		-6.540583
**ZINC01003398**	ant		-5.931155
**ZINC01003402**	ant		-6.977465
**ZINC01003414**	ant		-6.073472
**ZINC01005588**	ant		-7.140691
**ZINC01005660**	ant		-6.903046
**ZINC01033154**	ant		-5.923092
**ZINC01044433**	ant		-7.827195
**ZINC01052196**	ant		-6.989592
**ZINC01056072**	ant		-6.694679
**ZINC01056074**	ant		-6.171409
**ZINC01058476**	ant		-6.32742
**ZINC01058691**	ant		-7.053889
**ZINC01058696**	ant		-6.928612
**ZINC01081624**	ant		-5.631024
**ZINC01081668**	ant		-5.366736
**ZINC01083407**	ant		-6.93881
**ZINC01084693**	ant		-7.486456

**ZINC01086230**	ant		-6.188742
**ZINC01095191**	ant		-7.631353
**ZINC01097211**	ant		-6.905343
**ZINC01101162**	ant		-6.040651
**ZINC01101505**	ant		-7.127786
**ZINC01103481**	ant		-5.944941
**ZINC01103482**	ant		-7.605881
**ZINC01103650**	ant		-5.746042
**ZINC01117202**	ant		-7.000278
**ZINC01126371**	ant		-4.595186
**ZINC01131818**	ant		-4.782379
**ZINC01133284**	ant		-7.060625
**ZINC01141419**	ant		-6.71318
**ZINC01144478**	ant		-6.508814
**ZINC01144590**	ant		-6.976619
**ZINC01146927**	ant		-6.166862
**ZINC01146929**	ant		-6.631061
**ZINC01159623**	ant		-8.51784
**ZINC01160167**	ant		-6.659538
**ZINC01162745**	ant		-7.058038
**ZINC01170447**	ant		-5.680729
**ZINC01173893**	ant	-4.587941	-6.626569
**ZINC01174874**	ant		-6.810487
**ZINC01174951**	ant		-6.3147
**ZINC01182837**	ant		-8.260211

**ZINC01194688**	ant		-8.827703
**ZINC01194688**	ant		-7.010812
**ZINC01200339**	ant		-2.797375
**ZINC01205772**	ant		-6.436987
**ZINC01209633**	ant		-5.366264
**ZINC01213119**	ant		-6.279188
**ZINC01214072**	ant		-6.063229
**ZINC01214959**	ant		-4.126739
**ZINC01214961**	ant		-4.389452
**ZINC01216753**	ant		-5.344648
**ZINC01218068**	ant		-6.090906
**ZINC01218901**	ant		-5.373835
**ZINC01229501**	ant		-5.933337
**ZINC01233857**	ant		-7.309575
**ZINC01238144**	ant		-6.490183
**ZINC01238144**	ant		-6.432485
**ZINC01246423**	ant	-6.504662	-7.286066
**ZINC01246423**	ant		-6.72187
**ZINC01247059**	ant		-7.1575
**ZINC01247226**	ant		-8.311177
**ZINC01247226**	ant		-6.584289
**ZINC01288803**	ant		-7.918994
**ZINC01299463**	ant		-7.946701
**ZINC01299463**	ant		-7.446393
**ZINC01317840**	ant		-8.05995

**ZINC01317845**	ant	-7.018039
**ZINC01322728**	ant	-8.498087
**ZINC01322729**	ant	-6.849685
**ZINC01322762**	ant	-6.273672
**ZINC01338009**	ant	-8.06322
**ZINC01339851**	ant	-8.608565
**ZINC01339859**	ant	-8.023895
**ZINC01339882**	ant	-8.832332
**ZINC01341425**	ant	-5.202107
**ZINC01398805**	ant	-3.887713
**ZINC01425970**	ant	-6.689995
**ZINC01428269**	ant	-7.59793
**ZINC01429115**	ant	-6.14567
**ZINC01429206**	ant	-6.47219
**ZINC01434130**	ant	-6.876732
**ZINC01434131**	ant	-7.175454
**ZINC01434887**	ant	-7.789073
**ZINC01435812**	ant	-6.821373
**ZINC01455458**	ant	-6.297196
**ZINC01464592**	ant	-6.675693
**ZINC01464601**	ant	-6.710429
**ZINC01469475**	ant	-8.222585
**ZINC01472102**	ant	-6.772451
**ZINC01472104**	ant	-5.251807
**ZINC01472105**	ant	-5.04276

**ZINC01472929**	ant	-6.465256
**ZINC01476756**	ant	-5.650333
**ZINC01476786**	ant	-6.062271
**ZINC01477319**	ant	-7.565839
**ZINC01482561**	ant	-3.082746
**ZINC01486800**	ant	-7.559071
**ZINC01486801**	ant	-6.879985
**ZINC01486802**	ant	-7.195355
**ZINC01491280**	ant	-7.857808
**ZINC01497277**	ant	-6.279019
**ZINC01497280**	ant	-6.611126
**ZINC01536640**	ant	-6.432551
**ZINC01537121**	ant	-7.923067
**ZINC01538841**	ant	-6.673251
**ZINC01540251**	ant	-6.093861
**ZINC01540485**	ant	-6.29551
**ZINC01540638**	ant	-6.732107
**ZINC01543843**	ant	-11.096177
**ZINC01543910**	ant	-10.183882
**ZINC01543912**	ant	-11.652174
**ZINC01544712**	ant	-6.95846
**ZINC01544713**	ant	-5.393902
**ZINC01545928**	ant	-7.974131
**ZINC01545930**	ant	-7.212404
**ZINC01546089**	ant	-7.595134

**ZINC01546320**	ant		-8.015771
**ZINC01549093**	ant		-7.489766
**ZINC01549256**	ant		-9.337872
**ZINC01549934**	ant	-5.819536	-7.640964
**ZINC01553960**	ant		-10.903387
**ZINC01554005**	ant		-7.723816
**ZINC01554009**	ant		-7.691143
**ZINC01559558**	ant		-8.693508
**ZINC01631690**	ant		-5.240232
**ZINC01729888**	ant		-6.846795
**ZINC01736901**	ant		-6.380716
**ZINC01741527**	ant		-7.826098
**ZINC01760145**	ant		-5.673698
**ZINC01774423**	ant		-6.785595
**ZINC01774425**	ant	-6.071343	-7.156251
**ZINC01774787**	ant		-5.958598
**ZINC01775686**	ant		-6.652373
**ZINC01787221**	ant		
**ZINC01787224**	ant		-6.655778
**ZINC01789867**	ant		-7.273411
**ZINC01796973**	ant		-5.817672
**ZINC01796975**	ant		-5.87966
**ZINC01804415**	ant		-4.539309
**ZINC01808742**	ant		-4.66513
**ZINC01808744**	ant		-6.393273

**ZINC01821402**	ant	-4.136411
**ZINC01821790**	ant	-5.369155
**ZINC01822128**	ant	-7.21554
**ZINC01836749**	ant	-7.670304
**ZINC01849885**	ant	-6.305444
**ZINC01856260**	ant	-5.051873
**ZINC01872494**	ant	-4.929378
**ZINC01881976**	ant	-6.577677
**ZINC01884184**	ant	-4.667299
**ZINC01889545**	ant	-6.291888
**ZINC01890295**	ant	-6.012057
**ZINC01890435**	ant	-6.591884
**ZINC01893139**	ant	-8.175418
**ZINC01893144**	ant	-8.031932
**ZINC01894937**	ant	-4.339875
**ZINC01894940**	ant	-4.259415
**ZINC01894950**	ant	-6.356337
**ZINC01903627**	ant	-5.605571
**ZINC01903631**	ant	-5.425487
**ZINC01903743**	ant	-9.077252
**ZINC01905559**	ant	-4.177052
**ZINC01908345**	ant	-6.627619
**ZINC01910467**	ant	-6.542586
**ZINC01910604**	ant	-6.731407
**ZINC01910607**	ant	-6.714368

**ZINC01912349**	ant	-7.662472
**ZINC01912396**	ant	-5.943082
**ZINC01915724**	ant	-7.974498
**ZINC01915727**	ant	-7.157515
**ZINC01928660**	ant	-5.823302
**ZINC01928661**	ant	-6.003998
**ZINC01928662**	ant	-4.476956
**ZINC01928663**	ant	-5.897974
**ZINC01928664**	ant	-5.549842
**ZINC01928665**	ant	-5.921733
**ZINC01928666**	ant	-5.61824
**ZINC01928667**	ant	-5.863773
**ZINC01928668**	ant	-5.841586
**ZINC01928669**	ant	-5.424752
**ZINC01928672**	ant	-6.175208
**ZINC01928673**	ant	-5.464242
**ZINC01928674**	ant	-5.93532
**ZINC01928675**	ant	-5.241642
**ZINC01928686**	ant	-5.845234
**ZINC01928687**	ant	-5.956936
**ZINC01928688**	ant	-6.130908
**ZINC01928693**	ant	-6.07832
**ZINC01928694**	ant	-5.015099
**ZINC01928695**	ant	-6.078655
**ZINC01928825**	ant	-5.647079

**ZINC01928826**	ant		-5.33822
**ZINC01928883**	ant		-5.588234
**ZINC01928891**	ant		-5.429574
**ZINC01928892**	ant		-4.642375
**ZINC01928917**	ant		-5.698392
**ZINC01929101**	ant		-4.953775
**ZINC01929127**	ant		-5.003468
**ZINC01929182**	ant		-6.180676
**ZINC01929806**	ant		-4.91337
**ZINC01929950**	ant		-5.025185
**ZINC01929951**	ant		-6.05572
**ZINC01930007**	ant		-5.592101
**ZINC01930008**	ant		-4.241647
**ZINC01930010**	ant		-5.672147
**ZINC01930146**	ant		-5.277234
**ZINC01930352**	ant		-4.378931
**ZINC01930353**	ant		-6.195087
**ZINC01930731**	ant		-6.483929
**ZINC01930732**	ant		-6.009113
**ZINC01930733**	ant		-7.347578
**ZINC01930734**	ant		-7.619653
**ZINC01930823**	ant	-4.594547	-6.413529
**ZINC01930824**	ant		-7.226091
**ZINC01930826**	ant		-6.076043
**ZINC01931022**	ant		-4.875443

**ZINC01931023**	ant	-4.157735
**ZINC01931778**	ant	-5.575436
**ZINC01931875**	ant	-6.016207
**ZINC01932062**	ant	-6.027484
**ZINC01932063**	ant	-5.864974
**ZINC01932064**	ant	-4.472338
**ZINC01932065**	ant	-6.213395
**ZINC01932066**	ant	-5.390535
**ZINC01932070**	ant	-5.131685
**ZINC01932081**	ant	-5.132527
**ZINC01932111**	ant	-5.593697
**ZINC01932112**	ant	-5.165739
**ZINC01932113**	ant	-5.608836
**ZINC01932114**	ant	-5.737277
**ZINC01932119**	ant	-5.977024
**ZINC01932120**	ant	-5.073795
**ZINC01932122**	ant	-5.558538
**ZINC01932152**	ant	-5.812328
**ZINC01932153**	ant	-6.091028
**ZINC01932154**	ant	-4.902784
**ZINC01932155**	ant	-5.558933
**ZINC01932156**	ant	-5.272537
**ZINC01932157**	ant	-5.274423
**ZINC01932158**	ant	-5.796182
**ZINC01932212**	ant	-5.59375

**ZINC01933521**	ant	-4.252767
**ZINC01933522**	ant	-5.33281
**ZINC01933526**	ant	-5.240807
**ZINC01933713**	ant	-5.081818
**ZINC01933861**	ant	-6.367406
**ZINC01933864**	ant	-5.571967
**ZINC01935137**	ant	-6.814516
**ZINC01935138**	ant	-6.858221
**ZINC01938174**	ant	-4.971951
**ZINC01938175**	ant	-5.945614
**ZINC01939440**	ant	-6.902977
**ZINC01939441**	ant	-7.795661
**ZINC01942355**	ant	-7.124694
**ZINC01942359**	ant	-6.942028
**ZINC01954393**	ant	-6.206897
**ZINC01954394**	ant	-6.267662
**ZINC01954513**	ant	-5.369823
**ZINC01954528**	ant	-5.369495
**ZINC01954531**	ant	-5.325932
**ZINC01954578**	ant	-6.157042
**ZINC01954582**	ant	-4.919304
**ZINC01954918**	ant	-6.338523
**ZINC01954925**	ant	-4.977074
**ZINC01954927**	ant	-6.325373
**ZINC01954931**	ant	-4.320756

**ZINC01954933**	ant	-4.543094
**ZINC01954946**	ant	-5.814293
**ZINC01954948**	ant	-4.796831
**ZINC01954969**	ant	-5.491813
**ZINC01954986**	ant	-5.49444
**ZINC01954989**	ant	-5.817275
**ZINC01954993**	ant	-5.938199
**ZINC01955046**	ant	-5.634091
**ZINC01967423**	ant	-6.227571
**ZINC01971976**	ant	-6.656649
**ZINC01977304**	ant	-5.434229
**ZINC01984572**	ant	-6.509509
**ZINC01984573**	ant	-6.958669
**ZINC01993110**	ant	-4.305608
**ZINC01993592**	ant	-7.227895
**ZINC01993593**	ant	-7.426789
**ZINC02006239**	ant	-6.757603
**ZINC02007838**	ant	-6.504196
**ZINC02012802**	ant	-5.447834
**ZINC02017896**	ant	-7.123623
**ZINC02027713**	ant	-6.187053
**ZINC02049812**	ant	-8.247386
**ZINC02052846**	ant	-7.538491
**ZINC02059415**	ant	-6.003665
**ZINC02059734**	ant	-6.923265

**ZINC02060332**	ant		-5.399265
**ZINC02060523**	ant		-5.71825
**ZINC02069502**	ant		-6.07237
**ZINC02069503**	ant		-6.236991
**ZINC02069553**	ant		-6.903133
**ZINC02075832**	ant		-5.384966
**ZINC02077287**	ant		-7.154509
**ZINC02079448**	ant		-7.235562
**ZINC02083322**	ant		-4.514163
**ZINC02083324**	ant		-5.256232
**ZINC02083383**	ant		-5.126776
**ZINC02083389**	ant		-4.469592
**ZINC02083424**	ant		-4.56996
**ZINC02086656**	ant		-6.412594
**ZINC02087209**	ant		-6.506099
**ZINC02088235**	ant		-5.61018
**ZINC02089577**	ant		-5.891549
**ZINC02089659**	ant		-5.981633
**ZINC02089928**	ant		-5.987515
**ZINC02090109**	ant	-6.30477	-6.204681
**ZINC02090961**	ant		-6.33447
**ZINC02103495**	ant		-5.960274
**ZINC02103630**	ant		-6.908044
**ZINC02103691**	ant		-6.10771
**ZINC02109382**	ant		-6.489778

**ZINC02109441**	ant		-6.808535
**ZINC02109446**	ant		-6.821329
**ZINC02109562**	ant		-5.834413
**ZINC02109566**	ant		-5.554953
**ZINC02109692**	ant		-6.358266
**ZINC02109712**	ant		-5.379641
**ZINC02109728**	ant		-5.360671
**ZINC02109752**	ant		-5.982021
**ZINC02109766**	ant		-7.222998
**ZINC02109793**	ant		-6.152428
**ZINC02109915**	ant		-6.953499
**ZINC02109919**	ant		-5.848192
**ZINC02112460**	ant		-7.065076
**ZINC02116009**	ant		-7.25117
**ZINC02124825**	ant		-6.484425
**ZINC02124934**	ant		-6.758465
**ZINC02125913**	ant		-8.07064
**ZINC02134453**	ant		-5.465274
**ZINC02134455**	ant		-6.095726
**ZINC02136970**	ant		-7.318062
**ZINC02136982**	ant	-6.352295	-5.908001
**ZINC02149804**	ant		-6.05913
**ZINC02152286**	ant		-6.466923
**ZINC02152514**	ant		-6.488456
**ZINC02152521**	ant		-4.462507

**ZINC02152567**	ant		-4.284861
**ZINC02152596**	ant		-6.207827
**ZINC02152605**	ant	-5.730193	-7.163504
**ZINC02159864**	ant		-5.92476
**ZINC02162437**	ant		-6.82828
**ZINC02162441**	ant		-7.027447
**ZINC02162444**	ant		-7.10505
**ZINC02162549**	ant		-7.018145
**ZINC02165969**	ant		-7.332102
**ZINC02179191**	ant		-6.661889
**ZINC02182535**	ant		-6.52899
**ZINC02182536**	ant		-6.711616
**ZINC02183688**	ant		-6.081722
**ZINC02190536**	ant	-6.088609	-7.282876
**ZINC02194023**	ant		-5.796995
**ZINC02194173**	ant		-6.216173
**ZINC02197338**	ant		-6.474311
**ZINC02198738**	ant		-6.459638
**ZINC02200241**	ant		-7.327433
**ZINC02207866**	ant		-5.986354
**ZINC02210491**	ant		-6.696699
**ZINC02210999**	ant		-4.814878
**ZINC02211285**	ant		-6.716327
**ZINC02220011**	ant		-5.751155
**ZINC02220858**	ant		-5.459712

**ZINC02220860**	ant		-5.226241
**ZINC02220861**	ant		-5.608538
**ZINC02226419**	ant		-6.599705
**ZINC02231040**	ant		-4.985609
**ZINC02233190**	ant		-6.519061
**ZINC02233191**	ant		-6.055805
**ZINC02246943**	ant		-5.873229
**ZINC02247191**	ant		-5.468835
**ZINC02250079**	ant		-6.645543
**ZINC02266184**	ant		-6.27933
**ZINC02267548**	ant		-6.11693
**ZINC02274377**	ant		-7.457982
**ZINC02284317**	ant		-5.991992
**ZINC02285762**	ant		-5.569895
**ZINC02288057**	ant		-7.095599
**ZINC02290889**	ant		-5.800135
**ZINC02291404**	ant		-6.388022
**ZINC02291749**	ant		-5.670397
**ZINC02291752**	ant		-5.795537
**ZINC02292027**	ant		-5.183648
**ZINC02292934**	ant		-6.453359
**ZINC02295415**	ant	-7.463759	-7.611522
**ZINC02295529**	ant		-6.638917
**ZINC02296394**	ant		-7.105774
**ZINC02296827**	ant		-5.732689

**ZINC02298461**	ant	-6.010298
**ZINC02299392**	ant	-5.592655
**ZINC02303062**	ant	-7.278233
**ZINC02306573**	ant	-5.384988
**ZINC02306827**	ant	-6.41755
**ZINC02310973**	ant	-5.525687
**ZINC02312392**	ant	-6.733121
**ZINC02315009**	ant	
**ZINC02315475**	ant	-6.207028
**ZINC02317353**	ant	-6.227959
**ZINC02317498**	ant	-7.527455
**ZINC02317527**	ant	-4.073905
**ZINC02318662**	ant	-6.196204
**ZINC02318663**	ant	-6.044865
**ZINC02323190**	ant	-5.469703
**ZINC02327783**	ant	-7.494426
**ZINC02338464**	ant	-6.958906
**ZINC02339784**	ant	-7.043955
**ZINC02348957**	ant	-5.676966
**ZINC02356453**	ant	-5.64916
**ZINC02359198**	ant	-5.443685
**ZINC02367070**	ant	-5.082238
**ZINC02367071**	ant	-4.802964
**ZINC02368553**	ant	-5.248915
**ZINC02370150**	ant	-5.420817

**ZINC02371801**	ant	-5.473529
**ZINC02373735**	ant	-6.204924
**ZINC02377659**	ant	-7.891755
**ZINC02377700**	ant	-6.374229
**ZINC02377861**	ant	-6.643182
**ZINC02377862**	ant	-6.714837
**ZINC02383490**	ant	-5.702731
**ZINC02383493**	ant	-5.356803
**ZINC02394844**	ant	-5.395753
**ZINC02397054**	ant	-6.78661
**ZINC02397108**	ant	-7.651713
**ZINC02397109**	ant	-7.820185
**ZINC02397814**	ant	-4.750765
**ZINC02409291**	ant	-5.578485
**ZINC02409292**	ant	-5.393909
**ZINC02409851**	ant	-5.464167
**ZINC02415267**	ant	-5.650271
**ZINC02424040**	ant	-5.544942
**ZINC02427884**	ant	-7.636897
**ZINC02427886**	ant	-6.057873
**ZINC02427937**	ant	-6.741767
**ZINC02438186**	ant	-6.071822
**ZINC02442745**	ant	-6.690322
**ZINC02443141**	ant	-6.130294
**ZINC02443142**	ant	-6.353016

**ZINC02445244**	ant		-6.192808
**ZINC02447268**	ant		-7.85488
**ZINC02450667**	ant		-4.675944
**ZINC02459690**	ant		-6.590502
**ZINC02459712**	ant		-5.826663
**ZINC02459798**	ant		-4.671589
**ZINC02459811**	ant		-5.728584
**ZINC02460045**	ant		-6.630727
**ZINC02460285**	ant		-6.064861
**ZINC02460680**	ant		-4.838765
**ZINC02465216**	ant		-6.534233
**ZINC02467965**	ant		-6.336713
**ZINC02468071**	ant		-4.226218
**ZINC02469334**	ant		-6.63674
**ZINC02474197**	ant		-5.128623
**ZINC02474233**	ant		-4.176882
**ZINC02474883**	ant		-4.842893
**ZINC02474897**	ant		-5.124713
**ZINC02475780**	ant		-5.825925
**ZINC02480526**	ant		-7.882847
**ZINC02480760**	ant		-6.94453
**ZINC02483368**	ant		-5.510307
**ZINC02483449**	ant		-5.471325
**ZINC02500923**	ant		-5.885386
**ZINC02516263**	ant	-6.177778	-6.765675

**ZINC02520447**	ant		-7.628349
**ZINC02528511**	ant		-8.438736
**ZINC02530240**	ant		-6.595666
**ZINC02556565**	ant		-6.923385
**ZINC02565251**	ant		-7.385798
**ZINC02586510**	ant		-6.418118
**ZINC02586513**	ant		-6.048139
**ZINC02608643**	ant		-5.789357
**ZINC02611768**	ant		-6.732317
**ZINC02611773**	ant		-6.91775
**ZINC02611774**	ant		-6.270268
**ZINC02617440**	ant		-6.796591
**ZINC02618809**	ant		-6.558906
**ZINC02620958**	ant		-5.060664
**ZINC02622045**	ant		-6.519024
**ZINC02623394**	ant		-6.835707
**ZINC02623427**	ant	-8.894898	-6.896483
**ZINC02626267**	ant		-6.528643
**ZINC02630310**	ant		-6.925996
**ZINC02630379**	ant		-7.41639
**ZINC02631312**	ant		-7.766391
**ZINC02631313**	ant		-7.62722
**ZINC02633482**	ant		-6.178063
**ZINC02635811**	ant		-7.452068
**ZINC02639735**	ant		-7.534555

**ZINC02652402**	ant		-5.292557
**ZINC02655013**	ant		-7.810295
**ZINC02655413**	ant		-6.812889
**ZINC02655414**	ant		-8.07788
**ZINC02656792**	ant	-8.311022	-6.977014
**ZINC02660742**	ant		-7.216451
**ZINC02660743**	ant		-6.677895
**ZINC02662531**	ant		-4.991285
**ZINC02662532**	ant		-4.891928
**ZINC02666224**	ant		-6.364474
**ZINC02668059**	ant		-5.558466
**ZINC02677465**	ant		-7.373737
**ZINC02677488**	ant		-5.677149
**ZINC02678808**	ant		-8.061089
**ZINC02693530**	ant		-6.834427
**ZINC02693606**	ant		-6.538621
**ZINC02694220**	ant		-6.657001
**ZINC02708613**	ant		-4.279353
**ZINC02729925**	ant		-7.043847
**ZINC02730312**	ant		-8.558412
**ZINC02730507**	ant		-5.896111
**ZINC02736205**	ant		-5.987975
**ZINC02736952**	ant		-6.013352
**ZINC02738197**	ant		-6.779516
**ZINC02740689**	ant		-7.50642

**ZINC02741361**	ant		-5.800715
**ZINC02741884**	ant		-5.460826
**ZINC02748459**	ant		-6.117712
**ZINC02750689**	ant		-6.180113
**ZINC02750855**	ant		-7.000471
**ZINC02751256**	ant		-7.128521
**ZINC02752136**	ant		-6.529535
**ZINC02753343**	ant		-5.196901
**ZINC02754861**	ant	-6.716498	-6.125665
**ZINC02754974**	ant		-5.919416
**ZINC02758658**	ant		-7.541718
**ZINC02759326**	ant		-6.281698
**ZINC02760245**	ant		-3.715232
**ZINC02761727**	ant		-6.526683
**ZINC02762418**	ant		-5.866986
**ZINC02768053**	ant		-6.220576
**ZINC02769040**	ant		-6.757988
**ZINC02769041**	ant		-6.197917
**ZINC02769108**	ant		-7.301814
**ZINC02785256**	ant		-7.194109
**ZINC02785395**	ant		-6.43812
**ZINC02785483**	ant		-7.052011
**ZINC02807559**	ant		-6.579221
**ZINC02814820**	ant		-7.45389
**ZINC02815650**	ant		-6.599995

**ZINC02818172**	ant	-6.413863
**ZINC02819524**	ant	
**ZINC02826408**	ant	-6.342906
**ZINC02837145**	ant	-6.531915
**ZINC02839065**	ant	-5.868986
**ZINC02841253**	ant	-5.570978
**ZINC02851494**	ant	-6.473522
**ZINC02867724**	ant	-7.62075
**ZINC02867866**	ant	-6.770106
**ZINC02876242**	ant	-6.674302
**ZINC02876954**	ant	-6.546009
**ZINC02876955**	ant	-6.036948
**ZINC02877109**	ant	-6.505338
**ZINC02884958**	ant	-6.779629
**ZINC02886214**	ant	-6.611388
**ZINC02892505**	ant	-5.743387
**ZINC02895727**	ant	-6.875903
**ZINC02895729**	ant	-7.814946
**ZINC02897718**	ant	-7.294917
**ZINC02897720**	ant	-6.875222
**ZINC02898226**	ant	-7.246916
**ZINC02898228**	ant	-6.980534
**ZINC02899235**	ant	-7.232261
**ZINC02900261**	ant	-6.724936
**ZINC02904782**	ant	-4.423252

**ZINC02904844**	ant	-4.677646
**ZINC02910571**	ant	-6.046925
**ZINC02910650**	ant	-6.475339
**ZINC02917867**	ant	-6.134359
**ZINC02928837**	ant	-6.057796
**ZINC02928920**	ant	-7.267978
**ZINC02930797**	ant	-7.675824
**ZINC02932987**	ant	-7.275718
**ZINC02935971**	ant	-7.507818
**ZINC02935974**	ant	-7.770536
**ZINC02939682**	ant	-6.985925
**ZINC02941413**	ant	-6.011593
**ZINC02942926**	ant	-5.166652
**ZINC02943056**	ant	
**ZINC02944611**	ant	-6.456778
**ZINC02952852**	ant	-6.57699
**ZINC02956172**	ant	-5.664915
**ZINC02962417**	ant	-5.233049
**ZINC02979111**	ant	-8.031973
**ZINC02979568**	ant	-8.062433
**ZINC02979631**	ant	-6.584742
**ZINC02984230**	ant	-6.780527
**ZINC02984995**	ant	-4.724732
**ZINC02985062**	ant	-7.11446
**ZINC02989800**	ant	-6.696102

**ZINC02990618**	ant		-8.137285
**ZINC02995771**	ant		-6.608255
**ZINC02995832**	ant		-7.398774
**ZINC02998347**	ant		-5.447924
**ZINC03002535**	ant		-5.222491
**ZINC03002662**	ant		-6.454279
**ZINC03002663**	ant		-6.187179
**ZINC03007999**	ant		-5.572776
**ZINC03008019**	ant		-6.060965
**ZINC03009731**	ant		-4.250783
**ZINC03009860**	ant		
**ZINC03026191**	ant		-7.157876
**ZINC03027356**	ant		-5.543588
**ZINC03033244**	ant		-7.552076
**ZINC03033245**	ant		-7.56638
**ZINC03037529**	ant		-6.055452
**ZINC03037530**	ant		-5.671731
**ZINC03040157**	ant		-5.113858
**ZINC03040549**	ant		-5.569008
**ZINC03040758**	ant	-6.976964	-5.276496
**ZINC03040819**	ant		-5.237111
**ZINC03044938**	ant		-5.779618
**ZINC03045000**	ant		-5.967703
**ZINC03045016**	ant		-5.781627
**ZINC03046026**	ant		-5.883625

**ZINC03046894**	ant		-5.420544
**ZINC03046896**	ant		-5.832227
**ZINC03047605**	ant		-5.50042
**ZINC03047614**	ant		-5.247179
**ZINC03050276**	ant		-6.434757
**ZINC03064452**	ant		-6.505492
**ZINC03064472**	ant		-6.015953
**ZINC03064478**	ant		-6.138049
**ZINC03065467**	ant		-4.552165
**ZINC03079492**	ant		-6.91783
**ZINC03080486**	ant		-7.487239
**ZINC03089144**	ant		-6.042203
**ZINC03101100**	ant		-7.678153
**ZINC03142280**	ant		-5.275516
**ZINC03147006**	ant		-3.024102
**ZINC03147042**	ant		-7.370787
**ZINC03162711**	ant		-7.269297
**ZINC03201370**	ant		-8.56384
**ZINC03208795**	ant		-5.935883
**ZINC03216374**	ant		-7.843595
**ZINC03216375**	ant		-7.419881
**ZINC03216401**	ant	-5.685054	-6.788145
**ZINC03217067**	ant		-7.934185
**ZINC03217074**	ant		-6.847728
**ZINC03217081**	ant		-7.867723

**ZINC03217082**	ant		-8.25988
**ZINC03217298**	ant		-7.985453
**ZINC03217615**	ant		-7.539368
**ZINC03217622**	ant		-6.594481
**ZINC03217623**	ant		-7.900728
**ZINC03218208**	ant		-7.84961
**ZINC03219277**	ant		-8.178235
**ZINC03219376**	ant		-8.151244
**ZINC03221218**	ant		-6.688425
**ZINC03222183**	ant		-5.707284
**ZINC03222184**	ant		-5.23899
**ZINC03222926**	ant		-6.854668
**ZINC03226657**	ant		-7.982129
**ZINC03226806**	ant		-7.086078
**ZINC03230144**	ant		-7.96185
**ZINC03235568**	ant		-7.68672
**ZINC03236999**	ant		-8.175808
**ZINC03237119**	ant		-7.837884
**ZINC03237120**	ant	-5.271532	-6.921186
**ZINC03241493**	ant		-7.562322
**ZINC03244845**	ant		-8.197374
**ZINC03247329**	ant		-6.955624
**ZINC03250623**	ant		-7.484208
**ZINC03253744**	ant		-6.961138
**ZINC03260869**	ant		-4.528298

**ZINC03263459**	ant		-7.73375
**ZINC03266214**	ant	-5.989015	-7.453534
**ZINC03266774**	ant		-7.84934
**ZINC03267033**	ant		-8.101059
**ZINC03271407**	ant		-6.197829
**ZINC03273346**	ant		-7.670832
**ZINC03273347**	ant		-7.242634
**ZINC03276105**	ant		-6.57663
**ZINC03276246**	ant		-7.218022
**ZINC03277223**	ant		-7.831061
**ZINC03279100**	ant		-5.184786
**ZINC03281528**	ant		-8.361058
**ZINC03288679**	ant	-6.454198	-9.02889
**ZINC03288681**	ant		-8.883805
**ZINC03292352**	ant		-6.06664
**ZINC03297299**	ant		-7.247096
**ZINC03303998**	ant		-5.783676
**ZINC03304833**	ant		-7.189913
**ZINC03309213**	ant		-5.812397
**ZINC03309215**	ant	-3.758197	-5.416927
**ZINC03310147**	ant		-7.307885
**ZINC03311238**	ant		-6.906293
**ZINC03314294**	ant		-7.838831
**ZINC03317495**	ant		-4.249321
**ZINC03317533**	ant		-6.751289

**ZINC03319194**	ant		-6.701839
**ZINC03323944**	ant		-7.996225
**ZINC03328866**	ant	-5.635834	-3.21777
**ZINC03337196**	ant		-4.206566
**ZINC03337198**	ant		-4.201857
**ZINC03338010**	ant		-6.032459
**ZINC03338015**	ant		-7.047865
**ZINC03338016**	ant		-5.900043
**ZINC03339738**	ant		-6.041601
**ZINC03339739**	ant		-6.543281
**ZINC03344064**	ant	-4.820629	-7.123102
**ZINC03344582**	ant		-4.652906
**ZINC03349271**	ant		-7.528028
**ZINC03349272**	ant		-6.191311
**ZINC03355881**	ant		-7.604419
**ZINC03360767**	ant		-8.29906
**ZINC03370987**	ant		-6.479986
**ZINC03372386**	ant	-4.94058	-6.511076
**ZINC03376147**	ant		-6.504295
**ZINC03383264**	ant	-5.837501	-7.865325
**ZINC03396791**	ant	-9.420522	-7.015511
**ZINC03396794**	ant		-6.12747
**ZINC03399073**	ant		-5.56894
**ZINC03412342**	ant		-8.099268
**ZINC03412345**	ant		-8.669732

**ZINC03414251**	ant		-7.382033
**ZINC03427748**	ant		-6.253633
**ZINC03427755**	ant		-7.071949
**ZINC03430351**	ant	-6.099736	-6.113429
**ZINC03433146**	ant		-7.37768
**ZINC03451956**	ant		-6.887157
**ZINC03458214**	ant		-6.615557
**ZINC03581683**	ant	-7.160628	-7.688413
**ZINC03581695**	ant		-7.699447
**ZINC03584963**	ant		-6.076769
**ZINC03584989**	ant		-6.368687
**ZINC03585007**	ant		-6.043762
**ZINC03610404**	ant		-8.501187
**ZINC03615661**	ant		-6.076115
**ZINC03615669**	ant		-6.081284
**ZINC03617120**	ant		-7.296923
**ZINC03624949**	ant		-6.159037
**ZINC03632166**	ant		-4.341928
**ZINC03638881**	ant		-7.81533
**ZINC03648187**	ant	-8.52057	-6.649177
**ZINC03659083**	ant		-7.05316
**ZINC03663323**	ant	-4.175119	-6.091726
**ZINC03663324**	ant		-6.720227
**ZINC03663748**	ant		-6.202042
**ZINC03664829**	ant		-6.693986

**ZINC03673877**	ant		-5.732232
**ZINC03680809**	ant		-6.194104
**ZINC03702266**	ant	-6.036733	-6.328859
**ZINC03702267**	ant		-7.512374
**ZINC03702398**	ant		-6.342354
**ZINC03702399**	ant		-7.642234
**ZINC03702466**	ant	-7.838219	-6.164642
**ZINC03702467**	ant		-6.589468
**ZINC03703747**	ant		-6.258387
**ZINC03707552**	ant		-7.777977
**ZINC03735201**	ant		-8.52619
**ZINC03735202**	ant		-6.626905
**ZINC03735203**	ant		-8.298851
**ZINC03735204**	ant		-6.920698
**ZINC03735275**	ant		-7.758004
**ZINC03735429**	ant		-7.658308
**ZINC03735430**	ant		-6.946849
**ZINC03735449**	ant		-8.439814
**ZINC03735450**	ant		-6.776634
**ZINC03736098**	ant		-8.345082
**ZINC03736099**	ant		-7.224929
**ZINC03736118**	ant		-7.944446
**ZINC03736119**	ant		
**ZINC03736289**	ant		-7.804493
**ZINC03736693**	ant		-8.106092

**ZINC03736694**	ant	-6.678271
**ZINC03737042**	ant	-6.813029
**ZINC03737272**	ant	-8.117203
**ZINC03737273**	ant	-7.422432
**ZINC03737292**	ant	-8.011311
**ZINC03737832**	ant	-7.430633
**ZINC03737833**	ant	-6.452036
**ZINC03737853**	ant	-8.06022
**ZINC03737872**	ant	-7.577233
**ZINC03737873**	ant	-6.473778
**ZINC03737912**	ant	-7.906776
**ZINC03737913**	ant	-6.719346
**ZINC03738090**	ant	-7.703911
**ZINC03738091**	ant	-6.384656
**ZINC03738110**	ant	-7.985667
**ZINC03738250**	ant	-7.329764
**ZINC03738251**	ant	-7.171538
**ZINC03738274**	ant	-8.050436
**ZINC03738275**	ant	-7.734651
**ZINC03738276**	ant	-8.567985
**ZINC03738277**	ant	-6.974979
**ZINC03738279**	ant	-7.553046
**ZINC03738289**	ant	-6.815816
**ZINC03738298**	ant	-8.063977
**ZINC03738299**	ant	

**ZINC03738370**	ant	-7.783954
**ZINC03738371**	ant	-6.976554
**ZINC03738418**	ant	-8.349495
**ZINC03738419**	ant	-7.278327
**ZINC03738927**	ant	-6.105816
**ZINC03738928**	ant	-6.671718
**ZINC03738957**	ant	-7.638008
**ZINC03738958**	ant	-7.063702
**ZINC03738977**	ant	-7.624115
**ZINC03738978**	ant	-6.677469
**ZINC03739070**	ant	-7.96145
**ZINC03739071**	ant	-5.985935
**ZINC03764944**	ant	-5.498715
**ZINC03765538**	ant	-7.423598
**ZINC03765581**	ant	-7.158356
**ZINC03765582**	ant	-6.068416
**ZINC03767970**	ant	-6.222578
**ZINC03779767**	ant	-7.542692
**ZINC03779771**	ant	-7.819524
**ZINC03786823**	ant	-7.689444
**ZINC03786825**	ant	-7.943306
**ZINC03787638**	ant	
**ZINC03788784**	ant	-6.953561
**ZINC03792650**	ant	-8.138736
**ZINC03798280**	ant	-7.534212

**ZINC03809371**	ant		-7.657346
**ZINC03826688**	ant		-11.778523
**ZINC03826690**	ant		-11.690236
**ZINC03827594**	ant		-7.475161
**ZINC03830716**	ant		-7.037807
**ZINC03846127**	ant		-6.723698
**ZINC03849572**	ant		-6.496328
**ZINC03849572**	ant		-5.934128
**ZINC03849575**	ant		-6.644927
**ZINC03849575**	ant		-6.107437
**ZINC03849912**	ant		-6.522219
**ZINC03850235**	ant		-5.373455
**ZINC03851130**	ant		-5.960342
**ZINC03851483**	ant		-5.636654
**ZINC03851492**	ant		-8.214986
**ZINC03851498**	ant		-6.579213
**ZINC03851630**	ant		-8.215573
**ZINC03851630**	ant		-7.713472
**ZINC03852289**	ant		-5.736799
**ZINC03852293**	ant		-6.794422
**ZINC03852300**	ant		-7.589278
**ZINC03852557**	ant		-7.373399
**ZINC03852558**	ant		-8.210746
**ZINC03852637**	ant	-4.713868	-7.781539
**ZINC03852637**	ant		-7.355195

**ZINC03852647**	ant	-8.000468
**ZINC03852647**	ant	-7.135314
**ZINC03852884**	ant	-6.455303
**ZINC03853209**	ant	-5.856018
**ZINC03853433**	ant	-5.718614
**ZINC03853434**	ant	-6.204356
**ZINC03853435**	ant	-6.668087
**ZINC03853436**	ant	-5.985005
**ZINC03853438**	ant	-2.471447
**ZINC03854241**	ant	-6.881167
**ZINC03854241**	ant	-4.419109
**ZINC03854242**	ant	-7.43238
**ZINC03854242**	ant	-7.081331
**ZINC03854738**	ant	-6.627055
**ZINC03854740**	ant	-6.403028
**ZINC03854788**	ant	-5.989988
**ZINC03854886**	ant	-6.693088
**ZINC03854887**	ant	-6.915643
**ZINC03854890**	ant	
**ZINC03854891**	ant	-7.057819
**ZINC03854892**	ant	-6.619672
**ZINC03854908**	ant	-5.426977
**ZINC03854909**	ant	-3.69541
**ZINC03854910**	ant	-5.652795
**ZINC03855159**	ant	-5.805665

**ZINC03855159**	ant		-5.776635
**ZINC03856269**	ant		
**ZINC03856270**	ant		
**ZINC03856334**	ant	-4.836631	-6.209585
**ZINC03856372**	ant		-7.079046
**ZINC03856396**	ant		-6.844603
**ZINC03856424**	ant		-6.518053
**ZINC03857170**	ant		-3.982073
**ZINC03857180**	ant		-7.412989
**ZINC03857180**	ant		-6.641936
**ZINC03857246**	ant		-7.28259
**ZINC03857381**	ant		-4.549217
**ZINC03858140**	ant		-4.926495
**ZINC03858148**	ant	-4.680492	-6.715841
**ZINC03858149**	ant		-7.432499
**ZINC03858187**	ant		-5.253975
**ZINC03858223**	ant		-5.041402
**ZINC03858225**	ant		-5.852591
**ZINC03858226**	ant		-5.455297
**ZINC03858284**	ant		-5.421374
**ZINC03858286**	ant		-4.950442
**ZINC03858337**	ant		-5.082484
**ZINC03858338**	ant		
**ZINC03858346**	ant		-5.857424
**ZINC03858348**	ant		-4.828802

**ZINC03858426**	ant	-4.53164
**ZINC03858521**	ant	-5.347097
**ZINC03858567**	ant	-6.581564
**ZINC03858568**	ant	-6.120102
**ZINC03858573**	ant	-7.439678
**ZINC03858574**	ant	-5.718009
**ZINC03858833**	ant	-6.970182
**ZINC03858929**	ant	-6.620416
**ZINC03858978**	ant	-4.891757
**ZINC03859070**	ant	-4.642488
**ZINC03859172**	ant	-6.266768
**ZINC03859190**	ant	-4.89529
**ZINC03859204**	ant	-5.677909
**ZINC03859360**	ant	-5.083925
**ZINC03859961**	ant	-8.912628
**ZINC03859961**	ant	-8.24583
**ZINC03859963**	ant	-6.240249
**ZINC03860100**	ant	-3.79687
**ZINC03860101**	ant	-7.008891
**ZINC03861442**	ant	-5.583943
**ZINC03864127**	ant	-4.666438
**ZINC03864324**	ant	
**ZINC03866732**	ant	-8.232254
**ZINC03866732**	ant	-8.059533
**ZINC03866783**	ant	-6.418152

**ZINC03866893**	ant		-5.494675
**ZINC03867025**	ant		-7.121729
**ZINC03867304**	ant		-7.606073
**ZINC03867304**	ant		-6.67589
**ZINC03867360**	ant		-7.972715
**ZINC03867360**	ant		-7.517976
**ZINC03867427**	ant		-7.136235
**ZINC03867456**	ant		-9.011305
**ZINC03867456**	ant		-8.030819
**ZINC03867590**	ant		-4.30662
**ZINC03867591**	ant		-5.767675
**ZINC03867657**	ant		-5.581535
**ZINC03868358**	ant	-6.445675	-6.118815
**ZINC03868469**	ant		-5.629636
**ZINC03868470**	ant		-6.810261
**ZINC03868508**	ant		-5.777055
**ZINC03868632**	ant		-7.525458
**ZINC03868639**	ant		-6.974104
**ZINC03868704**	ant		-7.041167
**ZINC03868767**	ant		-6.555607
**ZINC03868802**	ant		-7.413075
**ZINC03868803**	ant		-7.094563
**ZINC03868804**	ant		-6.280119
**ZINC03869129**	ant		-5.399915
**ZINC03869130**	ant		-4.446914

**ZINC03873507**	ant	-7.874254
**ZINC03873537**	ant	-7.721399
**ZINC03873737**	ant	-6.55583
**ZINC03873738**	ant	-6.796293
**ZINC03889310**	ant	-6.888091
**ZINC03889319**	ant	-6.545293
**ZINC03889321**	ant	-7.338234
**ZINC03889369**	ant	-6.797182
**ZINC03889371**	ant	-6.404219
**ZINC03889724**	ant	-6.820271
**ZINC03890154**	ant	-6.718703
**ZINC03890157**	ant	-4.397052
**ZINC03890426**	ant	-5.01279
**ZINC03890673**	ant	-4.881233
**ZINC03890770**	ant	-6.163082
**ZINC03890885**	ant	-7.372219
**ZINC03891148**	ant	-6.781783
**ZINC03892155**	ant	-7.356387
**ZINC03892827**	ant	-7.124116
**ZINC03892835**	ant	-5.103482
**ZINC03892893**	ant	-5.657296
**ZINC03892894**	ant	-5.143404
**ZINC03892915**	ant	-6.875689
**ZINC03916788**	ant	-9.957393
**ZINC03917999**	ant	-7.871531

**ZINC03917999**	ant	-6.793735
**ZINC03918428**	ant	-11.359365
**ZINC03921526**	ant	-7.241789
**ZINC03921605**	ant	-7.96511
**ZINC03921609**	ant	-7.736689
**ZINC03922638**	ant	-7.983699
**ZINC03922638**	ant	-7.495162
**ZINC03923434**	ant	-7.971824
**ZINC03923434**	ant	-7.868062
**ZINC03923441**	ant	-8.485236
**ZINC03923441**	ant	-7.365231
**ZINC03923441**	ant	-7.213619
**ZINC03927703**	ant	-8.197448
**ZINC03927703**	ant	-7.388403
**ZINC03928829**	ant	-6.230959
**ZINC03931554**	ant	-7.984294
**ZINC03933689**	ant	-6.251387
**ZINC03937338**	ant	-7.004389
**ZINC03937476**	ant	-6.082406
**ZINC03938009**	ant	-6.115443
**ZINC03938556**	ant	-8.333429
**ZINC03938562**	ant	-7.809741
**ZINC03938568**	ant	-8.822591
**ZINC03940865**	ant	-8.270987
**ZINC03941026**	ant	-8.887552

**ZINC03942856**	ant		-8.714789
**ZINC03949966**	ant		-8.455829
**ZINC03959850**	ant		-8.323029
**ZINC03962129**	ant		-5.606356
**ZINC03962342**	ant	-8.890851	-8.138385
**ZINC03964711**	ant		-7.387889
**ZINC03965077**	ant		-11.708416
**ZINC03967879**	ant		-8.213425
**ZINC03967879**	ant		-6.920331
**ZINC03967928**	ant		-7.484136
**ZINC03967928**	ant		-6.166933
**ZINC03968422**	ant		-5.604954
**ZINC03968454**	ant		-5.89922
**ZINC03968455**	ant		-5.805006
**ZINC03968457**	ant		-5.779884
**ZINC03968458**	ant		-6.021107
**ZINC03968459**	ant		-5.783167
**ZINC03968493**	ant		-7.179383
**ZINC03968564**	ant		-7.591074
**ZINC03968603**	ant		-5.48341
**ZINC03968634**	ant		-6.06396
**ZINC03968638**	ant		-5.697942
**ZINC03968639**	ant		-5.929666
**ZINC03968649**	ant		-5.952641
**ZINC03968669**	ant		-7.789145

**ZINC03968686**	ant	-6.416233
**ZINC03968687**	ant	-5.808614
**ZINC03968688**	ant	-6.589386
**ZINC03968689**	ant	-5.788845
**ZINC03968767**	ant	-6.216509
**ZINC03968993**	ant	-5.854546
**ZINC03969081**	ant	-6.323283
**ZINC03969168**	ant	-7.185862
**ZINC03969203**	ant	
**ZINC03969229**	ant	-6.20019
**ZINC03969708**	ant	-7.106784
**ZINC03969708**	ant	-6.69102
**ZINC03969723**	ant	-7.161097
**ZINC03969756**	ant	-6.537231
**ZINC03969757**	ant	-5.452259
**ZINC03969972**	ant	-7.627698
**ZINC03969973**	ant	-6.954637
**ZINC03969974**	ant	-7.527813
**ZINC03970364**	ant	-7.026027
**ZINC03970367**	ant	-5.632283
**ZINC03970447**	ant	-7.230348
**ZINC03970455**	ant	-4.974566
**ZINC03970586**	ant	-5.780655
**ZINC03970592**	ant	-6.145234
**ZINC03970593**	ant	-7.918099

**ZINC03970595**	ant	-6.412119	-6.486779
**ZINC03970603**	ant		-7.2766
**ZINC03970659**	ant		-7.438525
**ZINC03970660**	ant		-7.106074
**ZINC03970661**	ant		-5.112882
**ZINC03970662**	ant		-5.059245
**ZINC03970799**	ant		-4.012841
**ZINC03970800**	ant		-4.351289
**ZINC03970802**	ant		-5.370888
**ZINC03970816**	ant		-7.539528
**ZINC03970887**	ant		-4.285767
**ZINC03970936**	ant		-6.735907
**ZINC03970937**	ant		-5.871337
**ZINC03971056**	ant		-5.67293
**ZINC03971603**	ant		-5.908739
**ZINC03971661**	ant		-6.807294
**ZINC03971664**	ant		-5.713202
**ZINC03971797**	ant		-7.180827
**ZINC03971967**	ant		-5.415209
**ZINC03971969**	ant		-4.322207
**ZINC03972098**	ant		-5.223125
**ZINC03972309**	ant		-5.422851
**ZINC03972313**	ant		-5.839585
**ZINC03972329**	ant		-5.222388
**ZINC03972331**	ant		-5.180564

**ZINC03972360**	ant	-4.034804
**ZINC03972387**	ant	-5.526093
**ZINC03972392**	ant	-5.576017
**ZINC03972594**	ant	-6.645307
**ZINC03972600**	ant	-4.797948
**ZINC03972604**	ant	-5.644571
**ZINC03972613**	ant	-6.006623
**ZINC03972622**	ant	-5.079671
**ZINC03972640**	ant	-5.469964
**ZINC03972681**	ant	-5.549819
**ZINC03972695**	ant	-6.47354
**ZINC03972701**	ant	-6.185725
**ZINC03972759**	ant	-4.204978
**ZINC03972763**	ant	-5.376906
**ZINC03972801**	ant	-3.264925
**ZINC03972807**	ant	-5.669879
**ZINC03972845**	ant	-7.274068
**ZINC03972894**	ant	-5.35616
**ZINC03972897**	ant	-7.634173
**ZINC03973402**	ant	-6.194164
**ZINC03973415**	ant	-6.391755
**ZINC03973621**	ant	-5.749029
**ZINC03973640**	ant	-5.954182
**ZINC03973641**	ant	-6.216582
**ZINC03973644**	ant	-6.121362

**ZINC03973645**	ant	-6.120353
**ZINC03973648**	ant	-6.21089
**ZINC03973661**	ant	-6.176839
**ZINC03973662**	ant	-5.923835
**ZINC03973665**	ant	-5.981798
**ZINC03973670**	ant	-5.362059
**ZINC03973672**	ant	-6.098622
**ZINC03973683**	ant	-6.08863
**ZINC03973712**	ant	-5.413178
**ZINC03973713**	ant	-6.170344
**ZINC03973716**	ant	-6.304339
**ZINC03973720**	ant	-5.745114
**ZINC03973724**	ant	-6.053318
**ZINC03973733**	ant	-6.16358
**ZINC03973766**	ant	-6.704709
**ZINC03973891**	ant	-7.595953
**ZINC03973916**	ant	-7.478517
**ZINC03973972**	ant	-6.512461
**ZINC03975032**	ant	-6.348433
**ZINC03975426**	ant	-7.485796
**ZINC03975462**	ant	-5.866492
**ZINC03975465**	ant	-5.93802
**ZINC03975986**	ant	-3.88713
**ZINC03976059**	ant	-6.117939
**ZINC03976063**	ant	-6.594115

**ZINC03976081**	ant	-4.958042
**ZINC03976086**	ant	-5.381272
**ZINC03976091**	ant	-6.66892
**ZINC03976107**	ant	-4.923651
**ZINC03976261**	ant	-3.960501
**ZINC03976754**	ant	-5.364611
**ZINC03976755**	ant	-7.870041
**ZINC03976776**	ant	-6.740557
**ZINC03976778**	ant	-6.655897
**ZINC03976784**	ant	-6.262458
**ZINC03976785**	ant	-7.383232
**ZINC03976787**	ant	-6.941701
**ZINC03976788**	ant	-6.80962
**ZINC03976789**	ant	-6.698299
**ZINC03976791**	ant	-6.514213
**ZINC03976792**	ant	-6.277154
**ZINC03976793**	ant	-6.345677
**ZINC03976795**	ant	-7.2956
**ZINC03977490**	ant	-7.284911
**ZINC03977586**	ant	-6.482029
**ZINC03979729**	ant	-7.214998
**ZINC03980004**	ant	-7.861498
**ZINC03980004**	ant	-6.713438
**ZINC03980007**	ant	-7.18198
**ZINC03980007**	ant	-6.722105

**ZINC03980034**	ant	-7.118094
**ZINC03980034**	ant	-7.082953
**ZINC03980675**	ant	-6.990123
**ZINC03980805**	ant	-7.865041
**ZINC03980806**	ant	-7.869526
**ZINC03981917**	ant	-7.277253
**ZINC03982877**	ant	-5.321487
**ZINC03983031**	ant	-7.000362
**ZINC03983331**	ant	-3.875327
**ZINC03984508**	ant	-7.188372
**ZINC03984510**	ant	-6.181853
**ZINC03985801**	ant	-9.251345
**ZINC03987265**	ant	-8.397114
**ZINC03987272**	ant	-8.13258
**ZINC03987533**	ant	-5.803037
**ZINC03987681**	ant	-6.811888
**ZINC03987692**	ant	-6.936276
**ZINC03990910**	ant	-7.88659
**ZINC03991317**	ant	-6.368665
**ZINC03991381**	ant	-6.797199
**ZINC03991708**	ant	-8.903226
**ZINC03991963**	ant	-6.407939
**ZINC03992380**	ant	-7.002222
**ZINC03992788**	ant	-7.377609
**ZINC03993025**	ant	-7.319134

**ZINC03993032**	ant	-6.008025
**ZINC03993033**	ant	-4.450298
**ZINC03998920**	ant	-8.097624
**ZINC04000664**	ant	-5.825814
**ZINC04000719**	ant	-6.187756
**ZINC04001207**	ant	-5.718373
**ZINC04001221**	ant	-7.375793
**ZINC04001497**	ant	-6.43357
**ZINC04003903**	ant	-5.81396
**ZINC04004779**	ant	-4.379209
**ZINC04044749**	ant	-6.516868
**ZINC04046141**	ant	-6.336627
**ZINC04059647**	ant	-6.924696
**ZINC04083381**	ant	-7.514335
**ZINC04083781**	ant	-7.560529
**ZINC04083781**	ant	-7.547059
**ZINC04106552**	ant	-6.977375
**ZINC04120696**	ant	-5.177317
**ZINC04122233**	ant	-6.769152
**ZINC04126098**	ant	
**ZINC04126098**	ant	
**ZINC04126098**	ant	
**ZINC04126107**	ant	
**ZINC04126107**	ant	
**ZINC04126107**	ant	

**ZINC04139854**	ant		-5.685254
**ZINC04155216**	ant		-6.409469
**ZINC04155518**	ant		-6.353027
**ZINC04155518**	ant		-5.587727
**ZINC04157569**	ant		-5.700999
**ZINC04157569**	ant		
**ZINC04162875**	ant		-7.948236
**ZINC04172817**	ant		-5.490422
**ZINC04172817**	ant		-5.293268
**ZINC04184769**	ant		-5.804138
**ZINC04189343**	ant		-5.299741
**ZINC04192482**	ant		-6.528064
**ZINC04192482**	ant		-6.221249
**ZINC04217617**	ant		-6.786933
**ZINC04217682**	ant		-6.40945
**ZINC04265650**	ant		-7.901682
**ZINC04267997**	ant	-7.459519	-7.489492
**ZINC04279611**	ant		-7.590977
**ZINC04305622**	ant		-5.751211
**ZINC04347993**	ant		-5.607622
**ZINC04352901**	ant		-7.215993
**ZINC04357008**	ant		-5.782075
**ZINC04357454**	ant		-6.551497
**ZINC04357820**	ant		-6.53454
**ZINC04369054**	ant		-6.539281

**ZINC04370003**	ant		-5.710595
**ZINC04378885**	ant		-7.841971
**ZINC04378916**	ant		-4.986694
**ZINC04379032**	ant		-7.543301
**ZINC04383652**	ant		-8.678896
**ZINC04392605**	ant		-11.355314
**ZINC04392612**	ant		-11.522149
**ZINC04399803**	ant		-6.636417
**ZINC04399846**	ant		-6.565275
**ZINC04399920**	ant		-7.297228
**ZINC04400371**	ant		-6.899104
**ZINC04400560**	ant		-6.046067
**ZINC04400918**	ant		-7.880374
**ZINC04415504**	ant	-6.268183	-6.077447
**ZINC04416414**	ant		-6.840525
**ZINC04417656**	ant		-5.460063
**ZINC04419035**	ant		-8.015731
**ZINC04419114**	ant		-6.718259
**ZINC04422548**	ant		-8.052037
**ZINC04422548**	ant		-6.265371
**ZINC04423466**	ant		-6.615404
**ZINC04424334**	ant		-7.44844
**ZINC04425650**	ant		
**ZINC04425988**	ant		-7.622381
**ZINC04428158**	ant		-3.395056

**ZINC04439060**	ant	-7.751907
**ZINC04439705**	ant	-7.424189
**ZINC04456911**	ant	-7.141572
**ZINC04457331**	ant	-7.649275
**ZINC04462049**	ant	-5.581535
**ZINC04464920**	ant	-4.397052
**ZINC04465069**	ant	-5.364903
**ZINC04465170**	ant	-7.372219
**ZINC04465739**	ant	-5.11006
**ZINC04466276**	ant	-6.781783
**ZINC04467730**	ant	-5.059245
**ZINC04468591**	ant	-6.181853
**ZINC04470103**	ant	-5.041402
**ZINC04470111**	ant	-5.455297
**ZINC04470255**	ant	-5.713202
**ZINC04470268**	ant	-5.857424
**ZINC04470439**	ant	-5.644571
**ZINC04470449**	ant	-6.185725
**ZINC04470509**	ant	-6.216582
**ZINC04470514**	ant	-5.981798
**ZINC04474882**	ant	-6.607514
**ZINC04490961**	ant	-5.936021
**ZINC04492395**	ant	-8.730276
**ZINC04492404**	ant	-7.737252
**ZINC04502932**	ant	-8.427181

**ZINC04502986**	ant	-6.347334
**ZINC04508447**	ant	-7.119861
**ZINC04510086**	ant	-6.281068
**ZINC04513709**	ant	-7.629394
**ZINC04515753**	ant	-6.799207
**ZINC04518559**	ant	-6.679638
**ZINC04586759**	ant	-7.554003
**ZINC04593979**	ant	-7.354591
**ZINC04594366**	ant	-6.232759
**ZINC04595063**	ant	-7.824535
**ZINC04618418**	ant	-5.991127
**ZINC04620296**	ant	-10.159698
**ZINC04620611**	ant	-6.974812
**ZINC04622652**	ant	-7.239296
**ZINC04627349**	ant	-6.106165
**ZINC04627834**	ant	-7.387113
**ZINC04628129**	ant	-8.221471
**ZINC04628229**	ant	-6.925634
**ZINC04628995**	ant	-7.757632
**ZINC04631130**	ant	-7.402009
**ZINC04638710**	ant	-5.29672
**ZINC04638712**	ant	-6.120625
