# Supplementary material for: Competitive molecular docking approach for predicting estrogen receptor subtype α agonists and antagonists
Source: BMC Bioinformatics. 2014 Oct 21;15(Suppl 11):S4. doi: 10.1186/1471-2105-15-S11-S4 (PMC4251048; doi:10.1186/1471-2105-15-S11-S4)
Supplement: Additional file 4 — Chemical structures of crystallographic ligands [file 1471-2105-15-S11-S4-S4.pdf]

**Additional file 4** The chemical structures of the crystallographic ligands used in the first set of docking, labelled according to the ID listed in Table S1.

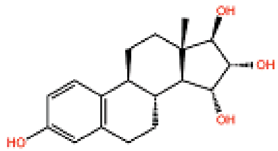

X1: 3L03

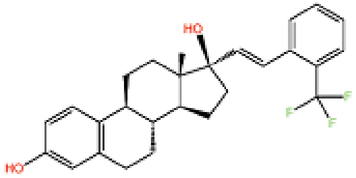

X2: 2P15

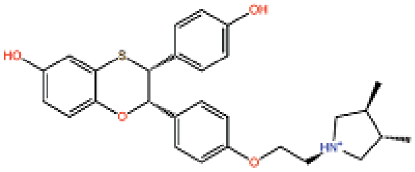

X3: 1XP6

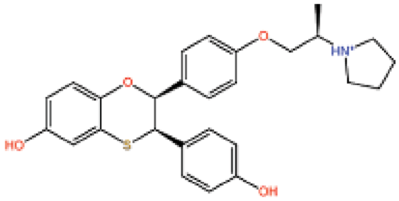

X4: 1XPC

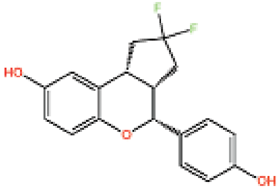

X5: 2Q70

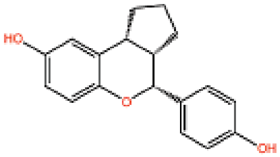

X6: 2I0J

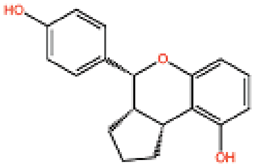

X7: 2POG

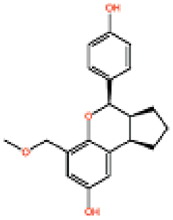

X8: 2QE4

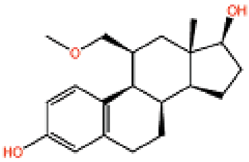

X9: 2QGT

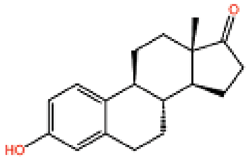

X10: 3HM1

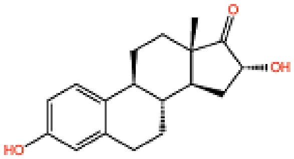

X11: 3HLV

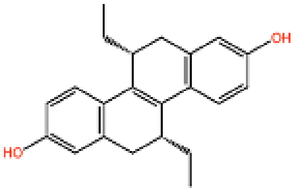

X12: 1L2I

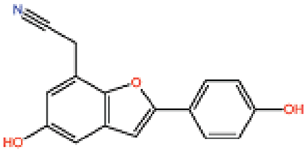

X13: 1X7E

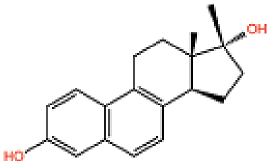

X14: 2B1Z

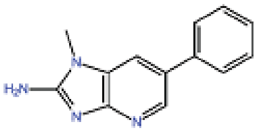

X15: 2QXM

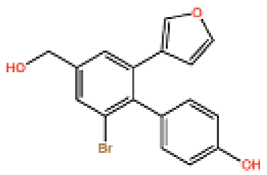


X16: 4DMA

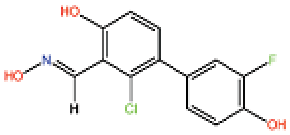


X17: 4IWF

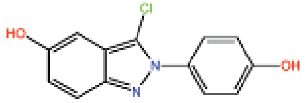


X18:2QGW

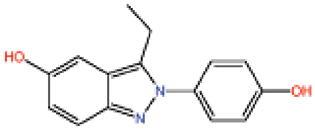


X19: 2QAB

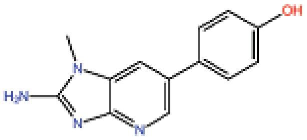


X20: 2QSE

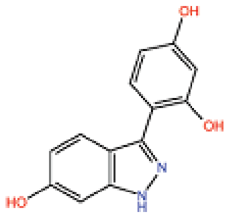


X21: 2QA6

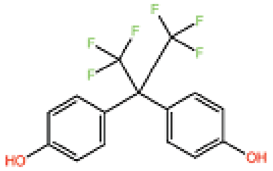


X22: 3UUA

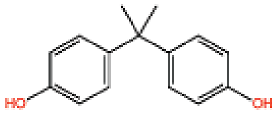


X23: 3UU7

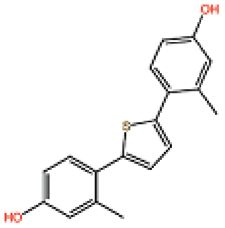


X24: 4IWC

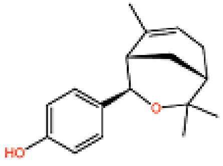


X25: 2G44

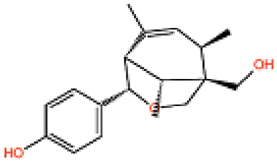


X26: 1ZKY

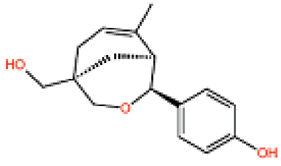


X27: 2B1V

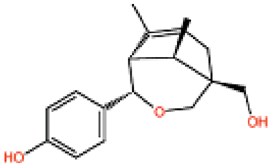


X28: 2FAI

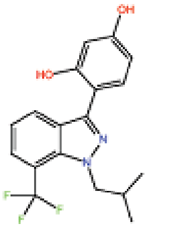


X29: 4IV2

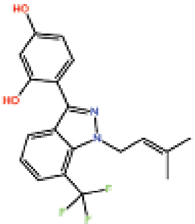


X30: 3OSA

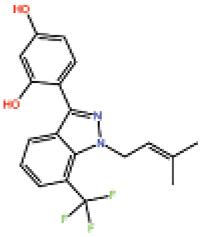

X31: 4IW8

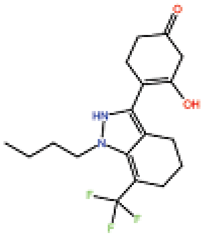

X32: 4IVY

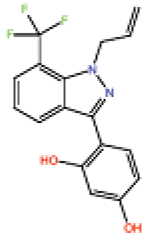

X33: 3OS9

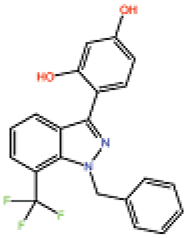

X34: 3OS8

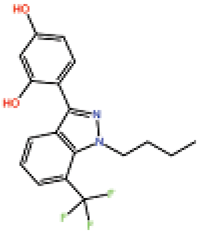

X35: 4IUI

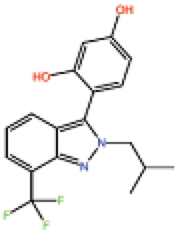

X36: 4IV4

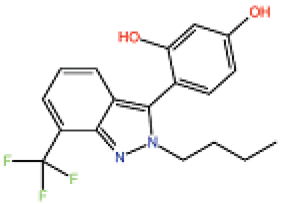

X37: 4IW6

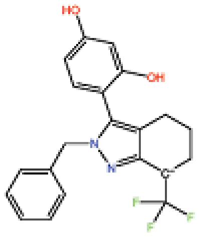

X38: 4IVW

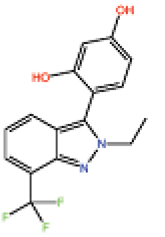

X39: 4IU7

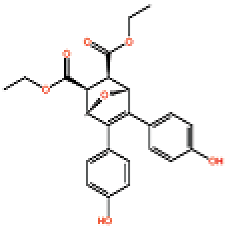

X40: 2QH6

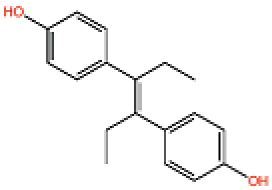

X41: 3ERD

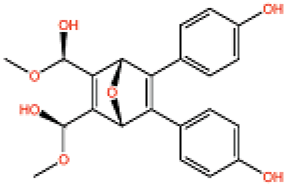

X42: 2QR9

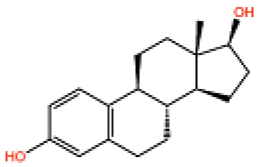

X43: 1GWR

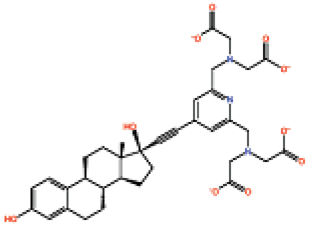

X44: 2YAT

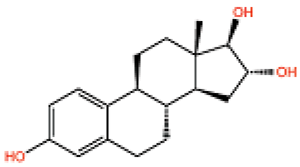

X45: 3Q95

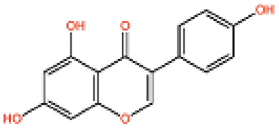

X46: 2QA8

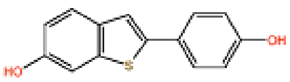

X47: 1GWQ

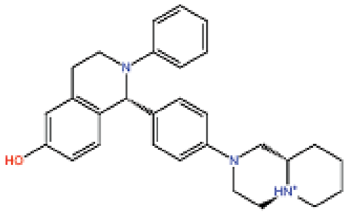

X48: 1XQC

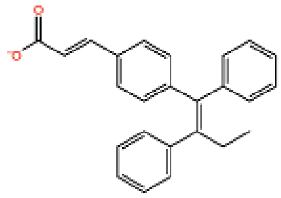

X49: 1R5K

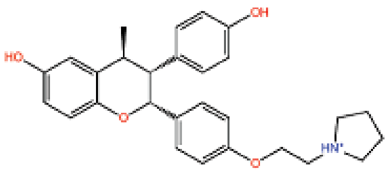

X50: 1YIM

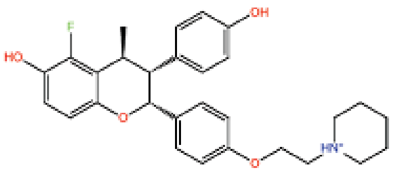

X51: 1YIN

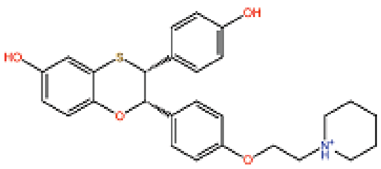

X52: 1SJ0

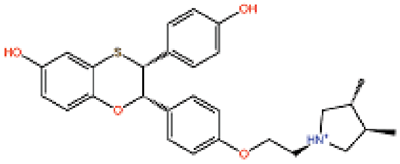

X53: 1XP1

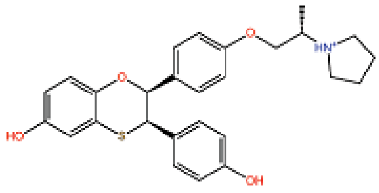

X54: 1XP9

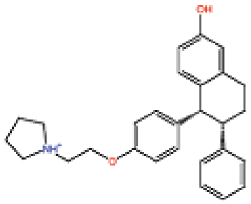

X55: 2OUZ

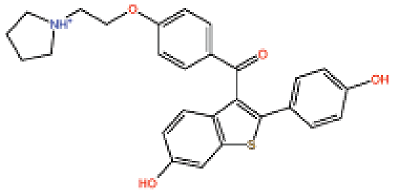

X56: 2R6Y

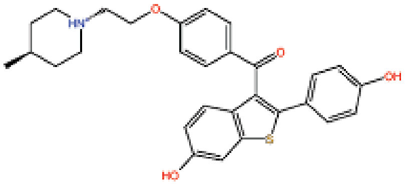

X57: 2R6W

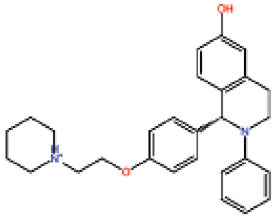

X58: 1UOM

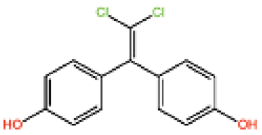

X59: 3UUC

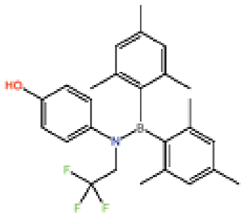

X60:2Q6J

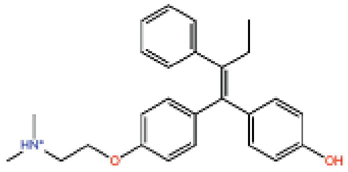

X61: 3ERT

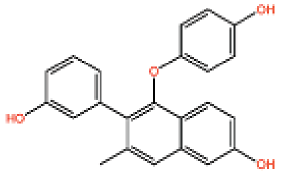

X62: 3DT3

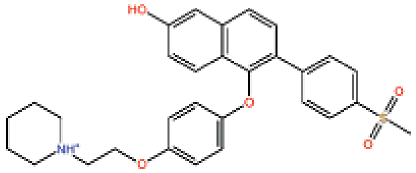

X63: 2AYR

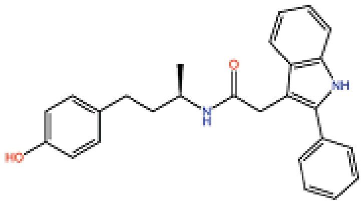

X64: 2IOK

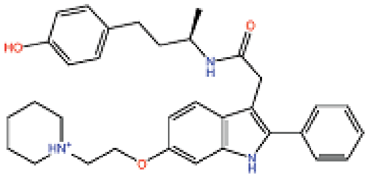

X65: 2IOG

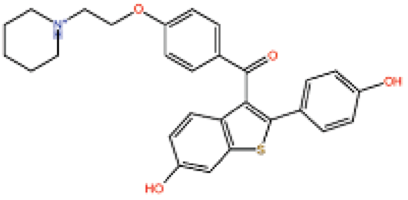

X66: 2QXS
